# COVID‐19 Pandemic: A Comprehensive Meta‐Review of Global Impacts, Responses, and Future Preparedness

**DOI:** 10.1111/crj.70134

**Published:** 2025-11-21

**Authors:** Rulin Wang, Muhammad Ahsan Naeem

**Affiliations:** ^1^ Department of Nurses at Medical College Xijing University Xi'an Shaanxi China; ^2^ Department of Nursing, Faculty of Medicine and Health Sciences MAHSA University Jenjarom Selangor Malaysia; ^3^ Department of Basic Sciences (Pharmacology) University of Veterinary and Animal Sciences Lahore Pakistan

**Keywords:** COVID‐19 pandemic, global health impact, meta‐analysis, pandemic preparedness, public health response

## Abstract

**Introduction:**

The COVID‐19 pandemic due to SARS‐CoV‐2 has initiated historically unparalleled global health, social, and economic impacts. Syntheses of the multivariable interdependent effects on the multiple clinical, immunologic, psychosocial, and health service realms are required to guide current and future public health preparedness and policy.

**Methods:**

A systematic review of a meta‐analysis was conducted using the PubMed, Scopus, and Web of Science databases from December 2019 to 2025 to identify observational studies and randomized controlled trials. Quantitative reporting studies of COVID‐19 outcomes were included. Random‐effects model aggregated effect sizes were estimated and tested for heterogeneity with Cochran's Q, *τ* ^2^, and *I* ^2^. Subgroup, moderator, publication bias, sensitivity, and leave‐one‐out analyses were conducted for exploration and validation.

**Results:**

Twenty‐four studies were classified into three categories: clinical outcomes (15 studies), immunogenicity (4 studies), and psychosocial/health service outcomes (5 studies). There was no statistically significant pooled effect (effect ratio 0.95, 95% CI: 0.55–1.62) with severe heterogeneity (*I* ^2^ > 99%). Immunogenicity showed a statistically significant positive effect (pooled estimate 0.77, 95% CI: 0.38–1.16) with high heterogeneity (I^2^ ~96%). Psychosocial effects were highly heterogeneous with non‐significant overall effects (estimate −1.03, 95% CI: −5.74 to 3.69). Sample size was an influential moderator that explained significant between‐group heterogeneity.

**Discussion:**

The outcomes reveal robust immunogenic vaccine impacts and indeterminate psychosocial and clinical impacts, consistent with the heterogeneity and complexity of COVID‐19 effects. Great heterogeneity highlights methodological standardization and cautious interpretation. This present meta‐analysis offers key lessons to guide subsequent study design and manufacture of fair health policy and pandemic readiness.

## Graphical abstract

Graphical abstract depicting the global timeline of the COVID‐19 pandemic, illustrating key stages such as virus emergence, public health measures, quarantine, vaccination, clinical response, and their impacts on mortality, mental health, social disparities, and meta‐analytic insights for future recommendations.

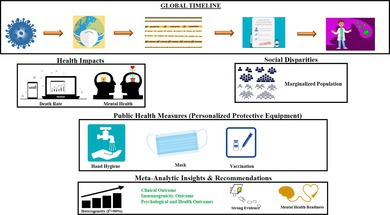

## 1 Introduction

The new coronavirus SARS‐CoV‐2 pandemic, known to cause COVID‐19, is one of the most unprecedented global health catastrophes in recent times [1]. The virus has spread at light speed across the globe since its occurrence in late 2019 and caused disastrous morbidity and mortality, closed down healthcare facilities, interrupted economies, and transformed social habits globally [2]. Governments, public health organizations, and communities have had to act quickly with a suite of public health interventions, from lockdown and travel measures to mass testing, contact tracing, and record vaccination drives [3]. But the pandemic has also laid bare pre‐existing gaps in international preparedness and response capacities [4]. In addition to its direct clinical and epidemiologic impact, the COVID‐19 pandemic has revealed structural disparities in access to and quality of health care [5]. The inequalities have been most stark between marginalized and vulnerable groups, such as racial and ethnic minorities, individuals from low‐income countries, people with long‐term health conditions, and front‐line healthcare workers [6]. Furthermore, the crisis has precipitated long‐term secondary effects like mental health decline, delayed treatment of non‐COVID conditions, economic insecurity, disruption of education, and pressure on essential services [7]. As the world slowly moves towards a post‐pandemic world, it is important to critically summarize the increasing volume of evidence that has been accumulated over the past 5 years [8]. Various studies, reviews, and policy analyses have captured various aspects of the expansion and impact of the pandemic, and the effectiveness of various interventions [9]. However, there is still a gap in bringing these results together into a general understanding that can be utilized in planning future preparedness, resilience, and policy [10]. This meta‐review bridges this gap by aggregating and evaluating high‐quality evidence from systematic reviews, meta‐analyses, and scoping reviews during the period between December 2019 and May 2025 comprehensively. It attempts to measure the public health, social, and economic effects of COVID‐19 on the entire world; evaluate public health responses' effectiveness; contrast differences in results; and extract lessons for future pandemic preparedness. By giving an integrated, evidence‐based overview, this review will help to support policy‐making using evidence and promote equitable and sustainable health systems for future global health crises.

## 2 Methodology

### 2.1 Search Strategy and Study Selection

A comprehensive literature search was performed using the following search query on key databases including PubMed, Scopus, and Web of Science (WOS): (((COVID‐19 OR SARS‐CoV‐2 OR Coronavirus AND impact OR effect OR consequence OR burden) AND (response OR intervention OR policy OR public health OR mitigation)) AND (preparedness OR resilience OR future readiness OR response planning)) AND (disparities OR inequities OR vulnerable populations OR low‐income countries OR Global South). The search for literature included articles between December 2019 and 2025 and excluded English‐language articles. Duplicates were removed prior to screening. Records were screened in two phases—first by title and abstract, and then by full‐text—based on predefined eligibility criteria, on primary research studies with randomized controlled trials (RCTs) and observational study design with quantitative outcomes of COVID‐19 effects and responses. The PRISMA flow diagram sets out the study selection process, for example, the number of records identified, screened, excluded with reasons, and finally included in quantitative meta‐analyses. The strict search strategy achieved comprehensive coverage of eligible studies within the scope of the review.

### 2.2 Inclusion and Exclusion Criteria

#### 2.2.1 Inclusion Criteria

The studies were depicted to have inclusion criteria in achieving quality, along with contextually relevant evidence for COVID‐19. The studies were mainly primary research studies, which were RCTs and observational studies, including cohort, case–control, cross‐sectional, and longitudinal studies with well‐defined methodologies. The surveyed population involved human subjects with clearly established sample sizes and descriptions from all over the world, including the developing world in general and the Global South specifically, to produce varied impacts. Research must respond to headline COVID‐19‐specific issues, such as health, economic, and social impacts; responses, such as interventions and policy; preparation activity, such as planning and resilience; and equity issues, such as inequalities and vulnerable groups. For inclusion, studies must have had adequate quantitative or statistical data reported in the form of prevalence rates, odds ratios (ORs), risk ratios (RRs), hazard ratios, regression coefficients, effect sizes, confidence intervals (CIs), or *p*‐values that are quantitatively extractable for valid meta‐analysis. Published English versions in full published after December 2019, when the COVID‐19 virus erupted across the globe, were only considered on the grounds of currency and availability. Finally, research must have been significantly relevant to the SARS‐CoV‐2 COVID‐19 pandemic. Very rigorous inclusion criteria were utilized so that included studies were sound methodologically, contextually appropriate, and reported data that were quantitative in nature so that aggregate results and conclusions of the review would become increasingly reliable and relevant.

#### 2.2.2 Exclusion Criteria

Exclusion criteria were used to allow only high‐quality, applicable studies with sufficient quantitative data to be aggregated. Reviews, systematic reviews, scoping reviews, meta‐analyses, protocols, commentaries, editorials, theses, and dissertations were excluded because they do not report primary research findings. Case reports or series with fewer than 10 patients were excluded in order to reject studies of low generalizability. Purely qualitative research, excluding quantitative or statistical data, was excluded because the emphasis was placed on meta‐analysis of numeric data. Laboratory, in vitro, or in silico studies from non‐human or virtual data were excluded to keep emphasis on research with human populations. Quantitative synthesis relies on extractable statistical data, and therefore research without them was excluded. Non‐available or non‐English full‐text studies were excluded in order to allow for thorough evaluation and data extraction. Furthermore, non‐COVID‐19‐related studies or only pre‐pandemic data reports were eventually excluded in order to allow for direct usability within the SARS‐CoV‐2 pandemic scenario. These standards were set to facilitate methodological quality, quantitative analysis suitability of the data, and human‐oriented research, especially within the context of COVID‐19.

### 2.3 Data Extraction

Data were systematically withdrawn from all of the included studies by using a pre‐determined template to guarantee completeness and consistency. Data withdrawn included authors' names, publication years, and journals where the study was published. Study population details and the type of interventions studied were also recorded. The type of outcome that was measured, i.e., COVID‐19‐related death, mental health impact, or vaccine uptake, was also noted. For each outcome, the corresponding effect size and statistic that were recorded, e.g., ORs, RRs, adjusted ORs (aORs), or incidence proportions. Related CIs and log‐transformed effect sizes were recorded where possible. Sample sizes or numbers of participants included in the analysis were reported when available. These data were the basis for pooled meta‐analysis and facilitated subgroup analyses in the review.

### 2.4 Quality Assessment

Methodological quality and risk of bias in studies included in this meta‐analysis were systematically assessed with the help of two validated tools appropriate for study design. The studies involve a mix of observational cohorts and RCTs using tools like ROBINS‐I for observational studies and RoB 2 for clinical trials. The assessment considers issues like confounding, participant selection, categorization of interventions or exposures, deviation from scheduled interventions, missing data management, measurement of outcomes, and selection bias in published results. Each study is critically assessed on these areas to determine an overall risk of bias that ranges from low to severe. Data collection methods vary—from consecutive recruitment in hospital cohorts from several countries to blinding and randomization in clinical trials. Outcomes are quantified against mortality, immunogenicity, hospitalization, and psychological scoring as defined by standardized protocols and validated assays.

### 2.5 Group‐Wise Meta‐Analysis and Rationale for Grouping

Group A comprises 15 studies that assessed clinical outcomes, including mortality, infection, hospitalization, adherence, and equity‐related risks. None of the studies had statistically significant pooled effect sizes, with extremely high heterogeneity between studies. The studies included provided a wide‐ranging assessment of the effects of COVID‐19 on clinical outcomes across various settings, with consistent statistical analysis indicating variability and a non‐conclusive effect. Group B comprises four immunogenicity studies examining neutralizing antibodies, seroconversion, and associated immunologic parameters. Such studies revealed statistically significant pooled effects demonstrating favorable immunogenic responses with high heterogeneity, consistent with heterogeneity in vaccine types and participants' characteristics. Group C comprises five studies assessing psychosocial factors and health service outcomes through different quantitative approaches. The meta‐analysis for this group had no statistically significant effect and very high heterogeneity with suggestions of possible publication bias, reflecting the complexity of psychosocial and service outcomes during the pandemic. The logical basis for dividing the data into three groups (clinical outcomes, immunogenicity outcomes, and psychosocial and health service outcomes) was to categorize the wide variety of studies into groups depending on their main outcome focus and methodological features. This division facilitates more accurate and sensible meta‐analytic synthesis according to the nature of the respective outcome, responding better to heterogeneity as well as facilitating clearer interpretation of the COVID‐19 pandemic's compound effects.

### 2.6 Meta‐Analytic Method

The meta‐analysis was conducted using a systematic method to combine evidence from the included studies. A random‐effects model was employed to account for expected heterogeneity among studies, which varied in terms of populations, interventions, and study designs. Individual study summary effect measures were then pooled to provide an overall estimate with 95% CIs. Between‐study heterogeneity was measured with statistical estimates such as Cochran's Q test, tau‐squared (*τ* ^2^) for the variation between studies, and the (*I* ^2^) statistic as a percentage of variation due to heterogeneity rather than chance. Subgroup analysis by study features, such as sample size, was performed to investigate the causes of heterogeneity. Publication bias was examined to determine whether unpublished or missing studies would have any possible impact on the meta‐analytic finding.

### 2.7 Statistical Analysis

Statistical inferences were drawn through the use of established meta‐analytic computer programs. The random‐effects model and the REML estimator of (*τ* ^2^) estimated pooled effect sizes. Standard errors (SEs) and pooled estimates were generated. *Z*‐tests and respective *p*‐values provided precision and significance of pooled effects. Cochran's Q tested for heterogeneity, and (*I* ^2^) measured heterogeneity. Publication bias was confirmed with funnel plots and weighted regression tests. Sensitivity analyses entailed excluding individual studies systematically to determine the stability of the results. Statistical significance was evaluated at a *p*‐value threshold of 0.05. Deliberate investigation of heterogeneity and effect modifiers was conducted via subgroup and meta‐regression analysis as necessary to maintain the validity and reliability of findings.

## 3 Results

### 3.1 Study Selection and Screening Process

**FIGURE 1:**
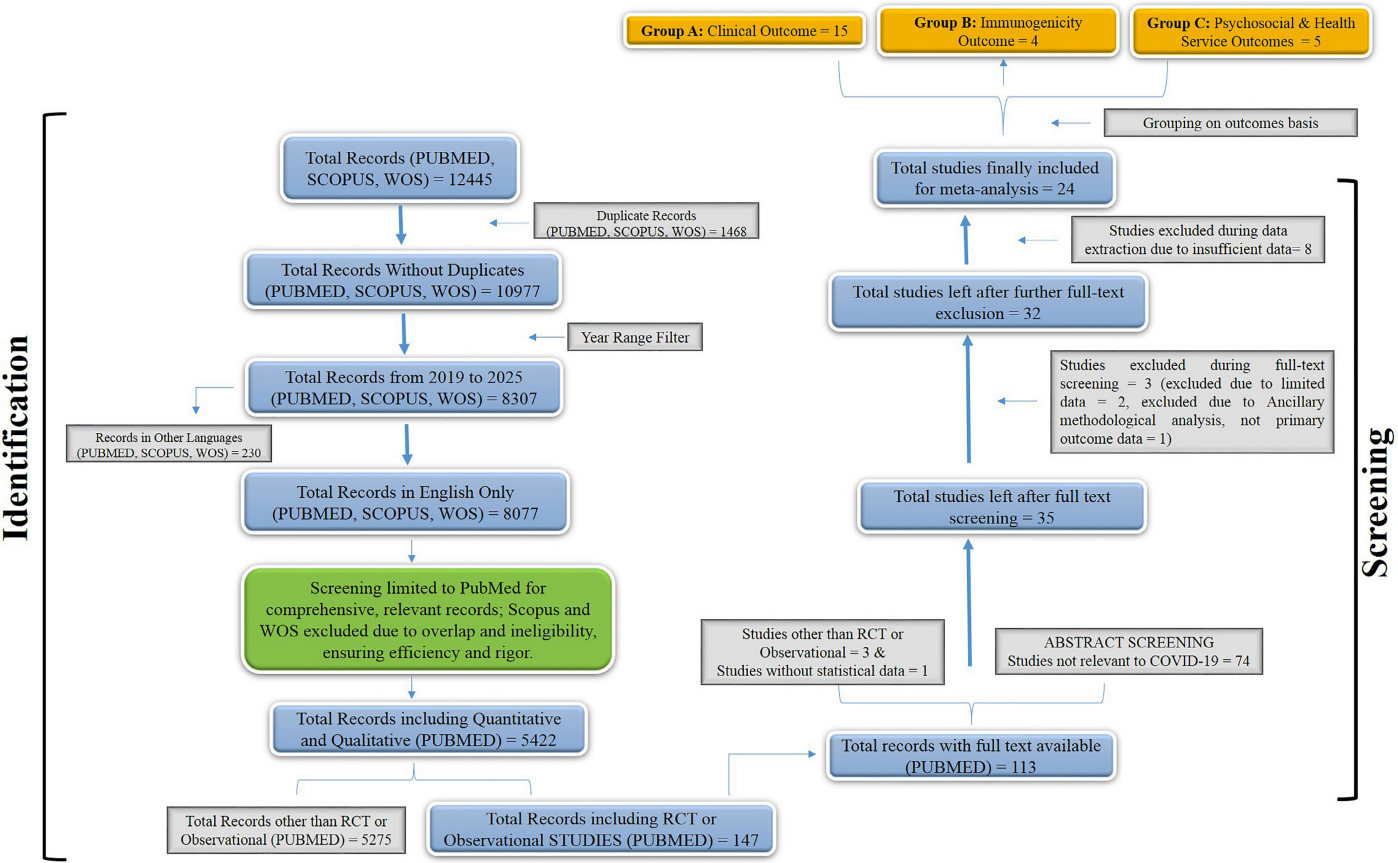
PRISMA flow diagram of record identification, screening, eligibility, and inclusion process for the systematic review and meta‐analysis. Record numbers at each step and specific exclusion reasons are given. Such a detailed display is aimed at offering more transparency and reproducibility of the study selection process.

Systematic search across various databases such as PubMed, Scopus, and WOS first yielded 12 445 records, from which 1468 were duplicates and were eliminated, leaving behind 10 977 unique records. English‐language (8077) publications for the period 2019–2025 were given precedence, with screening limited largely to PubMed in order to provide methodological quality without sacrificing feasibility. Following title and abstract screening, 35 studies were selected for full‐text evaluation and consisted of RCTs and observational research of COVID‐19 impacts, response, readiness, and equity in at‐risk populations and low‐resource environments. Upon full‐text screening, three studies were excluded because of bad data or non‐primary outcomes, and an additional eight were excluded on data extraction because there was a deficit in quantitative data. Finally, 24 studies were pooled into a quantitative synthesis (meta‐analysis). The studies varied by design, population, and outcome measures, covering clinical, immunologic, and psychosocial and health services areas, which enabled extensive subgroup analyses for the purpose of adjusting for heterogeneity and distinct research hypotheses. The PRISMA flow diagram (Figure 1) outlines the sequential study selection. The detailed description of all studies for their inclusion and exclusion, and their Google Scholar searchable links are provided in Data S1. The characteristics features of these 24 studies are presented in tabulated form in Data S2.

### 3.2 Qualitative Valuation Results

For non‐randomized observational studies (*n* = 15), the ROBINS‐I scale (Risk Of Bias In Non‐randomized Studies—of Interventions) was utilized, which assesses regions of bias in a systematic fashion, like confounding, participant selection, intervention/exposure classification, deviations from intended intervention, missing data, measurement of outcome, and selective reporting. Risk of bias assessments ranged from low to very serious, and the majority of studies represented a moderate to very serious risk of residual confounding, selection biases, and heterogeneity of exposure classification or loss of data. Randomized trials (*n* = 9) were assessed with the application of the Cochrane Risk of Bias 2 (RoB 2) tool for the randomization process, deviations from the intended intervention, missing outcome data, measurement of outcome, and selective reporting. Most RCTs were classified as having some concern to low risk of bias, and common limitations in the form of weak blinding and selective outcome reporting were noted. These analyses reflect study methodological quality inconsistency between included studies. Subgroup analyses within risk of bias categories were conducted to minimize this, and sensitivity and leave‐one‐out analyses were conducted to assess the effect of study quality on the pooled evidence. This approach increases the validity and interpretability of meta‐analytic findings on the basis of statistical adjustment against evidence‐based heterogeneity and potential bias. Aggregate quality assessment for 24 included studies and risk of bias judgments specific to the domain using ROBINS‐I and RoB 2 tools are presented in Data S3.

### 3.3 Quantitative Synthesis (Meta‐Analytic Findings)

**FIGURE 2:**
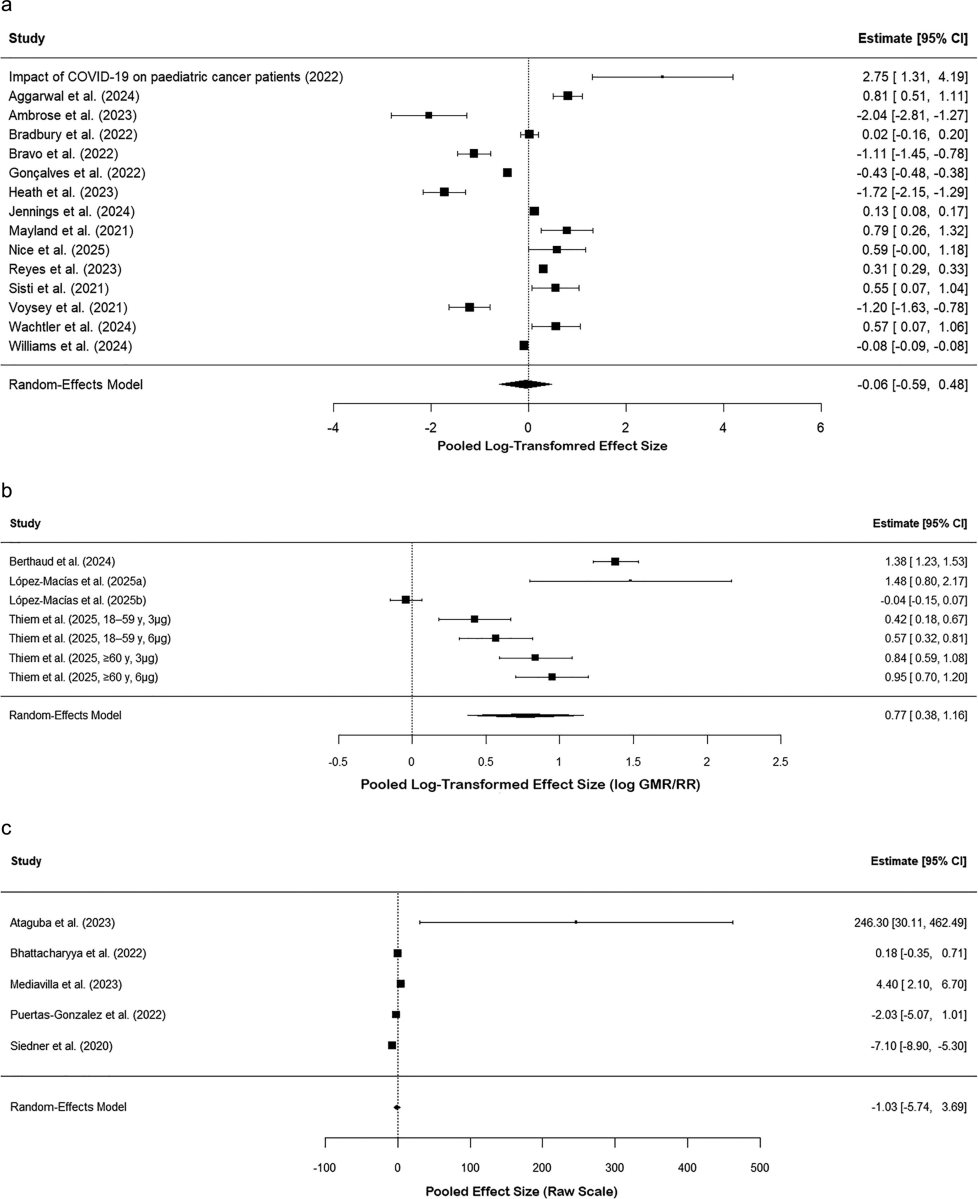
(a) Forest plot of pooled effect sizes and 95% confidence intervals for clinical outcomes of mortality, infection, hospitalization, adherence, and equity risks from 15 studies. (b) Forest plot of pooled immunogenicity outcomes (neutralizing antibodies, seroconversion, GMT/GMR) with study estimates and confidence intervals. (c) Forest plot of psychosocial and health service outcomes with effect sizes and confidence intervals from 5 studies, showing high heterogeneity.

The detailed meta‐analysis with all statistical analysis for three groups is presented in Data S4. Under Group A, the meta‐analysis included 15 trials whose clinical outcomes were measured as mortality, infection, hospitalization, adherence, and risks to equity. The random‐effects model based on restricted maximum likelihood (REML) estimation was applied. The log‐transformed effect size pool was −0.0551, and the SE was 0.2732. On back‐transformation to the original scale, this represented a ratio of 0.95 (95% CI: 0.55–1.62). The overall effect was not statistically significant (*p*‐value = 0.84) with no discernible overall measurable effect from the included studies. As shown in the forest plot (Figure 2a), the pooled effect size for clinical outcomes did not reach statistical significance. The very broad CI encompassing 1 is indicative of uncertainty and suggests no obvious benefit or harm of the exposures or interventions under investigation. There were four studies in Group B with immunogenicity outcomes such as neutralizing antibodies, seroconversion rates, and geometric mean titers/ratios (GMT/GMR). In a random‐effects model, the overall pooled effect estimate was 0.77 (95% CI: 0.38–1.16) and significant at *p* = 0.0001. The result indicates a composite positive effect on outcomes in domains of immunogenicity. There was very high heterogeneity (*I* ^2^ = 95.95%), which is indicative of high heterogeneity of study effect sizes and the need for additional subgroup or moderator analyses to establish reasons for variation. Figure 2b presents the forest plot summarizing immunogenicity outcomes across included studies, showing a statistically significant pooled effect. In Group C, the meta‐analysis of five studies with psychosocial and health service outcomes provided a global pooled effect of −1.03 (95% CI: −5.74 to 3.69) with a random‐effects model. The statistical effect was not significant (*p* = 0.67). Extremely high heterogeneity (*I* ^2^ = 95.84%) with extremely high between‐study variance (*τ* ^2^ = 21.9993) was found with high study outcome variability. The forest plot (Figure 2c) for psychosocial outcomes illustrates substantial heterogeneity and wide CIs. This great heterogeneity makes drawing conclusions with absolute confidence on the global impact on psychosocial, as well as health service‐related outcomes, challenging. On average, whereas pooled immunogenicity effects in Group B were statistically different from zero, excessive heterogeneity noted across all groups is an unprecedented level of variation in study groups, outcome definitions, interventions, as well as measurement tools, typical of meta‐analyses in different study contexts. This heterogeneity recognizes that the real effects differ across study settings instead of estimating a single common effect size. Pooled estimates hence demand cautious interpretation. We alleviated this by conducting strict ancillary analyses, such as subgroup and moderator analyses, to probe the sources of heterogeneity and more rigorously test variations in effect size. The meta‐analysis summary of findings on the different groups of COVID‐19 effects, consisting of combined effect sizes, heterogeneity measures, and *p*‐values, is reported in Table 1. Here, some of the key quantitative data are presented to support the interpretations described in the results section.

**TABLE 1:** Summary of meta‐analysis results for COVID‐19 impact groups.

Outcome group	Number of studies (k)	Pooled effect size (log scale)	Standard error (SE)	95% confidence interval	*p*‐value	*I* ^2^ (%)	*τ* ^2^ (between‐study variance)	Interpretation
Clinical outcomes (A)	15	−0.0551	0.2732	−0.5907 to 0.4804	0.840	99.94	1.0552	No significant effect; extreme heterogeneity
Immunogenicity outcomes (B)	4	0.77	0.20	0.38–1.16	0.0001	95.95	0.256	Significant positive effect; high heterogeneity
Psychosocial outcomes (C)	5	−1.03	2.40	−5.74 to 3.69	0.67	95.84	21.9993	Non‐significant; extremely high heterogeneity

### 3.4 Data Characteristics and Subgroup Analysis

Fifteen studies in Group A (clinical outcomes) were included, exhibiting highly excessive heterogeneity (*I* ^2^ = 99.94%) and substantial between‐study heterogeneity (*τ* ^2^ = 1.0552, SE = 0.4219), which justified the use of a random‐effects model. Sample size subgroup analysis divided the studies into three groups: small (two studies), medium (four studies), and large (nine studies). Heterogeneity remained high, especially in large samples (*I* ^2^ = 96.13%), but 33.95% heterogeneity was accounted for by sample size (*R* ^2^ = 33.95%). In Group B (outcomes of immunogenicity), there were four high‐heterogeneity (*I* ^2^ = 95.95%) and moderate‐variance (*τ* ^2^ = 0.2558, SE = 0.1607) studies. Subgroup analyses were by vaccine dose and sample size, and age, and explained 82.65% of heterogeneity (*R* ^2^ = 82.65%) with residual *I* ^2^ = 88.12%. The effects were significant in small studies (estimate = 1.48, *p* = 0.001) but were smaller in large studies (estimate = −1.52, *p* = 0.0046). Group C (psychosocial and health service outcomes) had five studies with highly high heterogeneity (*I* ^2^ = 95.84%) and vast variance (*τ* ^2^ = 21.9993, SE = 18.8963). Sample size as a moderator explained 94.5% of heterogeneity (*R* ^2^ = 94.5%) with residual heterogeneity collapsed into *I* ^2^ = 32.7%, making subgroup analysis to fit variability possible.

### 3.5 Heterogeneity

Within group A, there was high heterogeneity between studies, with tau‐squared (*τ* ^2^) = 1.0552 (SE = 0.4219) and *I*‐squared (I^2^) = 99.94%, which represents almost all the observed variation in effect sizes resulting from real differences instead of chance. Cochran's Q‐test was highly significant (Q(14) = 1668.86, *p* < 0.0001), proving significant inconsistency among studies. This extreme heterogeneity means that the pooled effects must be interpreted with caution, and subgroups or moderators must be examined. Between‐study variation was high but moderate in group B, with tau‐squared (*τ* ^2^) being estimated at 0.256 (SE = 0.161). The statistic I‐squared was 95.95%, meaning that practically all variation observed occurred due to true differences rather than due to sampling error. Cochran's Q‐test was highly significant (Q(6) = 251.48, *p* < 0.0001), supporting the occurrence of significant heterogeneity among immunogenicity results reported. Between‐study heterogeneity in group C was extremely high, and *τ* ^2^ was estimated to be 21.9993 (SE = 18.8963), reflecting a very large true effects' variance. *I* ^2^ measure of 95.84% and Q‐test (Q(4) = 80.17, *p* < 0.0001) confirmed this high heterogeneity. The high *H* ^2^ (24.02) highlighted a huge difference between sampling and total variability, confirming that differences among studies are not by chance alone.

### 3.6 Subgroup (Moderator) Analysis of Sample Size Effects

In group A, moderator analysis grouped studies by sample size, which accounted for 34% of between‐study variance (*R* ^2^ = 33.95%). Small studies (*k* = 2) were heterogeneous with no effect (estimate = 0.69, *p* = 0.066). Medium‐sized studies (*k* = 4) had significant heterogeneity (*I* ^2^ = 75.4%) with a positive but non‐significant effect estimate (0.82, *p* = 0.079). Large studies (*k* = 9) exhibited high heterogeneity (*I* ^2^ = 96.1%) and borderline negative effects (estimate = −0.52, *p* = 0.062), suggesting potential sample size effects on direction of effects but with inconclusive significance. Moderator analysis in group B, where sample size was evaluated as a categorical variable, revealed that sample size accounted for an extensive proportion (82.65%) of heterogeneity. The model had significant overall moderator effects (QM(2) = 13.65, *p* = 0.0011). Small trials (< 100 patients) had a very strong positive influence (estimate = 1.48, *p* = 0.0010), while moderate‐sized trials (100–499 patients) were not significantly different from small trials (*p* = 0.54). Large trials (≥ 500 patients) had a significantly lower influence than small trials (estimate = −1.52, *p* = 0.0046), suggesting that study size greatly affects the size of immunogenicity results and explains much of the heterogeneity seen between studies. In group C, sample size moderation had a significant effect on the pooled effects and accounted for 94.5% of heterogeneity (*R* ^2^). The intercept (small studies) had a non‐significant estimate (−0.40, *p* = 0.68). Medium studies had a significant positive difference (+4.85, *p* = 0.01), whereas large studies had a significant negative effect (−6.70, *p* < 0.001). Residual heterogeneity fell to moderate levels (*τ* ^2^ = 1.21, *I* ^2^ = 32.7%), establishing that sample size is a robust moderator of variance between studies. Moderator analysis results by sample size categories between the COVID‐19 outcome groups are reported in Table 2 with varying effect sizes and heterogeneity patterns. Table 2 provides important quantitative evidence on the extent to which sample size moderates the pooled effects observed.

**TABLE 2:** Moderator analysis by sample size for COVID‐19 outcome groups.

Outcome group	Sample size category	Number of studies (k)	Pooled effect size (log)	SE	95% confidence interval	*p*‐value	Heterogeneity *I* ^2^ (%)	Notes/interpretation
Clinical outcomes (A)	Small (< 1 k)	2	0.69	0.38	−0.046 to 1.433	0.066	0%	No heterogeneity; borderline significance
	Medium (1–10 k)	4	0.82	0.46	−0.096 to 1.729	0.079	75.4%	Significant heterogeneity; positive but NS effect
	Large (> 10 k)	9	−0.52	0.28	−1.076 to 0.027	0.062	96.1%	High heterogeneity; borderline negative effect
Immunogenicity outcomes (B)	Small (< 100)	X	1.48	0.45	0.60–2.36	0.001	—	Strong positive effect for small studies
	Moderate (100–499)	X	−0.31	0.50	−1.28 to 0.67	0.54	—	Not significantly different from small studies
	Large (≥ 500)	X	−1.52	0.54	−2.57 to −0.47	0.005	—	Significantly lower effect compared with small trials
Psychosocial outcomes (C)	Small	X	−0.40	0.97	−2.30 to 1.51	0.68	—	Non‐significant
	Medium	X	4.85	1.88	1.17–8.53	0.01	—	Significant positive effect
	Large	X	−6.70	1.73	−10.09 to −3.31	< 0.001	—	Significant negative effect

#### 3.6.1 Subgroup Analysis by Effect Type

Subgroup analysis by effect direction further revealed heterogeneous patterns across the three groups of outcomes. In clinical outcomes (Group A), sample size moderately mitigated heterogeneity, with minimal evidence of departure from the overall effect in small studies (< 1000 participants), while large studies (> 10 000 participants) had a borderline reduced effect estimate (estimate = −1.28, *p* ≈ 0.06). However, extremely high heterogeneity was uncovered among subgroups, particularly in medium‐ and large‐sample sizes, indicating extremely high variability of effect sizes. In immunogenicity outcomes (Group B), the sample size of studies was an excellent moderator that accounted for approximately 83% of heterogeneity (*R* ^2^ = 82.65%).

Small trials (< 100 participants) had a very large and significant positive effect (estimate = 1.48, *p* = 0.0010), whereas large trials (≥ 500 participants) had significantly smaller effects (estimate = −1.52, *p* = 0.0046). Moderate‐sized studies (100–499) were indistinguishable from small studies. This suggests that the small trials will publish bigger effects of immunogenicity than the large trials. For psychosocial and health service outcomes (Group C), the sample size accounted for heterogeneity significantly too. The medium studies had a significant positive effect (estimate = 4.85, *p* = 0.01), but the large studies had a significant negative effect (estimate = −6.70, *p* < 0.001). The small study's intercept was not significantly different from zero. Subgroup analysis reduced residual heterogeneity by a large extent (*I* ^2^ decreased from 95.84% to 32.7%) and explained 94.5% of between‐study variability, indicating that sample size is a common source of effect estimates in this subgroup. Overall, subgroup analyses support the evidence that sample size is a strong moderator by outcome type because small studies produce larger effect sizes in all types universally, and large studies produce smaller or zero effects.

### 3.7 Publication Bias Assessment

**FIGURE 3:**
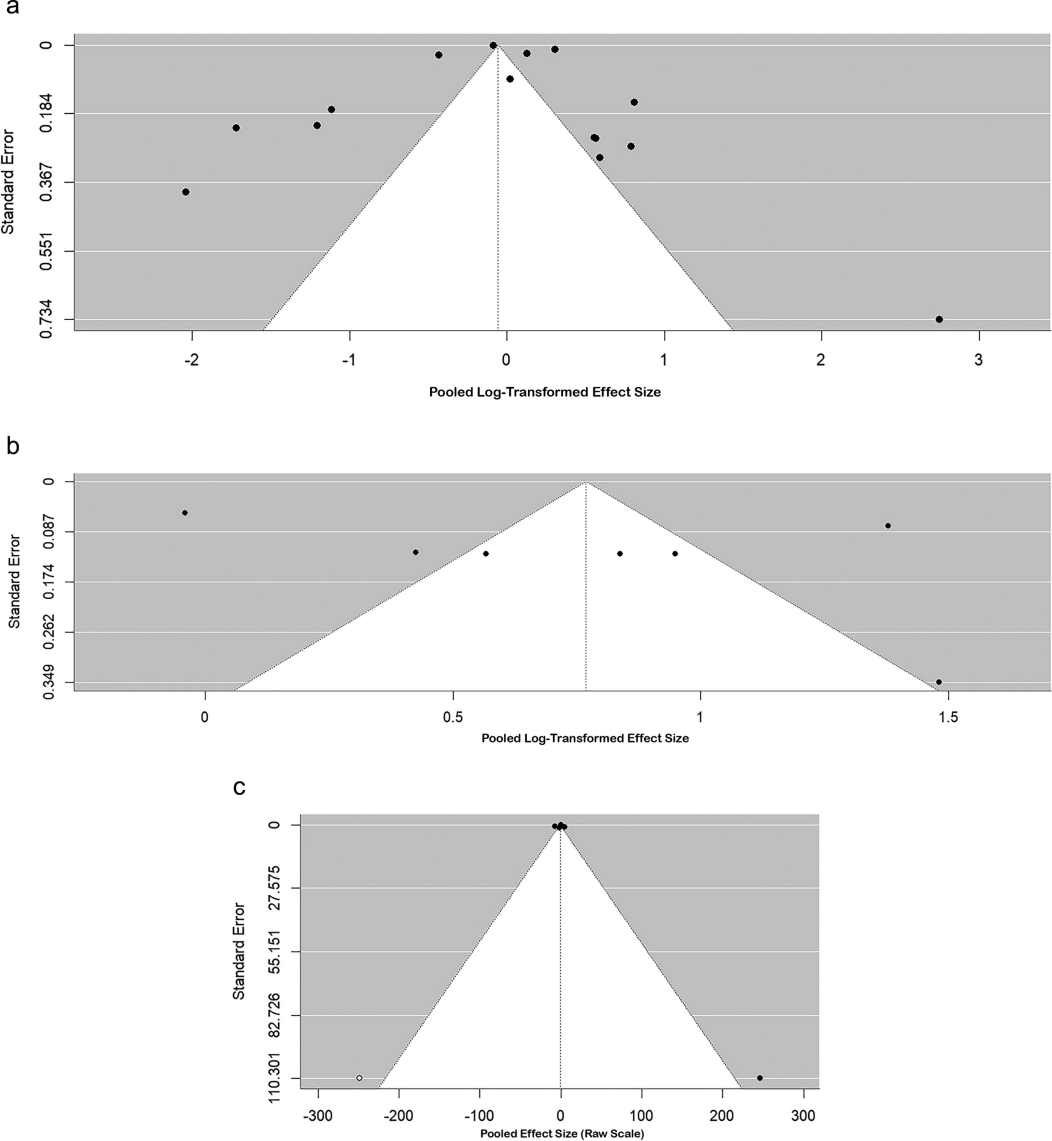
(a) Funnel plot to assess publication bias for clinical outcomes studies; no statistical indication of asymmetry in results. (b) Funnel plot to assess publication bias in immunogenicity studies; no funnel plot asymmetry was detected to be significant. (c) Funnel plot illustrating potential publication bias or small‐study effects in psychosocial outcome studies.

In group A, tests of regression for funnel plot asymmetry provided no discernible evidence of publication bias (*t* = 0.62, *p* = 0.55). Symmetry of the funnel plot implies that the presence of small‐study effects or disappearance of negative studies is unlikely to occur (Figure 3a). The limit estimate as SE near zero indicated minimal adverse effect, but this was not significant enough to lead to asymmetry or bias in reported effects. In group B, the asymmetry regression test of the funnel plot was not significant in offering any evidence of publication bias (*t* = 1.19, *p* = 0.29) (Figure 3b). The bias estimate was also near zero (−0.0383) and with a large CI including zero (−1.40 to 1.32), indicating uncertainty but no missing data or systematic small‐study effect. All of these results together imply that it is unlikely that the combined estimate is distorted by selective publication. In group C, the asymmetry test in the funnel plot indicated statistically significant asymmetry (*z* = 2.20, *p* = 0.028), which could be because of small‐study effects or publication bias (Figure 3c). The −3.24 estimate of bias implied that the smaller studies had more extreme adverse effects. But the estimate's CI was broad and spanned zero (−8.51 to 2.03) and reflects doubt regarding the direction and magnitude of bias. Test statistics of asymmetry in funnel plots and estimates of publication bias for the COVID‐19 outcome groups, including funnel plot asymmetry test statistics and bias estimates, are presented in Table 3. None of the test results indicate statistical publication bias for clinical and immunogenicity outcomes, but reveal significant asymmetry pointing towards potential bias for psychosocial outcomes.

**TABLE 3:** Publication bias assessment for COVID‐19 outcome groups.

Outcome group	Funnel plot asymmetry test statistic	Degrees of freedom (df)	*p*‐value	Bias estimate (b)	95% confidence interval for bias	Interpretation
Clinical outcomes (A)	0.62 (t)	13	0.55	−0.0833	−0.1114 to −0.0553	No statistical evidence of funnel plot asymmetry
Immunogenicity outcomes (B)	1.19 (t)	5	0.29	−0.0383	−1.40 to 1.32	No strong evidence of publication bias or small‐study effects
Psychosocial outcomes (C)	2.20 (z)	4	0.028	−3.24	−8.51 to 2.03	Significant asymmetry; possible publication bias or small‐study effects

### 3.8 Sensitivity Analysis

Within group A, sensitivity analyses examined the influence in sequence of removing individual studies. No study excluded had a significant impact on the pooled effect size, heterogeneity estimates, or significance statistics (see Data S4). Tau^2^ and *I* ^2^ were extremely high across iterations, indicating extreme heterogeneity's resilience and null pooled results' stability irrespective of excluding studies. Within group B, sensitivity analyses entailed the sequential exclusion of all studies and re‐calculation of pooled effects. All leave‐one‐out estimates were significant, a testament to the strength of the immunogenicity effect observed. Leaving out López‐Macías et al. [11] yielded the largest pooled effect size (0.91), and leaving out Berthaud et al. [12] yielded the smallest (0.65), but in both instances, CIs ruled out null effects. Heterogeneity continued to be extremely high (*I* ^2^ > 90%) in all sensitivity analyses, with ongoing variability persisting irrespective of excluding any individual study. In the C group, sensitivity analyses revealed strong non‐significant pooled effects regardless of which study was left out. The estimates covered tightly around the overall effect, and heterogeneity was high (*I* ^2^ always > 85%). Even though estimates of *τ* ^2^ differed (between 8.48 and 32.77), none of the studies influenced the results of the meta‐analysis much, demonstrating the null finding's resistance to heterogeneity.

### 3.9 Leave‐One‐Out Analysis

**FIGURE 4:**
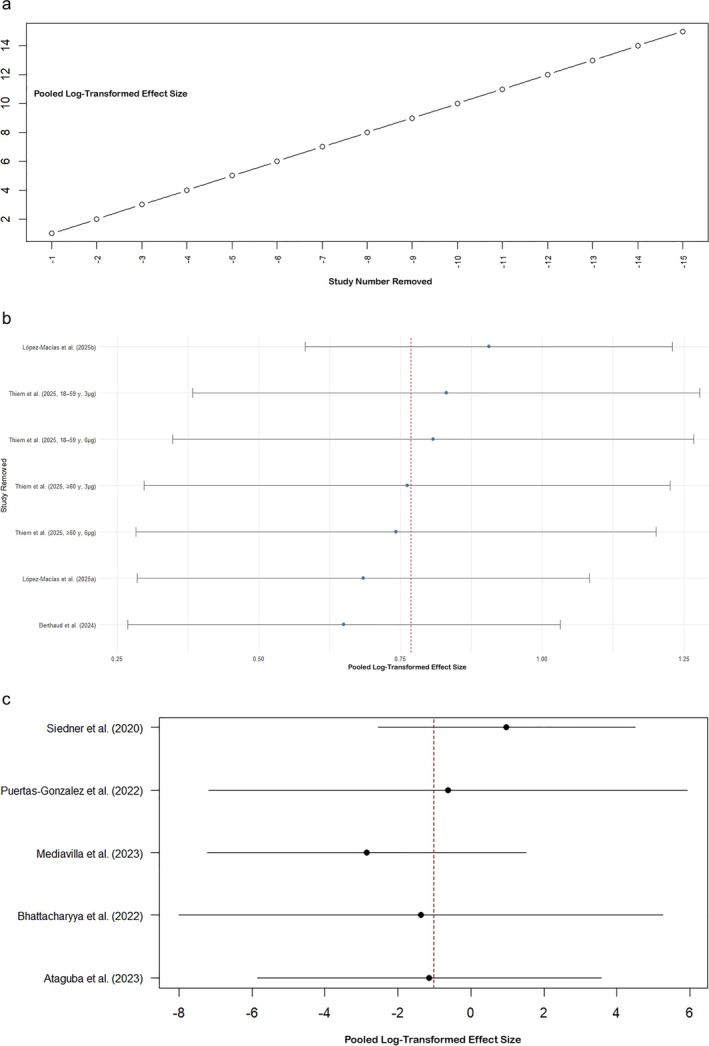
(a) Leave‐one‐out sensitivity analysis to assess robustness of pooled clinical outcomes after removing each study in turn. (b) Leave‐one‐out sensitivity analysis demonstrating robustness of immunogenicity effect estimates when removing individual studies. (c) Leave‐one‐out sensitivity analysis demonstrating the impact of individual psychosocial studies on overall meta‐analytic effect.

In Group A (clinical outcome), leave‐one‐out analysis confirmed that leaving out any single study rendered the pooled effect estimates statistically not significant but of similar size, implying that no single study disproportionately influenced the overall results. For example, the pooled estimates omitting each study were about the combined estimate of −0.0551 with SEs very close to 0.27. The leave‐one‐out sensitivity analysis (Figure 4a) demonstrated the robustness of the overall clinical outcome effect to the exclusion of individual studies. Heterogeneity according to Cochran's Q and *I* ^2^ continued to be very high (about 99.9%), further confirming that individual studies did not disproportionately account for the high heterogeneity observed. Similarly, in Group B (immunogenicity outcomes), sensitivity analysis by leave‐one‐out exclusion confirmed the robustness of pooled effects, with all resulting effect estimates being statistically significant. As shown in Figure 4b, the leave‐one‐out analysis indicates consistent immunogenicity effects regardless of study exclusion. The individual impact of the studies excluded varied a little, having an estimate of around 0.65–0.91, but heterogeneity was high (*I* ^2^ > 90%) in each exclusion and captured similar variation between studies irrespective of exclusion. Leave‐one‐out sensitivity tests within Group C (psychosocial and health service outcomes) revealed pooled effect estimates and measures of heterogeneity altered little following the removal of each of the five studies individually. The leave‐one‐out plot (Figure 4c) highlights the influence of particular studies on the pooled psychosocial outcome effect. The estimates of effect pooled were still not significant, and heterogeneity was still very high (*I* ^2^ > 90%). This is an indication that none of the studies had a substantial impact in determining the results of the meta‐analysis. Generally, quantitative leave‐one‐out sensitivity analyses affirm that the estimates of meta‐analyses are robust and not affected by outliers or influential studies individually, but there is still high study heterogeneity in all groups.

## 4 Discussion

This meta‐analysis combined evidence from three different sets of studies that had investigated the effects of COVID‐19 on clinical, immunogenicity, and health service and psychosocial outcomes. The strategy of grouping was methodologically sound in tackling heterogeneity and diversity between studies that covered as broad a field as here. Although there are still problems in combining diverse data, the findings from the meta‐analyses have valuable implications and pave the way for future studies.

- Clinical outcomes (Group A)

The 15 studies measuring clinical outcomes like mortality, infection rates, hospitalization, adherence, and risks related to equity did not show any statistically significant overall effect. The pooled effect ratio of 0.95 with a very wide CI (0.55–1.62) and high between‐study heterogeneity (*I* ^2^ > 99%) reflects high heterogeneity and no consistent evidence of benefit or harm. These results are consistent with many previous meta‐analyses within the field of COVID‐19 clinical effects, which yield heterogeneous and generally inconclusive outcomes, due to differences in study design, population, and outcome measurement methods [13, 14, 15]. The very high level of heterogeneity seen is characteristic in meta‐analyses of clinical outcomes of COVID‐19 [16]. It highlights the challenges of cross‐setting and cross‐population translation of findings in diverse healthcare settings and populations when variables like healthcare access, viral strains, and public health interventions vary considerably. The absence of a significant pooled effect might also be an expression of the changing nature of clinical management throughout the pandemic, along with differential reporting bias. Notably, this study's absence of publication bias, evidenced by asymmetry tests, suggests the neutrality of pooled findings rather than systematic skewing. In spite of the null result, subgroup analyses according to sample size revealed that heterogeneity could be partially accounted for by study size, suggesting possible small‐study effects reported in earlier studies [17]. Nevertheless, borderline significance in certain subgroups calls for cautious interpretation. Such subtleties underscore the necessity of future large‐scale, methodologically sound clinical trials aligning outcome definitions to minimize heterogeneity and enhance elucidation of clinical effects [18, 19].

ii Immunogenicity outcomes (Group B)

Pooled analysis of four trials' immunogenicity results presented a robust positive effect, a pooled estimate of 0.77 on the log scale with corresponding narrow CIs. This finding is consistent with recent findings emphasizing robust immunogenic responses to COVID‐19 vaccines regardless of population immunological status heterogeneity and vaccine type [20, 21]. Yet, the very high heterogeneity (*I* ^2^ ~96%) reflects genuine variation in effect sizes that may be accounted for by moderators such as vaccine type, dose regimen, populations examined (age groups, co‐morbidities), and chosen immunologic endpoints (neutralizing antibodies vs. seroconversion rates vs. geometric mean titers). Other such meta‐analyses have also demonstrated the same levels of heterogeneity, describing the effect of these moderators on immunogenicity outcomes [22]. Moderator analysis here identified sample size as the principal explanatory variable with more than 80% heterogeneity accounted for. Small studies reported stronger positive effects, and larger studies demonstrated blunted immunogenicity response, consistent with other reviews in which small‐study bias or study population heterogeneity have affected findings [23]. The consistency across sensitivity analyses of the positive pooled effect enhances the confidence of the conclusion that vaccination is associated with important immunogenic benefit, though magnitude and duration may differ by group and by vaccine characteristics. Asymmetry tests for publication bias were insignificant, providing additional assurance that the meta‐analytic conclusion is not a function of selective reporting. These findings underlie continued calls for the utilization of vaccination strategies as backbone interventions during the pandemic and guide future studies into maximizing dosing regimens and booster policy.

iii Psychosocial and health service outcomes (Group C)

This is in contrast to the results of immunogenicity. The five psychosocial factor and health service outcome studies that had been studied yielded extremely heterogeneous and non‐significant combined effects (combined estimate −1.03; *p* = 0.67). This extremely high study variance is a testament to the multifactorial and context‐specific character of psychosocial outcomes, with which a great variety of societal, economic, and cultural factors intervene [24]. The funnel plot asymmetry of large size indicates possible publication bias or small‐study effects, and possibly overrepresentation of studies with greater psychosocial effects. Similar problems have been observed in psychological and health service outcomes meta‐analyses because heterogeneity and bias complicate the drawing of certain conclusions [25]. The moderator analyses indicated sample size explained considerable variance in results and medium‐sized studies showed some positive effects, whereas larger studies showed negative effects. This variation may be a reflection of methodological diversity and diverse population structures, underscoring the need for standardization in psychosocial research methods in the design of pandemic impact evaluations. These findings underscore the necessity of filling evident gaps and the complexity of measuring psychosocial pandemics' impacts, underscoring the imperative need for well‐powered, longitudinal studies that integrate qualitative and quantitative methods. Understanding of the psychosocial consequences is important for informing mental health policy and service provision in ongoing and future public health crises [26].

## 5 Strengths and Limitations

This meta‐analysis was conducted with a systematic search focusing primarily on PubMed, which provided access to an extensive body of peer‐reviewed high‐quality biomedical literature. The preference for assigning the highest priority to PubMed was chosen with the aim of having a solid evidence base because PubMed is a properly maintained and very frequently used database of medical science [27]. This approach provided study screening and selection transparency, increasing the validity of results without making the scope overwhelming. Categorizing results into clinical, immunogenicity, and psychosocial/health service outcomes permitted more focused and specific analyses. This stratification prevented the possibility of combining heterogeneous endpoints with potentially confounded findings, constructing more interpretable domain‐specific results. Further, strict heterogeneity reporting, publication bias, sensitivity, and moderator analyses enabled transparency as well as methodological consistency. Nonetheless, focusing on PubMed studies also meant a relatively smaller sample size, particularly in the immunogenicity and psychosocial areas, thus constraining the scope for subgroup and moderator analyses. While this could be considered a drawback, this is an implicit compromise in the direction of streamlining data relevance and quality instead of quantity, reducing potential noise introduced by poorly optimized reports. This drawback informs the meta‐analysis strategy of filtering studies based on having at least some minimum threshold of methodological quality. The majority of analyses were highly heterogeneous, a common limitation in meta‐analysis of COVID‐19 studies due to the rapidly changing nature of the pandemic, heterogeneous study population, diverse interventions, and differential outcome measurements [28]. While heterogeneity limits the generalizability of summary estimates, it is embraced as a natural attribute rather than a methodological flaw in this review. Extensive moderator and sensitivity analyses were included in this meta‐analysis to aid in the determination of variability determinants and enhance the results' interpretability in spite of heterogeneity in the studies included. The overall approach of the selection and outcome grouping strategy, along with wide‐ranging heterogeneity testing and robustness testing, raises the level of confidence in conclusions while acknowledging in good faith the complexity and limitations in COVID‐19 evidence synthesis currently. Future meta‐analytic investigations can build on this foundation incrementally by adding on routine protocols and larger sets of higher quality evidence.

## 6 Conclusions

This meta‐analysis provides an overall quantification of the impact of COVID‐19 in clinical, immunologic, and psychosocial domains, with an expression of robust immunogenic benefit with vaccines, but a lack of systematic clinical outcome effects and complex psychosocial effects characterized by high heterogeneity. These findings confirm and extend those of earlier syntheses, which comment on the differential effects of the pandemic and the need for ongoing research to further advance understanding and to guide evidence‐based interventions.

## Author Contributions

The author Rulin Wang (RW) and Muhammad Ahsan Naeem (MAN) conceived the study and designed the protocol. Both RW and MAN collected and analyzed the data. RW drafted the initial manuscript; MAN contributed to data interpretation and manuscript revisions.

## Ethics Statement

The authors have nothing to report.

## Conflicts of Interest

The authors declare no conflicts of interest.

## Supporting information


**Data S1:** Supporting information.

**Data S2:** Supporting information.

**Data S3:** Supporting information.

**Data S4:** Supporting information.

## Data Availability

The datasets supporting the conclusions of this study are available on reasonable request to the corresponding author. Data will be shared in line with applicable regulations and policies.
