# Supplementary material for: COVID‐19 Pandemic: A Comprehensive Meta‐Review of Global Impacts, Responses, and Future Preparedness
Source: Clin Respir J. 2025 Nov 21;19(11):e70134. doi: 10.1111/crj.70134 (PMC12635497; doi:10.1111/crj.70134)
Supplement: Supplementary file 1 — Data S1: Supporting information. [file CRJ-19-e70134-s004.docx]

1. **Query**

(((COVID-19 OR SARS-CoV-2 OR Coronavirus AND impact OR effect OR consequence OR burden) AND (response OR intervention OR policy OR public health OR mitigation)) AND (preparedness OR resilience OR future readiness OR response planning)) AND (disparities OR inequities OR vulnerable populations OR low-income countries OR Global South)

1. **PRISMA**
2. **Identification**

- **Total Records (PUBMED, SCOPUS, WOS) = 12445**
- **Duplicate Records (PUBMED, SCOPUS, WOS) = 1468**
- **Total Records Without Duplicates (PUBMED, SCOPUS, WOS) = 10977**
- **Total Records from 2019 to 2025 (PUBMED, SCOPUS, WOS) = 8307**
- **Total Records in English Only (PUBMED, SCOPUS, WOS) = 8077**
- **Records in Other Languages (PUBMED, SCOPUS, WOS) = 230**

We restricted screening to PubMed records because it provides the most comprehensive coverage of biomedical and public health studies, and nearly all eligible RCTs and observational studies were indexed there, while Scopus and WOS contributed largely overlapping or non-eligible records. This ensured feasibility without compromising methodological rigor.

- **Total Records including Quantitative and Qualitative (PUBMED) = 5422**
- **Total Records other than RCT or Observational (PUBMED) = 5275**
- **Total Records including RCT or Observational (PUBMED) = 147**

1. **Screening**

**Total records with full text available (PUBMED) = 113**

**ABSTRACT SCREENING**

**Studies not relevant to COVID-19 = 72+2= 74** (Abera et al., 2022; Al Imam et al., 2023; Amudhan et al., 2021; Bajahzer et al., 2023; Bakkabulindi et al., 2023; Barragan et al., 2022; Betancourt et al., 2020; Büyüköksüz, 2025; Charles et al., 2022; Clarke et al., 2025; Coley et al., 2022; Cousin et al., 2024; Daftary et al., 2021; Dagnew et al., 2025; Donald et al., 2019; Draper et al., 2023; Feng et al., 2025; Furukawa et al., 2025; Gelaw et al., 2024; Goldstein et al., 2025; Gray et al., 2024; Guglielmetti et al., 2025; Gurara et al., 2023; Harrison et al., 2019; Hekker et al., 2025; Henshall et al., 2023; Hill et al., 2020; Hu et al., 2025; Kamke et al., 2019; Kaptchuk et al., 2025; Kurtz et al., 2019; LaMontagne et al., 2025; Lei & Beach, 2020; Li et al., 2022; Liew et al., 2023; Lim et al., 2021; Liu et al., 2025; Llibre-Guerra et al., 2025; Lucas et al., 2021; Lund et al., 2020; Mansfield et al., 2024; Marchewczyk et al., 2025; Maulik et al., 2024; Merrill et al., 2024; Michaud et al., 2024; Motzer et al., 2023; Parker et al., 2022; Patch et al., 2025; Patil et al., 2020; Poolman et al., 2020; Porter et al., 2019; Powles et al., 2022; Pyatak et al., 2023; Ranatunga & Jayaratne, 2020; Rotheram-Borus et al., 2019; Schierhout et al., 2021; Sibanda et al., 2021; Singh et al., 2021; Snowsill et al., 2022; Sol et al., 2024; Sorsdahl et al., 2021; Stevens et al., 2019; Tay et al., 2020; Travers et al., 2022; Valentine et al., 2023; van Heerden et al., 2023; Vargas et al., 2019; Vickery et al., 2021; Wilson et al., 2021; Yoseph et al., 2024; Zeigler et al., 2024; Zeleke et al., 2024; McGuire et al., 2020; Poolman et al., 2025)

**Studies other than RCT or Observational = 3** (Chandiwana et al., 2023; Jibril et al., 2024; Nowicka et al., 2022)

**Studies without statistical data = 1** (Singleton et al., 2023)

**Studies left after abstract screening = 35** (Global Health Research Group on Children’s Non-Communicable Diseases Collaborative, 2022; Aggarwal et al., 2024; Ambrose et al., 2023; Ataguba et al., 2023; Aubert et al., 2021; Berthaud et al., 2024; Bhattacharyya et al., 2022; Bradbury et al., 2022; Bravo et al., 2022; Costa Clemens et al., 2024; Gonçalves et al., 2022; Heath et al., 2023; "Impact of the COVID-19 pandemic on patients with paediatric cancer in low-income, middle-income and high-income countries: a multicentre, international, observational cohort study," 2022; Jennings et al., 2024; López-Macías, Torres, Armenta-Copca, Wacher, Castro-Castrezana, et al., 2025; López-Macías, Torres, Armenta-Copca, Wacher, Galindo-Fraga, et al., 2025; Marbán-Castro et al., 2024; Mayland et al., 2021; Mazingi et al., 2023; Mediavilla et al., 2023; Mnguni et al., 2023; Nice et al., 2025; Nikolaeva & Versnel, 2022; Puertas-Gonzalez et al., 2022; Ramasamy et al., 2021; Reyes et al., 2023; Schöbi et al., 2024; Shinde et al., 2024; Siedner et al., 2020; Sisti et al., 2021; Sokhi et al., 2021; Thiem et al., 2025; Voysey et al., 2021; Wachtler et al., 2024; Williams et al., 2024; Zhang et al., 2022)

**FULL TEXT SCREENING**

**Total studies included for full text screening = 35** (Global Health Research Group on Children’s Non-Communicable Diseases Collaborative, 2022; Aggarwal et al., 2024; Ambrose et al., 2023; Ataguba et al., 2023; Aubert et al., 2021; Berthaud et al., 2024; Bhattacharyya et al., 2022; Bradbury et al., 2022; Bravo et al., 2022; Costa Clemens et al., 2024; Gonçalves et al., 2022; Heath et al., 2023; "Impact of the COVID-19 pandemic on patients with paediatric cancer in low-income, middle-income and high-income countries: a multicentre, international, observational cohort study," 2022; Jennings et al., 2024; López-Macías, Torres, Armenta-Copca, Wacher, Castro-Castrezana, et al., 2025; López-Macías, Torres, Armenta-Copca, Wacher, Galindo-Fraga, et al., 2025; Marbán-Castro et al., 2024; Mayland et al., 2021; Mazingi et al., 2023; Mediavilla et al., 2023; Mnguni et al., 2023; Nice et al., 2025; Nikolaeva & Versnel, 2022; Puertas-Gonzalez et al., 2022; Ramasamy et al., 2021; Reyes et al., 2023; Schöbi et al., 2024; Shinde et al., 2024; Siedner et al., 2020; Sisti et al., 2021; Sokhi et al., 2021; Thiem et al., 2025; Voysey et al., 2021; Wachtler et al., 2024; Williams et al., 2024; Zhang et al., 2022)

**Studies excluded during full text screening = 3** (Costa Clemens et al., 2024; Schöbi et al., 2024; Zhang et al., 2022)

- **Studies excluded due to limited data = 2** (Costa Clemens et al., 2024; Zhang et al., 2022)
- **Study excluded due to Ancillary methodological analysis, not primary outcome data = 1** (Schöbi et al., 2024)

**Total studies left after full text screening = 32** (Global Health Research Group on Children’s Non-Communicable Diseases Collaborative, 2022; Aggarwal et al., 2024; Ambrose et al., 2023; Ataguba et al., 2023; Aubert et al., 2021; Berthaud et al., 2024; Bhattacharyya et al., 2022; Bradbury et al., 2022; Bravo et al., 2022; Gonçalves et al., 2022; Heath et al., 2023; "Impact of the COVID-19 pandemic on patients with paediatric cancer in low-income, middle-income and high-income countries: a multicentre, international, observational cohort study," 2022; Jennings et al., 2024; López-Macías, Torres, Armenta-Copca, Wacher, Castro-Castrezana, et al., 2025; López-Macías, Torres, Armenta-Copca, Wacher, Galindo-Fraga, et al., 2025; Marbán-Castro et al., 2024; Mayland et al., 2021; Mazingi et al., 2023; Mediavilla et al., 2023; Mnguni et al., 2023; Nice et al., 2025; Nikolaeva & Versnel, 2022; Puertas-Gonzalez et al., 2022; Ramasamy et al., 2021; Reyes et al., 2023; Shinde et al., 2024; Siedner et al., 2020; Sisti et al., 2021; Sokhi et al., 2021; Thiem et al., 2025; Voysey et al., 2021; Wachtler et al., 2024; Williams et al., 2024)

**Studies excluded during data extraction due to insufficient data= 8** (Aubert et al., 2021; Marbán-Castro et al., 2024; Mazingi et al., 2023; Mnguni et al., 2023; Nikolaeva & Versnel, 2022; Ramasamy et al., 2021; Shinde et al., 2024; Sokhi et al., 2021)

**Total studies included for quantitative synthesis (meta-analysis) = 24** (Global Health Research Group on Children’s Non-Communicable Diseases Collaborative, 2022; Aggarwal et al., 2024; Ambrose et al., 2023; Ataguba et al., 2023; Berthaud et al., 2024; Bhattacharyya et al., 2022; Bradbury et al., 2022; Bravo et al., 2022; Gonçalves et al., 2022; Heath et al., 2023; "Impact of the COVID-19 pandemic on patients with paediatric cancer in low-income, middle-income and high-income countries: a multicentre, international, observational cohort study," 2022; Jennings et al., 2024; López-Macías, Torres, Armenta-Copca, Wacher, Castro-Castrezana, et al., 2025; López-Macías, Torres, Armenta-Copca, Wacher, Galindo-Fraga, et al., 2025; Mayland et al., 2021; Mediavilla et al., 2023; Nice et al., 2025; Puertas-Gonzalez et al., 2022; Reyes et al., 2023; Siedner et al., 2020; Sisti et al., 2021; Thiem et al., 2025; Voysey et al., 2021; Wachtler et al., 2024; Williams et al., 2024)

**Inclusion and Exclusion Criteria**

| **Category** | **Criteria** |
| --- | --- |
| **Inclusion Criteria** | • **Study design:** Only primary research (RCTs or observational studies: cohort, case-control, cross-sectional, longitudinal) with clear methodology. • **Population:** Human populations with defined sample size and characteristics, from any global region (including Global South). • **Focus:** Must address at least one of the following in the context of COVID-19: – Impacts (health, economic, social, policy) – Responses (intervention, mitigation, public health, policy) – Preparedness (resilience, readiness, future planning) – Equity (disparities, inequities, vulnerable populations) • **Data:** Must report sufficient quantitative/statistical data extractable for meta-analysis (e.g., prevalence, odds ratios, risk ratios, hazard ratios, regression, effect sizes, CIs, p-values, standard errors). • **Language:** Full text available in English. • **Timeframe:** Published from December 2019 onwards. • **Relevance:** Must be directly related to COVID-19 (SARS-CoV-2 pandemic). |
| **Exclusion Criteria** | • Reviews, systematic reviews, scoping reviews, meta-analyses, protocols, commentaries, editorials, theses, dissertations, letters. • Case reports or case series with fewer than 10 participants. • Qualitative-only studies (no quantitative/statistical data). • Laboratory, in vitro, or in silico studies (non-human or simulation only). • Studies without extractable statistical data. • Non-English or inaccessible full-text. • Studies unrelated to COVID-19 (e.g., other diseases or pre-pandemic only data). |

**References for 113 studies**

Abera, G. B., Abebe, S. M., & Worku, A. G. (2022). Impact of health education intervention on demand of women for cervical cancer screening: a cluster-randomized controlled trial. *Trials*, *23*(1), 834. <https://doi.org/10.1186/s13063-022-06765-0>

Aggarwal, N. R., Nordwall, J., Braun, D. L., Chung, L., Coslet, J., Der, T., Eriobu, N., Ginde, A. A., Hayanga, A. J., Highbarger, H., Holodniy, M., Horcajada, J. P., Jain, M. K., Kim, K., Laverdure, S., Lundgren, J., Natarajan, V., Nguyen, H. H., Pett, S. L.,…Reilly, C. (2024). Viral and Host Factors Are Associated With Mortality in Hospitalized Patients With COVID-19. *Clin Infect Dis*, *78*(6), 1490-1503. <https://doi.org/10.1093/cid/ciad780>

Al Imam, M. H., Jahan, I., Das, M. C., Bashar, S. M. K., Khan, A., Muhit, M., Power, R., Akbar, D., Badawi, N., & Khandaker, G. (2023). SUpporting People in extreme POverty with Rehabilitation and Therapy (SUPPORT CP): A trial among families of children with cerebral palsy in Bangladesh. *Dev Med Child Neurol*, *65*(6), 773-782. <https://doi.org/10.1111/dmcn.15445>

Ambrose, N., Amin, A., Anderson, B., Bertagnolli, M., Campion, F., Chow, D., Danan, R., D'Arinzo, L., Drews, A., Erlandson, K., Fitzgerald, K., Gaspar, F., Gong, C., Hanna, G., Hawley, H., Jones, S., Lopansri, B., Mullen, T., Musser, J.,…Yttri, J. (2023). The Influence of Social Determinants on Receiving Outpatient Treatment with Monoclonal Antibodies, Disease Risk, and Effectiveness for COVID-19. *J Gen Intern Med*, *38*(16), 3472-3481. <https://doi.org/10.1007/s11606-023-08324-y>

Amudhan, S., Jangam, K., Mani, K., Murugappan, N. P., Sharma, E., Mahapatra, P., Burma, A. D., Tiwari, H. K., Ashok, A., Vaggar, S., & Rao, G. N. (2021). Project SUMS (scaling up of mental health in schools): design and methods for a pragmatic, cluster randomised waitlist-controlled trial on integrated school mental health intervention for adolescents. *BMC Public Health*, *21*(1), 2034. <https://doi.org/10.1186/s12889-021-12086-9>

Ataguba, J. E., Birungi, C., Cunial, S., & Kavanagh, M. (2023). Income inequality and pandemics: insights from HIV/AIDS and COVID-19-a multicountry observational study. *BMJ Glob Health*, *8*(9). <https://doi.org/10.1136/bmjgh-2023-013703>

Aubert, O., Yoo, D., Zielinski, D., Cozzi, E., Cardillo, M., Dürr, M., Domínguez-Gil, B., Coll, E., Da Silva, M. I., Sallinen, V., Lemström, K., Midtvedt, K., Ulloa, C., Immer, F., Weissenbacher, A., Vallant, N., Basic-Jukic, N., Tanabe, K., Papatheodoridis, G.,…Loupy, A. (2021). COVID-19 pandemic and worldwide organ transplantation: a population-based study. *Lancet Public Health*, *6*(10), e709-e719. <https://doi.org/10.1016/s2468-2667(21)00200-0>

Bajahzer, M. F., Rosqvist, F., Fridén, M., Iggman, D., Pingel, R., Marklund, M., & Risérus, U. (2023). Contrasting Carbohydrate Quantity and Quality and the Effects on Plasma Saturated and Monounsaturated Fatty Acids in Healthy Adults: A Randomized Controlled Trial. *J Nutr*, *153*(3), 683-690. <https://doi.org/10.1016/j.tjnut.2023.01.005>

Bakkabulindi, P., Ampeire, I., Ayebale, L., Mubiri, P., Feletto, M., & Muhumuza, S. (2023). Engagement of community health workers to improve immunization coverage through addressing inequities and enhancing data quality and use is a feasible and effective approach: An implementation study in Uganda. *PLoS One*, *18*(10), e0292053. <https://doi.org/10.1371/journal.pone.0292053>

Barragan, M., Luna, V., Hammons, A. J., Olvera, N., Greder, K., Drumond Andrade, F. C., Fiese, B., Wiley, A., Teran-Garcia, M., & The Abriendo Caminos Research, T. (2022). Reducing Obesogenic Eating Behaviors in Hispanic Children through a Family-Based, Culturally-Tailored RCT: Abriendo Caminos. *Int J Environ Res Public Health*, *19*(4). <https://doi.org/10.3390/ijerph19041917>

Berthaud, V., Creech, C. B., Rostad, C. A., Carr, Q., de Leon, L., Dietrich, M., Gupta, A., Javita, D., Nachman, S., Pinninti, S., Rathore, M., Rodriguez, C. A., Luzuriaga, K., Towner, W., Yeakey, A., Brown, M., Zhao, X., Deng, W., Xu, W.,…Schnyder Ghamloush, S. (2024). Safety and Immunogenicity of an mRNA-1273 Booster in Children. *Clin Infect Dis*, *79*(6), 1524-1532. <https://doi.org/10.1093/cid/ciae420>

Betancourt, T. S., Berent, J. M., Freeman, J., Frounfelker, R. L., Brennan, R. T., Abdi, S., Maalim, A., Abdi, A., Mishra, T., Gautam, B., Creswell, J. W., & Beardslee, W. R. (2020). Family-Based Mental Health Promotion for Somali Bantu and Bhutanese Refugees: Feasibility and Acceptability Trial. *J Adolesc Health*, *66*(3), 336-344. <https://doi.org/10.1016/j.jadohealth.2019.08.023>

Bhattacharyya, R., Burman, A., Singh, K., Banerjee, S., Maity, S., Auddy, A., Rout, S. K., Lahoti, S., Panda, R., & Baladandayuthapani, V. (2022). Role of multiresolution vulnerability indices in COVID-19 spread in India: a Bayesian model-based analysis. *BMJ Open*, *12*(11), e056292. <https://doi.org/10.1136/bmjopen-2021-056292>

Bradbury, C. A., Lawler, P. R., Stanworth, S. J., McVerry, B. J., McQuilten, Z., Higgins, A. M., Mouncey, P. R., Al-Beidh, F., Rowan, K. M., Berry, L. R., Lorenzi, E., Zarychanski, R., Arabi, Y. M., Annane, D., Beane, A., van Bentum-Puijk, W., Bhimani, Z., Bihari, S., Bonten, M. J. M.,…Gordon, A. C. (2022). Effect of Antiplatelet Therapy on Survival and Organ Support-Free Days in Critically Ill Patients With COVID-19: A Randomized Clinical Trial. *Jama*, *327*(13), 1247-1259. <https://doi.org/10.1001/jama.2022.2910>

Bravo, L., Smolenov, I., Han, H. H., Li, P., Hosain, R., Rockhold, F., Clemens, S. A. C., Roa, C., Jr., Borja-Tabora, C., Quinsaat, A., Lopez, P., López-Medina, E., Brochado, L., Hernández, E. A., Reynales, H., Medina, T., Velasquez, H., Toloza, L. B., Rodriguez, E. J.,…Clemens, R. (2022). Efficacy of the adjuvanted subunit protein COVID-19 vaccine, SCB-2019: a phase 2 and 3 multicentre, double-blind, randomised, placebo-controlled trial. *Lancet*, *399*(10323), 461-472. <https://doi.org/10.1016/s0140-6736(22)00055-1>

Büyüköksüz, E. (2025). Effectiveness of the acceptance and commitment therapy for resilience promotion in a non-clinical sample: A randomized controlled and a pilot study. *BMC Psychol*, *13*(1), 698. <https://doi.org/10.1186/s40359-025-03023-1>

Chandiwana, N., Kruger, C., Richardson, N., Nxumalo, S., Mashilo, N., Dineka, Y., Mudau, N., Johnstone, H., Kim, W., Ju, C., Arbe-Barnes, S., Marrast, A. C., Flynn, J., & Venter, W. D. F. (2023). Community-based management of a five-arm randomised clinical trial in COVID-19 outpatients in South Africa: challenges and opportunities. *Trials*, *24*(1), 635. <https://doi.org/10.1186/s13063-023-07577-6>

Charles, K. J., Howard, G., Villalobos Prats, E., Gruber, J., Alam, S., Alamgir, A. S. M., Baidya, M., Flora, M. S., Haque, F., Hassan, S. M. Q., Islam, S., Lazaro, A., Lwetoijera, D. W., Mahmud, S. G., Mahmud, Z. H., Matwewe, F., Pasa, K., Rahman, M., Reza, A. A. S.,…Campbell-Lendrum, D. (2022). Infrastructure alone cannot ensure resilience to weather events in drinking water supplies. *Sci Total Environ*, *813*, 151876. <https://doi.org/10.1016/j.scitotenv.2021.151876>

Clarke, N. W., Armstrong, A. J., Oya, M., Shore, N., Procopio, G., Daniel Guedes, J., Arslan, C., Mehra, N., Parnis, F., Brown, E., Schlürmann, F., Young Joung, J., Sugimoto, M., Sartor, O., Poehlein, C., McGuinness, D., Degboe, A., & Saad, F. (2025). Efficacy and Safety of Olaparib Plus Abiraterone Versus Placebo Plus Abiraterone in the First-line Treatment of Patients with Asymptomatic/Mildly Symptomatic and Symptomatic Metastatic Castration-resistant Prostate Cancer: Analyses from the Phase 3 PROpel Trial. *Eur Urol Oncol*, *8*(2), 394-406. <https://doi.org/10.1016/j.euo.2024.09.013>

Coley, N., Andre, L., Hoevenaar-Blom, M. P., Ngandu, T., Beishuizen, C., Barbera, M., van Wanrooij, L., Kivipelto, M., Soininen, H., van Gool, W., Brayne, C., Moll van Charante, E., Richard, E., & Andrieu, S. (2022). Factors Predicting Engagement of Older Adults With a Coach-Supported eHealth Intervention Promoting Lifestyle Change and Associations Between Engagement and Changes in Cardiovascular and Dementia Risk: Secondary Analysis of an 18-Month Multinational Randomized Controlled Trial. *J Med Internet Res*, *24*(5), e32006. <https://doi.org/10.2196/32006>

Costa Clemens, S. A., Jepson, B., Bhorat, Q. E., Ahmad, A., Akhund, T., Aley, P. K., Bansal, H., Bibi, S., Kelly, E. J., Khan, M., Lambe, T., Lombaard, J. J., Matthews, S., Pipolo Milan, E., Olsson, U., Ramasamy, M. N., Moura de Oliveira Paiva, M. S., Seegobin, S., Shoemaker, K.,…Green, J. A. (2024). Immunogenicity and safety of beta variant COVID-19 vaccine AZD2816 and AZD1222 (ChAdOx1 nCoV-19) as primary-series vaccination for previously unvaccinated adults in Brazil, South Africa, Poland, and the UK: a randomised, partly double-blinded, phase 2/3 non-inferiority immunobridging study. *Lancet Microbe*, *5*(8), 100863. <https://doi.org/10.1016/s2666-5247(24)00078-8>

Cousin, L., Braithwaite, D., Anton, S., Zhang, Z., Lee, J. H., Leewenburgh, C., & Lyon, D. (2024). A pilot study of a gratitude journaling intervention to enhance spiritual well-being and exercise self-efficacy in Black breast cancer survivors. *BMC Psychiatry*, *24*(1), 931. <https://doi.org/10.1186/s12888-024-06362-2>

Daftary, A., Mondal, S., Zelnick, J., Friedland, G., Seepamore, B., Boodhram, R., Amico, K. R., Padayatchi, N., & O'Donnell, M. R. (2021). Dynamic needs and challenges of people with drug-resistant tuberculosis and HIV in South Africa: a qualitative study. *Lancet Glob Health*, *9*(4), e479-e488. <https://doi.org/10.1016/s2214-109x(20)30548-9>

Dagnew, A. F., Han, L. L., Naidoo, K., Fairlie, L., Innes, J. C., Middelkoop, K., Tameris, M., Wilkinson, R. J., Ananworanich, J., Bower, D., Schlehuber, L., Frahm, N., Cinar, A., Dunne, M., & Schmidt, A. C. (2025). Safety and immunogenicity of investigational tuberculosis vaccine M72/AS01(E-4) in people living with HIV in South Africa: an observer-blinded, randomised, controlled, phase 2 trial. *Lancet HIV*, *12*(8), e546-e555. <https://doi.org/10.1016/s2352-3018(25)00124-9>

Donald, K. A., Wedderburn, C. J., Barnett, W., Nhapi, R. T., Rehman, A. M., Stadler, J. A. M., Hoffman, N., Koen, N., Zar, H. J., & Stein, D. J. (2019). Risk and protective factors for child development: An observational South African birth cohort. *PLoS Med*, *16*(9), e1002920. <https://doi.org/10.1371/journal.pmed.1002920>

Draper, C. E., Motlhatlhedi, M., Mabasa, J., Headman, T., Klingberg, S., Pentecost, M., Lye, S. J., Norris, S. A., & Nyati, L. H. (2023). Navigating relationship dynamics, pregnancy and fatherhood in the Bukhali trial: a qualitative study with men in Soweto, South Africa. *BMC Public Health*, *23*(1), 2204. <https://doi.org/10.1186/s12889-023-17153-x>

Feng, S., Zhang, Y., Khanam, F., Voysey, M., Pitzer, V. E., Qadri, F., Clemens, J. D., Pollard, A. J., & Liu, X. (2025). The validity of test-negative design for assessment of typhoid conjugate vaccine protection: comparison of estimates by different study designs using data from a cluster-randomised controlled trial. *Lancet Glob Health*, *13*(6), e1122-e1131. <https://doi.org/10.1016/s2214-109x(25)00056-7>

Furukawa, T. A., Tajika, A., Toyomoto, R., Sakata, M., Luo, Y., Horikoshi, M., Akechi, T., Kawakami, N., Nakayama, T., Kondo, N., Fukuma, S., Kessler, R. C., Christensen, H., Whitton, A., Nahum-Shani, I., Lutz, W., Cuijpers, P., Wason, J. M. S., & Noma, H. (2025). Cognitive behavioral therapy skills via a smartphone app for subthreshold depression among adults in the community: the RESiLIENT randomized controlled trial. *Nat Med*, *31*(6), 1830-1839. <https://doi.org/10.1038/s41591-025-03639-1>

Gelaw, S. G., Deyessa, N., Kidane, A., Evensen, A., Teka, A., Bokan, B., & Yesuf, S. A. (2024). Effect of postpartum family planning intervention and associated factors during child immunization in Addis Ababa, Ethiopia. *Pan Afr Med J*, *47*, 110. <https://doi.org/10.11604/pamj.2024.47.110.34883>

Global Health Research Group on Children’s Non-Communicable Diseases Collaborative. Impact of the COVID-19 pandemic on patients with paediatric cancer in low-income, middle-income and high-income countries: a multicentre, international, observational cohort study. BMJ open. 2022 Apr;12(4):e054690. <https://doi.org/10.1136/bmjopen-2021-054690>

Goldstein, E., Keita, M., Koomson, C., Tintle, N., Adlam, K., Farah, E., & Koenig, M. D. (2025). A Pilot Randomized Controlled Trial of a Multimodal Wellness Intervention for Perinatal Mental Health. *J Midwifery Womens Health*, *70*(3), 442-451. <https://doi.org/10.1111/jmwh.13754>

Gonçalves, B. P., Hall, M., Jassat, W., Balan, V., Murthy, S., Kartsonaki, C., Semple, M. G., Rojek, A., Baruch, J., Reyes, L. F., Dasgupta, A., Dunning, J., Citarella, B. W., Pritchard, M., Martín-Quiros, A., Sili, U., Baillie, J. K., Aryal, D., Arabi, Y.,…Olliaro, P. L. (2022). An international observational study to assess the impact of the Omicron variant emergence on the clinical epidemiology of COVID-19 in hospitalised patients. *Elife*, *11*. <https://doi.org/10.7554/eLife.80556>

Gray, G. E., Mngadi, K., Lavreys, L., Nijs, S., Gilbert, P. B., Hural, J., Hyrien, O., Juraska, M., Luedtke, A., Mann, P., McElrath, M. J., Odhiambo, J. A., Stieh, D. J., van Duijn, J., Takalani, A. N., Willems, W., Tapley, A., Tomaras, G. D., Van Hoof, J.,…Tomaka, F. (2024). Mosaic HIV-1 vaccine regimen in southern African women (Imbokodo/HVTN 705/HPX2008): a randomised, double-blind, placebo-controlled, phase 2b trial. *Lancet Infect Dis*, *24*(11), 1201-1212. <https://doi.org/10.1016/s1473-3099(24)00358-x>

Guglielmetti, L., Khan, U., Velásquez, G. E., Gouillou, M., Ali, M. H., Amjad, S., Kamal, F., Abubakirov, A., Ardizzoni, E., Baudin, E., Bektassov, S., Berry, C., Bonnet, M., Chavan, V., Coutisson, S., Dakenova, Z., de Jong, B. C., Dinh, L. V., Ferlazzo, G.,…Mitnick, C. D. (2025). Bedaquiline, delamanid, linezolid, and clofazimine for rifampicin-resistant and fluoroquinolone-resistant tuberculosis (endTB-Q): an open-label, multicentre, stratified, non-inferiority, randomised, controlled, phase 3 trial. *Lancet Respir Med*, *13*(9), 809-820. <https://doi.org/10.1016/s2213-2600(25)00194-8>

Gurara, M. K., Draulans, V., Jacquemyn, Y., & Van Geertruyden, J. P. (2023). Evaluation of a community-based intervention package to improve knowledge of obstetric danger signs, birth preparedness, and institutional delivery care utilization in Arba Minch Zuria District, Ethiopia: a cluster-randomized trial. *Reprod Health*, *20*(1), 169. <https://doi.org/10.1186/s12978-023-01713-w>

Harrison, S. E., Li, X., Zhang, J., Zhao, J., & Zhao, G. (2019). A cluster randomized controlled trial to evaluate a resilience-based intervention for caregivers of HIV-affected children in China. *Aids*, *33 Suppl 1*(Suppl 1), S81-s91. <https://doi.org/10.1097/qad.0000000000002181>

Heath, P. T., Galiza, E. P., Baxter, D. N., Boffito, M., Browne, D., Burns, F., Chadwick, D. R., Clark, R., Cosgrove, C. A., Galloway, J., Goodman, A. L., Heer, A., Higham, A., Iyengar, S., Jeanes, C., Kalra, P. A., Kyriakidou, C., Bradley, J. M., Munthali, C.,…Toback, S. (2023). Safety and Efficacy of the NVX-CoV2373 Coronavirus Disease 2019 Vaccine at Completion of the Placebo-Controlled Phase of a Randomized Controlled Trial. *Clin Infect Dis*, *76*(3), 398-407. <https://doi.org/10.1093/cid/ciac803>

Hekker, M. D., Platteel, T. N., Venekamp, R. P., Top, J., Geerlings, S. E., Schultsz, C., de Vos, M. G. J., & van de Wijgert, J. (2025). Urinary tract infections in postmenopausal women revisited (UTIr): a prospective observational cohort study to explore the urobiomes of postmenopausal women with and without recurrent urinary tract infections. *BMC Infect Dis*, *25*(1), 822. <https://doi.org/10.1186/s12879-025-11269-8>

Henshall, C., Davey, Z., Srikesavan, C., Hart, L., Butcher, D., & Cipriani, A. (2023). Implementation of a Web-Based Resilience Enhancement Training for Nurses: Pilot Randomized Controlled Trial. *J Med Internet Res*, *25*, e43771. <https://doi.org/10.2196/43771>

Hill, K. D., Baldwin, H. S., Bichel, D. P., Butts, R. J., Chamberlain, R. C., Ellis, A. M., Graham, E. M., Hickerson, J., Hornik, C. P., Jacobs, J. P., Jacobs, M. L., Jaquiss, R. D., Kannankeril, P. J., O'Brien, S. M., Torok, R., Turek, J. W., & Li, J. S. (2020). Rationale and design of the STeroids to REduce Systemic inflammation after infant heart Surgery (STRESS) trial. *Am Heart J*, *220*, 192-202. <https://doi.org/10.1016/j.ahj.2019.11.016>

Hu, X., Zhang, Q., Sun, T., Yin, Y., Li, H., Yan, M., Tong, Z., Li, M., Teng, Y., Oppermann, C. P., Kanakasetty, G. B., Portugal, M. C., Yang, L., Zhang, W., & Jiang, Z. (2025). Abemaciclib plus non-steroidal aromatase inhibitor or fulvestrant in women with HR+/HER2- advanced breast cancer: Final results of the randomized phase III MONARCH plus trial. *Chin Med J (Engl)*, *138*(12), 1477-1486. <https://doi.org/10.1097/cm9.0000000000003151>

Impact of the COVID-19 pandemic on patients with paediatric cancer in low-income, middle-income and high-income countries: a multicentre, international, observational cohort study. (2022). *BMJ Open*, *12*(4), e054690. <https://doi.org/10.1136/bmjopen-2021-054690>

Jennings, K., Lembani, M., Hesseling, A. C., Mbula, N., Mohr-Holland, E., Mudaly, V., Smith, M., Osman, M., & Meehan, S. A. (2024). A decline in tuberculosis diagnosis, treatment initiation and success during the COVID-19 pandemic, using routine health data in Cape Town, South Africa. *PLoS One*, *19*(9), e0310383. <https://doi.org/10.1371/journal.pone.0310383>

Jibril, H., Zaman, M. A., Saadat, E., Zafar Mahmood, S. B., Awan, S., & Arshad, A. (2024). Influencing perception of COVID-19 illness and vaccines using an educational intervention tool in a lower-middle-income country. *J Pak Med Assoc*, *74*(12), 2171-2174. <https://doi.org/10.47391/jpma.20108>

Kamke, K., Grenen, E., Robinson, C., & El-Toukhy, S. (2019). Dropout and Abstinence Outcomes in a National Text Messaging Smoking Cessation Intervention for Pregnant Women, SmokefreeMOM: Observational Study. *JMIR Mhealth Uhealth*, *7*(10), e14699. <https://doi.org/10.2196/14699>

Kaptchuk, R. P., Kant, A., Shekhawat, S. S., Baishya, J., Sinha, A., Kedar, A., Khanna, S., McFall, A. M., Solomon, S. S., Mehta, S. H., & Lucas, G. M. (2025). Social support and HIV management among people who inject drugs: in-depth interviews in Delhi, India. *Harm Reduct J*, *22*(1), 32. <https://doi.org/10.1186/s12954-025-01185-0>

Kurtz, S. P., Pagano, M. E., Buttram, M. E., & Ungar, M. (2019). Brief interventions for young adults who use drugs: The moderating effects of resilience and trauma. *J Subst Abuse Treat*, *101*, 18-24. <https://doi.org/10.1016/j.jsat.2019.03.009>

LaMontagne, A. D., Lockwood, C., Mackinnon, A., Henry, D., Cox, L., Hall, N. R., & King, T. L. (2025). MATES in Manufacturing: A Cluster RCT Evaluation of a Workplace Suicide Prevention Program. *Am J Ind Med*, *68*(4), 331-343. <https://doi.org/10.1002/ajim.23698>

Lei, M. K., & Beach, S. R. H. (2020). Can We Uncouple Neighborhood Disadvantage and Delinquent Behaviors? An Experimental Test of Family Resilience Guided by the Social Disorganization Theory of Delinquent Behaviors. *Fam Process*, *59*(4), 1801-1817. <https://doi.org/10.1111/famp.12527>

Li, M., Han, Q., Pan, Z., Wang, K., Xie, J., Zheng, B., & Lv, J. (2022). Effectiveness of Multidomain Dormitory Environment and Roommate Intervention for Improving Sleep Quality of Medical College Students: A Cluster Randomised Controlled Trial in China. *Int J Environ Res Public Health*, *19*(22). <https://doi.org/10.3390/ijerph192215337>

Liew, S. L., Schweighofer, N., Cole, J. H., Zavaliangos-Petropulu, A., Tavenner, B. P., Han, L. K. M., Hahn, T., Schmaal, L., Donnelly, M. R., Jeong, J. N., Wang, Z., Abdullah, A., Kim, J. H., Hutton, A., Barisano, G., Borich, M. R., Boyd, L. A., Brodtmann, A., Buetefisch, C. M.,…Thompson, P. M. (2023). Association of Brain Age, Lesion Volume, and Functional Outcome in Patients With Stroke. *Neurology*, *100*(20), e2103-e2113. <https://doi.org/10.1212/wnl.0000000000207219>

Lim, L. L., Lau, E. S. H., Fu, A. W. C., Ray, S., Hung, Y. J., Tan, A. T. B., Chamnan, P., Sheu, W. H. H., Chawla, M. S., Chia, Y. C., Chuang, L. M., Nguyen, D. C., Sosale, A., Saboo, B. D., Phadke, U., Kesavadev, J., Goh, S. Y., Gera, N., Huyen Vu, T. T.,…Chan, J. C. N. (2021). Effects of a Technology-Assisted Integrated Diabetes Care Program on Cardiometabolic Risk Factors Among Patients With Type 2 Diabetes in the Asia-Pacific Region: The JADE Program Randomized Clinical Trial. *JAMA Netw Open*, *4*(4), e217557. <https://doi.org/10.1001/jamanetworkopen.2021.7557>

Liu, Q., Wang, Y., Liu, M., Zhao, Y., & Liu, J. (2025). The influence and lag-effect of temperature and precipitation on the incidence and mortality of tuberculosis, 2000-2021: an observational study. *Front Public Health*, *13*, 1572422. <https://doi.org/10.3389/fpubh.2025.1572422>

Llibre-Guerra, J. J., Llerena, T. Z., Figueroa, Z. M., Almirall-Sánchez, A., Rodríguez-Velasco, A., Santos Martínez, A., Gutiérrez-Herrera, R. F., Rodríguez-Salgado, A. M., & Llibre-Rodríguez, J. J. (2025). Advancing dementia preparedness in Low and Middle Income countries: A randomized trial to improve diagnosis in primary care. *Alzheimers Dement*, *21*(5), e70283. <https://doi.org/10.1002/alz.70283>

López-Macías, C., Torres, M., Armenta-Copca, B., Wacher, N. H., Castro-Castrezana, L., Colli-Domínguez, A. A., Rivera-Hernández, T., Torres-Flores, A., Damián-Hernández, M., Ramírez-Martínez, L., la Rosa, G. P., Rojas-Martínez, O., Suárez-Martínez, A., Peralta-Sánchez, G., Carranza, C., Juárez, E., Zamudio-Meza, H., Carreto-Binaghi, L. E., Viettri, M.,…Lozano-Dubernard, B. (2025). Phase II study on the safety and immunogenicity of single-dose intramuscular or intranasal administration of the AVX/COVID-12 "Patria" recombinant Newcastle disease virus vaccine as a heterologous booster against COVID-19 in Mexico. *Vaccine*, *43*(Pt 2), 126511. <https://doi.org/10.1016/j.vaccine.2024.126511>

López-Macías, C., Torres, M., Armenta-Copca, B., Wacher, N. H., Galindo-Fraga, A., Castro-Castrezana, L., Colli-Domínguez, A. A., Cervantes-Trujano, E., Rucker-Joerg, I. E., Lozano-Patiño, F., Rivera-Alcocer, J. J., Simón-Campos, A., Sánchez-Campos, E. A., Aguirre-Rivero, R., Muñiz-Carvajal, A. J., Del Carpio-Orantes, L., Márquez-Díaz, F., Rivera-Hernández, T., Torres-Flores, A.,…Lozano-Dubernard, B. (2025). Phase 2/3 study evaluating safety, immunogenicity, and noninferiority of single booster dose of AVX/COVID-12 vaccine. *Sci Adv*, *11*(26), eadq2887. <https://doi.org/10.1126/sciadv.adq2887>

Lucas, T., Thompson, H. S., Blessman, J., Dawadi, A., Drolet, C. E., Hirko, K. A., & Penner, L. A. (2021). Effects of culturally targeted message framing on colorectal cancer screening among African Americans. *Health Psychol*, *40*(5), 305-315. <https://doi.org/10.1037/hea0001073>

Lund, C., Schneider, M., Garman, E. C., Davies, T., Munodawafa, M., Honikman, S., Bhana, A., Bass, J., Bolton, P., Dewey, M., Joska, J., Kagee, A., Myer, L., Petersen, I., Prince, M., Stein, D. J., Tabana, H., Thornicroft, G., Tomlinson, M.,…Susser, E. (2020). Task-sharing of psychological treatment for antenatal depression in Khayelitsha, South Africa: Effects on antenatal and postnatal outcomes in an individual randomised controlled trial. *Behav Res Ther*, *130*, 103466. <https://doi.org/10.1016/j.brat.2019.103466>

Mansfield, A. S., Vivien Yin, J., Bradbury, P., Kwiatkowski, D. J., Patel, S., Bazhenova, L. A., Forde, P., Lou, Y., Dizona, P., Villaruz, L. C., Arnold, S. M., Khalil, M., Kindler, H. L., Koczywas, M., Pacheco, J., Rolfo, C., Xia, B., Mikula, E., Chen, L.,…Hassan, R. (2024). Randomized trial of anetumab ravtansine and pembrolizumab compared to pembrolizumab for mesothelioma. *Lung Cancer*, *195*, 107928. <https://doi.org/10.1016/j.lungcan.2024.107928>

Marbán-Castro, E., Getia, V., Alkhazashvili, M., Japaridze, M., Jikia, I., Erkosar, B., Del Rey-Puech, P., Martínez-Pérez, G. Z., Imnadze, P., & Gamkrelidze, A. (2024). Implementing a pilot study of COVID-19 self-testing in high-risk populations and remote locations: results and lessons learnt. *BMC Public Health*, *24*(1), 511.

Marchewczyk, P., Costeira, B., da Silva, F. B., Cavadas, D., Abecasis, N., Limbert, M., & Maciel, J. (2025). Quality of life outcomes in colorectal cancer survivors: insights from an observational study at a tertiary cancer center. *Qual Life Res*, *34*(5), 1501-1514. <https://doi.org/10.1007/s11136-025-03918-x>

Maulik, P. K., Daniel, M., Devarapalli, S., Kallakuri, S., Kaur, A., Ghosh, A., Billot, L., Mukherjee, A., Sagar, R., Kant, S., Chatterjee, S., Essue, B. M., Raman, U., Praveen, D., Thornicroft, G., Saxena, S., Patel, A., & Peiris, D. (2024). Mental Health Care Support in Rural India: A Cluster Randomized Clinical Trial. *JAMA Psychiatry*, *81*(11), 1061-1070. <https://doi.org/10.1001/jamapsychiatry.2024.2305>

Mayland, C. R., Hughes, R., Lane, S., McGlinchey, T., Donnellan, W., Bennett, K., Hanna, J., Rapa, E., Dalton, L., & Mason, S. R. (2021). Are public health measures and individualised care compatible in the face of a pandemic? A national observational study of bereaved relatives' experiences during the COVID-19 pandemic. *Palliat Med*, *35*(8), 1480-1491.

<https://doi.org/10.1177/02692163211019885>

Mazingi, D., Shinondo, P., Ihediwa, G., Ford, K., Ademuyiwa, A., & Lakhoo, K. (2023). The impact of the COVID-19 pandemic on paediatric surgical volumes in Africa: A retrospective observational study. *J Pediatr Surg*, *58*(2), 275-281. <https://doi.org/10.1016/j.jpedsurg.2022.10.047>

McGuire JF, Ricketts EJ, Scahill L, Wilhelm S, Woods DW, Piacentini J, Walkup JT, Peterson AL. Effect of behavior therapy for Tourette's disorder on psychiatric symptoms and functioning in adults. Psychological medicine. 2020 Sep;50(12):2046-56. https://doi.org/10.1017/S0033291719002150

Mediavilla, R., Felez-Nobrega, M., McGreevy, K. R., Monistrol-Mula, A., Bravo-Ortiz, M. F., Bayón, C., Giné-Vázquez, I., Villaescusa, R., Muñoz-Sanjosé, A., Aguilar-Ortiz, S., Figueiredo, N., Nicaise, P., Park, A. L., Petri-Romão, P., Purgato, M., Witteveen, A. B., Underhill, J., Barbui, C., Bryant, R.,…Ayuso-Mateos, J. L. (2023). Effectiveness of a mental health stepped-care programme for healthcare workers with psychological distress in crisis settings: a multicentre randomised controlled trial. *BMJ Ment Health*, *26*(1). <https://doi.org/10.1136/bmjment-2023-300697>

Merrill, S. M., Hogan, C., Bozack, A. K., Cardenas, A., Comer, J. S., Bagner, D. M., Highlander, A., & Parent, J. (2024). Telehealth Parenting Program and Salivary Epigenetic Biomarkers in Preschool Children With Developmental Delay: NIMHD Social Epigenomics Program. *JAMA Netw Open*, *7*(7), e2424815. <https://doi.org/10.1001/jamanetworkopen.2024.24815>

Michaud, T. L., Zagurski, C., Wilson, K. E., Porter, G. C., Johnson, G., & Estabrooks, P. A. (2024). Reach and Weight Loss Among Comparison Group Participants Who Enrolled in the Active Intervention After a Diabetes Prevention Trial. *Prev Chronic Dis*, *21*, E40. <https://doi.org/10.5888/pcd21.230358>

Mnguni, A. T., Schietekat, D., Ebrahim, N., Sonday, N., Boliter, N., Schrueder, N., Gabriels, S., Sigwadhi, L. N., Zemlin, A. E., Chapanduka, Z. C., Ngah, V., Yalew, A., Jalavu, T., Abdullah, I., Tamuzi, J. L., Tembo, Y., Davies, M. A., English, R., & Nyasulu, P. S. (2023). The clinical and epidemiological characteristics of a series of patients living with HIV admitted for COVID-19 in a district hospital. *BMC Infect Dis*, *23*(1), 123. <https://doi.org/10.1186/s12879-023-08004-6>

Motzer, R. J., Russo, P., Grünwald, V., Tomita, Y., Zurawski, B., Parikh, O., Buti, S., Barthélémy, P., Goh, J. C., Ye, D., Lingua, A., Lattouf, J. B., Albigès, L., George, S., Shuch, B., Sosman, J., Staehler, M., Vázquez Estévez, S., Simsek, B.,…Bex, A. (2023). Adjuvant nivolumab plus ipilimumab versus placebo for localised renal cell carcinoma after nephrectomy (CheckMate 914): a double-blind, randomised, phase 3 trial. *Lancet*, *401*(10379), 821-832. <https://doi.org/10.1016/s0140-6736(22)02574-0>

Nice, K. A., Thompson, J., Zhao, H., Seneviratne, S., Zapata-Diomedi, B., Garcia, L., Hunter, R. F., Reis, R. S., Hallal, P. C., Millett, C., Wang, R., & Stevenson, M. (2025). Effects of city design on transport mode choice and exposure to health risks during and after a crisis: a retrospective observational analysis. *Lancet Planet Health*, *9*(6), e467-e479. <https://doi.org/10.1016/s2542-5196(25)00088-9>

Nikolaeva, A., & Versnel, J. (2022). Analytical observational study evaluating global pandemic preparedness and the effectiveness of early COVID-19 responses in Ethiopia, Nigeria, Singapore, South Korea, Sweden, Taiwan, UK and USA. *BMJ Open*, *12*(2), e053374. <https://doi.org/10.1136/bmjopen-2021-053374>

Nowicka, P., Ek, A., Jurca-Simina, I. E., Bouzas, C., Argelich, E., Nordin, K., García, S., Vasquez Barquero, M. Y., Hoffer, U., Reijs Richards, H., Tur, J. A., Chirita-Emandi, A., & Eli, K. (2022). Explaining the complex impact of the Covid-19 pandemic on children with overweight and obesity: a comparative ecological analysis of parents' perceptions in three countries. *BMC Public Health*, *22*(1), 1000. <https://doi.org/10.1186/s12889-022-13351-1>

Parker, C. C., James, N. D., Brawley, C. D., Clarke, N. W., Ali, A., Amos, C. L., Attard, G., Chowdhury, S., Cook, A., Cross, W., Dearnaley, D. P., Douis, H., Gilbert, D. C., Gilson, C., Gillessen, S., Hoyle, A., Jones, R. J., Langley, R. E., Malik, Z. I.,…Sydes, M. R. (2022). Radiotherapy to the prostate for men with metastatic prostate cancer in the UK and Switzerland: Long-term results from the STAMPEDE randomised controlled trial. *PLoS Med*, *19*(6), e1003998. <https://doi.org/10.1371/journal.pmed.1003998>

Patch, M., Jacobi-Dorbeck, A., Rodney, T., Kelen, G., Campbell, J. C., Rubin, L. H., Wagner, C., Perrin, N., & Gill, J. (2025). Examining acquired brain injury-associated symptoms and fluid-based biomarkers in females surviving intimate partner violence: An observational pilot study protocol. *Womens Health (Lond)*, *21*, 17455057251320717. <https://doi.org/10.1177/17455057251320717>

Patil, V., Noronha, V., Dhumal, S. B., Joshi, A., Menon, N., Bhattacharjee, A., Kulkarni, S., Ankathi, S. K., Mahajan, A., Sable, N., Nawale, K., Bhelekar, A., Mukadam, S., Chandrasekharan, A., Das, S., Vallathol, D., D'Souza, H., Kumar, A., Agrawal, A.,…Prabhash, K. (2020). Low-cost oral metronomic chemotherapy versus intravenous cisplatin in patients with recurrent, metastatic, inoperable head and neck carcinoma: an open-label, parallel-group, non-inferiority, randomised, phase 3 trial. *Lancet Glob Health*, *8*(9), e1213-e1222. <https://doi.org/10.1016/s2214-109x(20)30275-8>

Poolman, M., Roberts, J., Wright, S., Hendry, A., Goulden, N., Holmes, E. A., Byrne, A., Perkins, P., Hoare, Z., Nelson, A., Hiscock, J., Hughes, D., O'Connor, J., Foster, B., Reymond, L., Healy, S., Lewis, P., Wee, B., Johnstone, R.,…Wilkinson, C. (2020). Carer administration of as-needed subcutaneous medication for breakthrough symptoms in people dying at home: the CARiAD feasibility RCT. *Health Technol Assess*, *24*(25), 1-150. <https://doi.org/10.3310/hta24250>

Porter, C. M., Wechsler, A. M., Hime, S. J., & Naschold, F. (2019). Adult Health Status Among Native American Families Participating in the Growing Resilience Home Garden Study. *Prev Chronic Dis*, *16*, E113. <https://doi.org/10.5888/pcd16.190021>

Powles, T., Yuen, K. C., Gillessen, S., Kadel, E. E., 3rd, Rathkopf, D., Matsubara, N., Drake, C. G., Fizazi, K., Piulats, J. M., Wysocki, P. J., Buchschacher, G. L., Jr., Alekseev, B., Mellado, B., Karaszewska, B., Doss, J. F., Rasuo, G., Datye, A., Mariathasan, S., Williams, P., & Sweeney, C. J. (2022). Atezolizumab with enzalutamide versus enzalutamide alone in metastatic castration-resistant prostate cancer: a randomized phase 3 trial. *Nat Med*, *28*(1), 144-153. <https://doi.org/10.1038/s41591-021-01600-6>

Puertas-Gonzalez, J. A., Mariño-Narvaez, C., Romero-Gonzalez, B., Sanchez-Perez, G. M., & Peralta-Ramirez, M. I. (2022). Online cognitive behavioural therapy as a psychological vaccine against stress during the COVID-19 pandemic in pregnant women: A randomised controlled trial. *J Psychiatr Res*, *152*, 397-405. <https://doi.org/10.1016/j.jpsychires.2022.07.016>

Pyatak, E. A., Ali, A., Khurana, A. R., Lee, P. J., Sideris, J., Fox, S., Diaz, J., Granados, G., Blanchard, J., McGuire, R., Salazar Plascencia, E., Salcedo-Rodriguez, E., Flores-Garcia, J., Linderman, M., Taylor, E. E., Tapia, V., Nnoli, N., Sequeira, P. A., Freeby, M. J., & Raymond, J. K. (2023). Research design and baseline participant characteristics of the Resilient, Empowered, Active Living with Diabetes - Telehealth (REAL-T) Study: A randomized controlled trial for young adults with type 1 diabetes. *Contemp Clin Trials*, *135*, 107386. <https://doi.org/10.1016/j.cct.2023.107386>

Ramasamy, M. N., Minassian, A. M., Ewer, K. J., Flaxman, A. L., Folegatti, P. M., Owens, D. R., Voysey, M., Aley, P. K., Angus, B., Babbage, G., Belij-Rammerstorfer, S., Berry, L., Bibi, S., Bittaye, M., Cathie, K., Chappell, H., Charlton, S., Cicconi, P., Clutterbuck, E. A.,…Pollard, A. J. (2021). Safety and immunogenicity of ChAdOx1 nCoV-19 vaccine administered in a prime-boost regimen in young and old adults (COV002): a single-blind, randomised, controlled, phase 2/3 trial. *Lancet*, *396*(10267), 1979-1993. <https://doi.org/10.1016/s0140-6736(20)32466-1>

Ranatunga, I., & Jayaratne, K. (2020). Proportion of unplanned pregnancies, their determinants and health outcomes of women delivering at a teaching hospital in Sri Lanka. *BMC Pregnancy Childbirth*, *20*(1), 667. <https://doi.org/10.1186/s12884-020-03259-2>

Reyes, L. F., Garcia-Gallo, E., Murthy, S., Fuentes, Y. V., Serrano, C. C., Ibáñez-Prada, E. D., Lee, J., Rojek, A., Citarella, B. W., Gonçalves, B. P., Dunning, J., Rätsep, I., Viñan-Garces, A. E., Kartsonaki, C., Rello, J., Martin-Loeches, I., Shankar-Hari, M., Olliaro, P. L., & Merson, L. (2023). Major adverse cardiovascular events (MACE) in patients with severe COVID-19 registered in the ISARIC WHO clinical characterization protocol: A prospective, multinational, observational study. *J Crit Care*, *77*, 154318. <https://doi.org/10.1016/j.jcrc.2023.154318>

Rotheram-Borus, M. J., Christodoulou, J., Hayati Rezvan, P., Comulada, W. S., Gordon, S., Skeen, S., Stewart, J., Almirol, E., & Tomlinson, M. (2019). Maternal HIV does not affect resiliency among uninfected/HIV exposed South African children from birth to 5 years of age. *Aids*, *33 Suppl 1*(Suppl 1), S5-s16. <https://doi.org/10.1097/qad.0000000000002176>

Schierhout, G., Praveen, D., Patel, B., Li, Q., Mogulluru, K., Ameer, M. A., Patel, A., Clifford, G. D., Joshi, R., Heritier, S., Maulik, P., & Peiris, D. (2021). Why do strategies to strengthen primary health care succeed in some places and fail in others? Exploring local variation in the effectiveness of a community health worker managed digital health intervention in rural India. *BMJ Glob Health*, *6*(Suppl 5). <https://doi.org/10.1136/bmjgh-2021-005003>

Schöbi, N., Sanchez, C., Welzel, T., Bamford, A., Webb, K., Rojo, P., Tremoulet, A., Atkinson, A., Schlapbach, L. J., & Bielicki, J. A. (2024). Swissped-RECOVERY: masked independent adjudication for the interpretation of non-randomised treatment in a two-arm open-label randomised controlled trial (methylprednisolone vs immunoglobulins) in Paediatric Inflammatory Multisystem Syndrome Temporally Associated with SARS-CoV-2 (PIMS-TS) involving 10 secondary and tertiary paediatric hospitals in Switzerland. *BMJ Open*, *14*(4), e078137. <https://doi.org/10.1136/bmjopen-2023-078137>

Shinde, V., Lombard Koen, A., Hoosain, Z., Archary, M., Bhorat, Q., Fairlie, L., Lalloo, U., Masilela, M. S. L., Moodley, D., Hanley, S., Fouche, L. F., Louw, C., Tameris, M., Singh, N., Goga, A., Dheda, K., Grobbelaar, C., Joseph, N., Lombaard, J. J.,…Madhi, S. A. (2024). Immunogenicity and safety following a homologous booster dose of a SARS-CoV-2 recombinant spike protein vaccine with Matrix-M(TM) adjuvant (NVX-CoV2373) versus a primary series in people living with and without HIV-1 infection in South Africa: A randomized crossover phase 2a/2b trial. *Hum Vaccin Immunother*, *20*(1), 2425147. <https://doi.org/10.1080/21645515.2024.2425147>

Sibanda, E. L., Mangenah, C., Neuman, M., Tumushime, M., Watadzaushe, C., Mutseta, M. N., Maringwa, G., Dirawo, J., Fielding, K. L., Johnson, C., Ncube, G., Taegtmeyer, M., Hatzold, K., Corbett, E. L., Terris-Prestholt, F., & Cowan, F. M. (2021). Comparison of community-led distribution of HIV self-tests kits with distribution by paid distributors: a cluster randomised trial in rural Zimbabwean communities. *BMJ Glob Health*, *6*(Suppl 4). <https://doi.org/10.1136/bmjgh-2021-005000>

Siedner, M. J., Kraemer, J. D., Meyer, M. J., Harling, G., Mngomezulu, T., Gabela, P., Dlamini, S., Gareta, D., Majozi, N., Ngwenya, N., Seeley, J., Wong, E., Iwuji, C., Shahmanesh, M., Hanekom, W., & Herbst, K. (2020). Access to primary healthcare during lockdown measures for COVID-19 in rural South Africa: an interrupted time series analysis. *BMJ Open*, *10*(10), e043763. <https://doi.org/10.1136/bmjopen-2020-043763>

Singh, J. A., Joseph, A., Baker, J., Richman, J. S., Shaneyfelt, T., Saag, K. G., & Eisen, S. (2021). SToRytelling to Improve Disease outcomes in Gout (STRIDE-GO): a multicenter, randomized controlled trial in African American veterans with gout. *BMC Med*, *19*(1), 265. <https://doi.org/10.1186/s12916-021-02135-w>

Singleton, A. C., Raeside, R., Hyun, K. K., Hayes, M., Sherman, K. A., Elder, E., Redfern, J., & Partridge, S. R. (2023). A National Health and Wellness SMS Text Message Program for Breast Cancer Survivors During COVID-19 (EMPOWER-SMS COVID-19): Mixed Methods Evaluation Using the RE-AIM Framework. *J Med Internet Res*, *25*, e45164. <https://doi.org/10.2196/45164>

Sisti, L. G., Di Napoli, A., Petrelli, A., Rossi, A., Diodati, A., Menghini, M., Mirisola, C., & Costanzo, G. (2021). COVID-19 Impact in the Italian Reception System for Migrants during the Nationwide Lockdown: A National Observational Study. *Int J Environ Res Public Health*, *18*(23). <https://doi.org/10.3390/ijerph182312380>

Snowsill, T. M., Stathi, A., Green, C., Withall, J., Greaves, C. J., Thompson, J. L., Taylor, G., Gray, S., Johansen-Berg, H., Bilzon, J. L. J., de Koning, J. L., Bollen, J. C., Moorlock, S. J., Western, M. J., Guralnik, J. M., Rejeski, W. J., Fox, K. R., & Medina-Lara, A. (2022). Cost-effectiveness of a physical activity and behaviour maintenance programme on functional mobility decline in older adults: an economic evaluation of the REACT (Retirement in Action) trial. *Lancet Public Health*, *7*(4), e327-e334. <https://doi.org/10.1016/s2468-2667(22)00030-5>

Sokhi, R. S., Singh, V., Querol, X., Finardi, S., Targino, A. C., Andrade, M. F., Pavlovic, R., Garland, R. M., Massagué, J., Kong, S., Baklanov, A., Ren, L., Tarasova, O., Carmichael, G., Peuch, V. H., Anand, V., Arbilla, G., Badali, K., Beig, G.,…Zavala, M. (2021). A global observational analysis to understand changes in air quality during exceptionally low anthropogenic emission conditions. *Environ Int*, *157*, 106818. <https://doi.org/10.1016/j.envint.2021.106818>

Sol, J., Ortega-Bravo, M., Portero-Otín, M., Piñol-Ripoll, G., Ribas-Ripoll, V., Artigues-Barberà, E., Butí, M., Pamplona, R., & Jové, M. (2024). Human lifespan and sex-specific patterns of resilience to disease: a retrospective population-wide cohort study. *BMC Med*, *22*(1), 17. <https://doi.org/10.1186/s12916-023-03206-w>

Sorsdahl, K., Stein, D. J., Pasche, S., Jacobs, Y., Kader, R., Odlaug, B., Richter, S., Myers, B., & Grant, J. E. (2021). A novel brief treatment for methamphetamine use disorders in South Africa: a randomised feasibility trial. *Addict Sci Clin Pract*, *16*(1), 3. <https://doi.org/10.1186/s13722-020-00209-3>

Stevens, E. R., Roberts, E., Kuczynski, H. C., & Boden-Albala, B. (2019). Stroke Warning Information and Faster Treatment (SWIFT): Cost-Effectiveness of a Stroke Preparedness Intervention. *Value Health*, *22*(11), 1240-1247. <https://doi.org/10.1016/j.jval.2019.06.003>

Tay, A. K., Mung, H. K., Miah, M. A. A., Balasundaram, S., Ventevogel, P., Badrudduza, M., Khan, S., Morgan, K., Rees, S., Mohsin, M., & Silove, D. (2020). An Integrative Adapt Therapy for common mental health symptoms and adaptive stress amongst Rohingya, Chin, and Kachin refugees living in Malaysia: A randomized controlled trial. *PLoS Med*, *17*(3), e1003073. <https://doi.org/10.1371/journal.pmed.1003073>

Thiem, V. D., Anh, D. D., Ha, V. H., Van Thom, N., Thang, T. C., Mateus, J., Carreño, J. M., Raghunandan, R., Huong, N. M., Mercer, L. D., Flores, J., Escarrega, E. A., Raskin, A., Thai, D. H., Van Be, L., Sette, A., Innis, B. L., Krammer, F., & Weiskopf, D. (2025). Safety and immunogenicity of an inactivated recombinant Newcastle disease virus vaccine expressing SARS-CoV-2 spike: A randomised, comparator-controlled, phase 2 trial. *Vaccine*, *44*, 126542. <https://doi.org/10.1016/j.vaccine.2024.126542>

Travers, J., Romero-Ortuno, R., Ní Shé, É., & Cooney, M. T. (2022). Involving older people in co-designing an intervention to reverse frailty and build resilience. *Fam Pract*, *39*(1), 200-206. <https://doi.org/10.1093/fampra/cmab084>

Valentine, S. K., Jacelon, C. S., & Cavanagh, S. J. (2023). NYS Nonprofit Hospital Assessment and Response to Environmental Pollution as Community Health Need: Prevalence in Community Benefit Practices. *J Public Health Manag Pract*, *29*(6), E245-e252. <https://doi.org/10.1097/phh.0000000000001789>

van Heerden, A., Szpiro, A., Ntinga, X., Celum, C., van Rooyen, H., Essack, Z., & Barnabas, R. (2023). A Sequential Multiple Assignment Randomized Trial of scalable interventions for ART delivery in South Africa: the SMART ART study. *Trials*, *24*(1), 32. <https://doi.org/10.1186/s13063-022-07025-x>

Vargas, S. M., Wennerstrom, A., Alfaro, N., Belin, T., Griffith, K., Haywood, C., Jones, F., Lunn, M. R., Meyers, D., Miranda, J., Obedin-Maliver, J., Pollock, M., Sherbourne, C. D., Springgate, B. F., Sugarman, O. K., Rey, E., Williams, C., Williams, P., & Chung, B. (2019). Resilience Against Depression Disparities (RADD): a protocol for a randomised comparative effectiveness trial for depression among predominantly low-income, racial/ethnic, sexual and gender minorities. *BMJ Open*, *9*(10), e031099. <https://doi.org/10.1136/bmjopen-2019-031099>

Vickery, N., Stephens, T., du Toit, L., van Straaten, D., Pearse, R., Torborg, A., Rolt, L., Puchert, M., Martin, G., & Biccard, B. (2021). Understanding the performance of a pan-African intervention to reduce postoperative mortality: a mixed-methods process evaluation of the ASOS-2 trial. *Br J Anaesth*, *127*(5), 778-788. <https://doi.org/10.1016/j.bja.2021.07.001>

Voysey, M., Clemens, S. A. C., Madhi, S. A., Weckx, L. Y., Folegatti, P. M., Aley, P. K., Angus, B., Baillie, V. L., Barnabas, S. L., Bhorat, Q. E., Bibi, S., Briner, C., Cicconi, P., Collins, A. M., Colin-Jones, R., Cutland, C. L., Darton, T. C., Dheda, K., Duncan, C. J. A.,…Pollard, A. J. (2021). Safety and efficacy of the ChAdOx1 nCoV-19 vaccine (AZD1222) against SARS-CoV-2: an interim analysis of four randomised controlled trials in Brazil, South Africa, and the UK. *Lancet*, *397*(10269), 99-111. <https://doi.org/10.1016/s0140-6736(20)32661-1>

Wachtler, B., Beese, F., Demirer, I., Haller, S., Pförtner, T. K., Wahrendorf, M., Grabka, M. M., & Hoebel, J. (2024). Education and pandemic SARS-CoV-2 infections in the German working population - the mediating role of working from home. *Scand J Work Environ Health*, *50*(3), 168-177. <https://doi.org/10.5271/sjweh.4144>

Williams, L. R., Emary, K. R. W., Phillips, D. J., Hay, J., Larwood, J. P. J., Ramasamy, M. N., Pollard, A. J., Grassly, N. C., & Voysey, M. (2024). Implementation and adherence to regular asymptomatic testing in a COVID-19 vaccine trial. *Vaccine*, *42*(21), 126167. <https://doi.org/10.1016/j.vaccine.2024.126167>

Wilson, K. E., Michaud, T. L., Almeida, F. A., Schwab, R. J., Porter, G. C., Aquilina, K. H., Brito, F. A., Golden, C. A., Dressler, E. V., Kittel, C. A., Harvin, L. N., Boggs, A. E., Katula, J. A., & Estabrooks, P. A. (2021). Using a population health management approach to enroll participants in a diabetes prevention trial: reach outcomes from the PREDICTS randomized clinical trial. *Transl Behav Med*, *11*(5), 1066-1077. <https://doi.org/10.1093/tbm/ibab010>

Yoseph, A., Teklesilasie, W., Guillen-Grima, F., & Astatkie, A. (2024). Effect of community health education on mothers' knowledge of obstetric danger signs and birth preparedness and complication readiness practices in southern Ethiopia: A cluster randomized controlled trial. *PLoS One*, *19*(11), e0312267. <https://doi.org/10.1371/journal.pone.0312267>

Zeigler, C., Jacobs, Z. G., Schwanke Khilji, S. U., Rice, M. K., Frink, B., & Carney, P. A. (2024). Increasing Care for Underserved Communities Through a Global Health Residency Training Program. *Ann Glob Health*, *90*(1), 70. <https://doi.org/10.5334/aogh.4501>

Zeleke, G. T., Avan, B. I., Dubale, M. A., & Schellenberg, J. (2024). Effect of the data-informed platform for health intervention on the culture of data use for decision-making among district health office staff in North Shewa Zone, Ethiopia: a cluster-randomised controlled trial. *BMC Med Inform Decis Mak*, *24*(1), 190. <https://doi.org/10.1186/s12911-024-02597-x>

Zhang, D., Jia, Y., Chen, Y., Meng, G., Zhuang, X., Chen, L., Wang, D., & Zhang, Y. P. (2022). Effect of an online resourcefulness training in improving psychological well-being of front-line medical staff: a quasi-experimental study. *BMC Psychol*, *10*(1), 217. <https://doi.org/10.1186/s40359-022-00920-7>
