# Supplementary material for: COVID‐19 Pandemic: A Comprehensive Meta‐Review of Global Impacts, Responses, and Future Preparedness
Source: Clin Respir J. 2025 Nov 21;19(11):e70134. doi: 10.1111/crj.70134 (PMC12635497; doi:10.1111/crj.70134)
Supplement: Supplementary file 2 — Data S2: Supporting information. [file CRJ-19-e70134-s002.docx]

**Study_Characteristics_Data 24 studies**

**Study 1**

| **Study ID (Author, Year, Country/Region)** | **Study Design & Population** | **Focus Area (Impact / Response / Preparedness / Equity)** | **Exposure / Intervention / Policy Studied** | **Outcomes (with Statistical Data)** | **Equity Dimension / Vulnerable Groups** | **Key Findings (Summary of Results)** |
| --- | --- | --- | --- | --- | --- | --- |
| Global Health Research Group on Children’s Non-Communicable Diseases Collaborative, **2022**; **Global (39 countries; HIC & LMIC)** | Multicentre international **observational cohort** (mixed retrospective/prospective); **N=1,660** paediatric cancer patients <18 y with ALL, NHL/HL, Wilms, sarcoma, retinoblastoma, glioma, medulloblastoma, neuroblastoma; care between **Mar–Dec 2020** | **Impact** (service disruption, mortality), **Response** (treatment modifications), **Equity** (LMIC vs HIC disparities) | System-level impact of the **COVID-19 pandemic** on paediatric oncology care: delays/alterations to chemotherapy/radiotherapy/surgery; COVID-19 positivity; health-system drivers (infrastructure, policy, staffing, lockdowns) | **30-day mortality:** LMIC vs HIC OR **12.1** (95% CI **2.93–50.3**), *p*<0.001; **Adjusted 30-day OR** for LMIC vs HIC **15.6** (95% CI **3.7–65.8**), *p*<0.001. **90-day mortality:** LMIC vs HIC OR **7.9** (95% CI **3.2–19.7**), *p*<0.001. **Treatment changes:** **219/1660** (13.2%) affected; **83.1%** in LMICs. | **Equity:** Marked excess mortality and more frequent treatment alterations in **LMICs**; vulnerable group = children with cancer, particularly in **LMIC** settings; instances of **treatment abandonment** reported. | COVID-19 **exacerbated existing disparities**: substantially higher short-term mortality in LMICs and more service disruptions; nonetheless, **most patients continued standard care**, demonstrating system **resilience**, but policy/infrastructure constraints disproportionately affected LMICs. |

**Study 2**

| **Study ID Country/Region)** | **Study Design & Population** | **Focus Area (Impact / Response / Preparedness / Equity)** | **Exposure / Intervention / Policy Studied** | **Outcomes (with Statistical Data)** | **Equity Dimension / Vulnerable Groups** | **Key Findings (Summary of Results)** |
| --- | --- | --- | --- | --- | --- | --- |
| (Aggarwal et al., 2024); **Global** (Africa, Asia, Europe, USA; 114 sites) | **Secondary analysis** of randomized trial cohorts; adults ≥18 y hospitalized with acute COVID-19; **N=2,625** (median age 57; 58% male); standardized baseline clinical and biomarker data | **Impact** (90-day mortality risk); signal for **Equity** by region | Baseline biomarkers & severity: **plasma SARS-CoV-2 nucleocapsid antigen** (Ag), upper-respiratory **viral RNA**, **IL-6**, CRP, ALC, eGFR; pulmonary status (O₂ flow/NIV/HFNC); vaccination & anti-S/anti-N antibodies; region & period | Multivariable Cox (aHR, 95% CI): Ag ≥ 4500 ng/L **2.07 (1.29–3.34)** vs <200; viral RNA <35,000 **2.42 (1.09–5.34)**, ≥35,000 **2.84 (1.29–6.28)** vs undetectable; **IL-6 >5.8 ng/L 2.54 (1.74–3.70)**; pulmonary status vs no O₂: O₂<4 L **1.84 (1.06–3.22)**, O₂≥4 L **4.41 (2.63–7.39)**, **NIV/HFNC 11.30 (6.46–19.75)**; renal impairment **1.77 (1.29–2.42)** | **Regional disparity:** higher adjusted mortality in **Africa** vs USA (**aHR 3.88; 2.34–6.43**), lower in **Europe** (**0.33; 0.20–0.57**). Vulnerable group: adults with severe disease/organ dysfunction | Higher **viral antigenemia**, **upper-airway viral RNA**, **inflammation (IL-6)**, **renal dysfunction**, and greater **respiratory support** independently predict 90-day mortality; antibody positivity associates with lower risk; findings robust to sensitivity analyses (placebo-only; exclusion of largest trial). |

**Study 3**

| **Study ID (Author, Year, Country/Region)** | **Study Design & Population** | **Focus Area (Impact / Response / Preparedness / Equity)** | **Exposure / Intervention / Policy Studied** | **Outcomes (with Statistical Data)** | **Equity Dimension / Vulnerable Groups** | **Key Findings (Summary of Results)** |
| --- | --- | --- | --- | --- | --- | --- |
| (Ambrose et al., 2023); **USA (4 health systems)** | **Retrospective observational** cohort using de-identified EHRs; COVID-19 positive outpatients ≥12 y eligible for nMAbs; **N=167,183** (treated **25,241** [15.1%], untreated **141,942**); index Nov 2020–Jan 2022 | **Impact** (14-day hospitalization; 30-day mortality), **Response** (nMAb effectiveness), **Equity** (SDoH disparities) | Receipt of **neutralizing monoclonal antibodies** (bamlanivimab±etesevimab, casirivimab-imdevimab, sotrovimab) vs no nMAb; SDoH: race/ethnicity, insurance, marital status, Area Deprivation Index, population density; vaccination, comorbidities | **Access (propensity model, adj. ORs):** Black/African American vs White non-Hispanic **0.86 (0.82–0.91)**; Medicaid vs private **0.89 (0.84–0.93)**; divorced/widowed lower odds; highest ADI lower odds. **Disease risk (DRS, adj. ORs):** Medicaid **1.18 (1.13–1.24)**; cystic fibrosis **3.47 (1.54–7.80)**; severe renal failure **3.38 (2.81–4.08)**; vaccination ↓ risk. **Effectiveness (MSMs with IPTW, MI):** nMAbs **reduced 14-day hospitalization and 30-day mortality across most SDoH subgroups** (several statistically significant). | **Disadvantaged SDoH groups** (Black/African American, Medicaid, divorced/widowed, rural, high-ADI) had **lower treatment odds** and **higher risk** of poor outcome. | **nMAbs beneficial across SDoH strata**, but **access disparities persisted**; clinical factors (age, comorbidities, vaccination) had stronger effects on outcome than SDoH alone. |

**Study 4**

| **Study ID (Author, Year, Country/Region)** | **Study Design & Population** | **Focus Area (Impact / Response / Preparedness / Equity)** | **Exposure / Intervention / Policy Studied** | **Outcomes (with Statistical Data)** | **Equity Dimension / Vulnerable Groups** | **Key Findings (Summary of Results)** |
| --- | --- | --- | --- | --- | --- | --- |
| (Ataguba et al., 2023); **Global**: HIV/AIDS analyses **217 countries (2000–2021)**; COVID-19 analyses **151 countries (2020–2021)**; sub-analyses: **Africa-only** and **excluding-Africa** | **Observational ecological** national datasets (World Bank, UNAIDS, Economist Intelligence Unit excess mortality, World Inequality Database). Linear regressions with regional (and, for HIV/AIDS, year) fixed effects; covariates: income category, **health expenditure per capita**, UNAIDS region | **Impact** (HIV incidence, AIDS mortality, COVID-19 excess mortality); **Equity** (income inequality); **Preparedness** (policy implications for unequal societies) | **Exposure:** Income inequality (Gini index). **Controls:** income level, health spending, region; fixed effects as above | **HIV incidence:** Gini positively associated in global, Africa-only, and excluding-Africa samples (**p<0.01**). **AIDS mortality:** positive association in all samples (**p<0.01**). **COVID-19 excess mortality:** positive association with Gini for global and excluding-Africa samples (**p<0.05**); **not significant** for Africa. **Scaled effects (Duan smearing):** 25-pp Gini reduction → HIV incidence ↓ **0.14/1000** (global) and **2.11/1000** (Africa) next year; AIDS mortality ↓ **6.58**, **11.45**, **17.39** per 100,000 (global, Africa, excluding-Africa). | **Equity focus:** Higher Gini (greater inequality) linked to worse pandemic outcomes; Africa COVID-19 model non-significant (likely sample/measurement constraints). | **More unequal countries experience higher HIV incidence, AIDS mortality, and COVID-19 excess deaths** after adjustment. Findings support **inequality-aware preparedness/response** to avoid reinforcing disparities. |

**Study 5**

| **Study ID (Author, Year, Country/Region)** | **Study Design & Population** | **Focus Area (Impact / Response / Preparedness / Equity)** | **Exposure / Intervention / Policy Studied** | **Outcomes (with Statistical Data)** | **Equity Dimension / Vulnerable Groups** | **Key Findings (Summary of Results)** |
| --- | --- | --- | --- | --- | --- | --- |
| (Berthaud et al., 2024); USA & Canada | Primary clinical study (booster phase within phase 2/3 KidCOVE); observer-blinded components described. Children 6 mo–5 y (n=153; booster 10 µg) and 6–11 y (n=2,519; booster 25 µg) who previously completed a 2-dose mRNA-1273 primary series. Per-protocol immunogenicity subsets SARS-CoV-2–negative at booster: n=76 (younger), n=145 (older). | **Response** (vaccination booster; safety & immunogenicity) | mRNA-1273 booster ≥6 months post-primary series (10 µg for 6 mo–5 y; 25 µg for 6–11 y); noninferiority vs young adult (18–25 y) responses after primary series. | **Immunogenicity (ancestral/D614G):** 6 mo–5 y: GMC 341→5457 (Day 29); **GMR vs adults** 3.90 (95% CI 3.16–4.81); **SRR 100% (72/72)**; GMFR 15.8 (95% CI 12.8–19.4). 6–11 y: GMC 434→5561; **GMR 3.97 (95% CI 3.41–4.63); SRR 100% (137/137)**; GMFR 12.8 (95% CI 11.3–14.6). **Safety:** Solicited local AEs 49.0% (younger) & 90.3% (older); systemic AEs 63.4% & 61.3%; grade ≥3 systemic AEs 3.3% & 4.6%; **no myocarditis/pericarditis, no deaths; SAEs 0.7% (younger) & 0.4% (older), none vaccine-related.** | Pediatric populations (6 mo–11 y); demographics reported (sex, race/ethnicity incl. Black, Asian, Hispanic/Latino). No dedicated inequity analysis. | Pediatric mRNA-1273 booster elicited robust neutralizing responses meeting prespecified noninferiority vs young adults and had an acceptable safety profile with no new safety signals, supporting booster use as a mitigation strategy in children. |

**Study 6**

| **Study ID (Author, Year, Country/Region)** | **Study Design & Population** | **Focus Area (Impact / Response / Preparedness / Equity)** | **Exposure / Intervention / Policy Studied** | **Outcomes (with Statistical Data)** | **Equity Dimension / Vulnerable Groups** | **Key Findings (Summary of Results)** |
| --- | --- | --- | --- | --- | --- | --- |
| (Bhattacharyya et al., 2022); Odisha, India | Observational ecological analysis using **30 districts** (1 May 2020–15 Apr 2021); **25 indicators** across five themes aggregated to theme-specific COVID-19 Vulnerability Indices (cVIs) and overall VI; associations with time-varying reproduction number (**instantaneous RRR**; also vRvRvR) assessed via **Bayesian model averaging** linear regression. | **Impact** (transmission growth), **Response/Preparedness** (health-system readiness), **Equity** (socioeconomic & access disparities in LMIC). | Exposures: Theme-specific cVIs—socio-demographic (cVISD), housing & hygiene (cVIHH), availability of healthcare (cVIAH), preparedness for COVID-19 (cVIPC), epidemiological factors (cVIEF); overall VI. | **Primary outcome: iR.** Theme-level BMA (iR as response): cVIHH **PIP=0.45, β=0.18 (SD=0.26)**; cVIPC **PIP=0.42, β=0.18 (SD=0.27)**; cVIEF **PIP=0.42, β=0.17 (SD=0.26)**; cVISD **PIP=0.20, β=−0.04 (SD=0.15)**. Selected within-theme indicators: clean fuel **PIP=0.59, β=0.37 (SD=0.42)**; public hospitals **PIP=0.62, β=0.35 (SD=0.36)**; population **PIP=0.66, β=−0.35 (SD=0.32)**; literacy **PIP=0.48, β=0.23 (SD=0.30)**. Spatial clustering documented; most districts had **iR > 1** in early 2021. | Equity operationalized via socio-economic deprivation, housing/hygiene deficits, and health-care access constraints; highlights rural/peripheral, resource-limited districts. | **Housing & hygiene, COVID-19 preparedness, and epidemiological burden positively associated with higher transmission (iR);** socio-demographic composite had low importance. Identifies high-vulnerability clusters (south/south-west), informing **targeted resource allocation and mitigation strategies** for current and future waves. |

**Study 7**

| **Study ID (Author, Year, Country/Region)** | **Study Design & Population** | **Focus Area (Impact / Response / Preparedness / Equity)** | **Exposure / Intervention / Policy Studied** | **Outcomes (with Statistical Data)** | **Equity Dimension / Vulnerable Groups** | **Key Findings (Summary of Results)** |
| --- | --- | --- | --- | --- | --- | --- |
| (Bradbury et al., 2022); 105 sites in 8 countries (Canada, France, Germany, India, Italy, Nepal, Netherlands, UK) | Adaptive platform **RCT**, open-label; critically ill adults with COVID-19 requiring ICU respiratory/cardiovascular organ support. **N=1,557 randomized** (aspirin n=565; P2Y12 inhibitor n=455; control n=529); **1,549 completed**; 90-day follow-up. Concurrent standard thromboprophylaxis. | **Response** (therapeutic intervention during pandemic care) | Up to 14 days: **aspirin 75–100 mg qd** or **P2Y12 inhibitor** (mostly clopidogrel 75 mg qd; few ticagrelor/prasugrel) **vs no antiplatelet**; adaptive pooling of aspirin/P2Y12 after equivalence trigger. | **Primary:** Organ support–free days to day 21: median 7 in both groups; **adj. OR 1.02 (95% CrI 0.86–1.23)** → **95.7% probability of futility**. **Hospital survival:** 71.5% vs 67.9%; **adj. OR 1.27 (0.99–1.62)**; absolute diff. 5% (CrI −0.2 to 9.5%); **97% probability of efficacy**. **90-day survival:** **HR 1.22 (1.06–1.40)**; 99.7% probability improved survival. **Major bleeding ≤14 d:** 2.1% vs 0.4%; **adj. OR 2.97 (1.23–8.28)**; absolute risk ↑ 0.8% (0.1–2.7%); 99.4% probability of harm. **Thrombosis or death composite:** **adj. OR 0.70 (0.54–0.90)**. | Multinational cohort includes **LMIC settings (India, Nepal)**; race/ethnicity collected; high-bleeding-risk patients excluded. | Antiplatelet therapy **did not improve** organ support–free days (primary endpoint) but likely **improved survival** (secondary Bayesian probabilities) at the cost of **increased major bleeding**; domain stopped for futility on the primary outcome. Findings inform risk–benefit of antiplatelets with anticoagulation in ICU COVID-19 care. |

**Study 8**

| **Study ID (Author, Year, Country/Region)** | **Study Design & Population** | **Focus Area (Impact / Response / Preparedness / Equity)** | **Exposure / Intervention / Policy Studied** | **Outcomes (with Statistical Data)** | **Equity Dimension / Vulnerable Groups** | **Key Findings (Summary of Results)** |
| --- | --- | --- | --- | --- | --- | --- |
| (Bravo et al., 2022); Belgium, Brazil, Colombia, Philippines, South Africa | Phase 2/3 double-blind RCT; adults ≥18 y, generally healthy or with stable comorbidities; safety set n=30,128; per-protocol SARS-CoV-2–naïve n=12,355 (vaccine 6,251; placebo 6,104) | Response (vaccination); Impact (clinical outcomes) | Two IM doses SCB-2019 (30 µg + CpG-1018 1.5 mg + alum 0.75 mg) vs placebo, 21 days apart | Primary VE (any symptomatic COVID-19, ≥14 d post-dose2, naïve): 67.2% (95.72% CI 54.3–76.8); Moderate-to-severe: 83.7% (97.86% CI 55.9–95.4); Severe: 100% (97.86% CI 25.3–100.0); Hospitalization: 100% (95% CI 42.7–100.0); Variant-specific VE (any severity): Delta 78.7% (95% CI 57.3–90.4), Gamma 91.8% (44.9–99.8), Mu 58.6% (13.3–81.5); Full analysis set VE (any severity): 66.7% (95% CI 55.5–75.4). Safety (phase 2 subset n≈1600): higher injection-site pain (35.7% vs 10.3% after dose 1), otherwise similar solicited systemic AEs; serious AEs rare and balanced. | Includes LMIC sites (Brazil, Colombia, Philippines, South Africa); high-risk comorbidity subgroup: VE any severity 65.9% (95% CI 35.7–82.9); moderate-to-severe 78.7% (22.3–96.1). | SCB-2019 provided significant protection against symptomatic COVID-19 (including Delta/Gamma/Mu periods), with strong protection against moderate-to-severe disease, severe disease, and hospitalization; acceptable reactogenicity profile; efficacy consistent across sex and BMI; insufficient power in ≥60 y for precise estimates. |

**Study 9**

| **Study ID (Author, Year, Country/Region)** | **Study Design & Population** | **Focus Area (Impact / Response / Preparedness / Equity)** | **Exposure / Intervention / Policy Studied** | **Outcomes (with Statistical Data)** | **Equity Dimension / Vulnerable Groups** | **Key Findings (Summary of Results)** |
| --- | --- | --- | --- | --- | --- | --- |
| (Gonçalves et al., 2022), Multinational (28–30 countries; major contributors South Africa, UK; admissions 1 Oct 2021–28 Feb 2022) | Multinational observational cohort of hospitalized, laboratory-confirmed/suspected COVID-19 patients; prospectively collected ISARIC CRFs; n=129,196 total; primary analyses restricted to admissions in 2-month windows before Omicron ≥10% vs after Omicron ≥90% at country level (n=103,061). Mixed-effects logistic and Cox models; adjustment for age, sex, vaccination; random intercepts by country/site. | **Impact** (clinical presentation, severity/mortality); **Response** (effect of vaccination status on outcomes). | Period defined by population-level Omicron dominance (pre-Omicron vs Omicron) inferred from GISAID lineage frequencies (10%/90% thresholds); sensitivity at 80% threshold; comparison also validated in subset with individual-level variant calls. | Primary: 14-day in-hospital mortality (and 28-day); Composite: death or IMV. Adjusted OR for 14-day mortality during Omicron period vs pre-Omicron: **0.65–0.68** (e.g., Model I: 0.65 [0.62–0.69]; Model II: 0.67 [0.61–0.75]; Model IV: 0.64 [0.59–0.69]). Vaccination associated with lower 14-day mortality (e.g., OR 0.60 [0.55–0.65] in Model II; 0.59 [0.54–0.65] in Model IV). Older age, male sex, hypertension/diabetes/cardiac disease increased risk (e.g., hypertension OR 1.26–1.29). Descriptive: lower frequencies of common symptoms during Omicron; composite death/IMV: 10,049/67,383. Survival analyses yielded concordant findings. | Includes broad geography (incl. Global South); age-stratified results; vaccination status captured (binary). Country-level heterogeneity explored; random effects account for site-level variation. | After Omicron emergence, hospitalized cases showed fewer typical symptoms and **lower short-term mortality** vs pre-Omicron, independent of age/sex/vaccination/comorbidities. Sensitivity analyses (alternative thresholds; country restrictions; excluding non-COVID admissions) were consistent. Limitations: variant misclassification risk using population-level frequencies; missingness in onset/outcome dates; changing country contribution over time. |

**Study 10**

| **Study ID (Author, Year, Country/Region)** | **Study Design & Population** | **Focus Area (Impact / Response / Preparedness / Equity)** | **Exposure / Intervention / Policy Studied** | **Outcomes (with Statistical Data)** | **Equity Dimension / Vulnerable Groups** | **Key Findings (Summary of Results)** |
| --- | --- | --- | --- | --- | --- | --- |
| (Heath et al., 2023), United Kingdom | Phase 3 RCT; randomized 1:1 NVX-CoV2373 vs placebo; adults 18–84 y across 33 UK sites; per-protocol efficacy set n=13,989 (6,989 vaccine; 7,000 placebo); follow-up up to 7.5 months (median 4.5); censoring at unblinding/receipt of other vaccines/crossover. | **Response** (vaccine intervention); **Impact** (clinical disease prevention). | Two 5-µg doses NVX-CoV2373 IM, 21 days apart, vs normal saline placebo; blinded crossover after placebo-controlled phase. | **Primary efficacy:** 24/6,989 vs 134/7,000 symptomatic PCR-confirmed cases ≥7 days post-dose 2 → VE **82.7%** (95% CI 73.3–88.8); incidence 9.48 vs 54.85 per 1,000 person-years. **Severe COVID-19:** VE **100%** (95% CI 17.9–100.0) (all 6 severe cases in placebo). **Moderate/severe:** VE 79.2% (66.7–87.0). **Asymptomatic infection:** VE **76.3%** (57.4–86.8). **Any infection (symptomatic or asymptomatic):** VE **82.5%** (75.0–87.7). **Safety:** unsolicited AEs 27.4% vs 21.8%; severe AEs 1.2% vs 1.1%; SAEs 0.8% vs 0.8%; 7 deaths total (4 vaccine, 3 placebo), none vaccine-related. **Immunogenicity:** strong anti-S IgG and neutralization rises; IFN-γ ELISpot GMFRs 8.4–16.5 by peptide pool. | Subgroup analyses by age (≥65 vs <65), sex, race, comorbidity status, and influenza coadministration; inclusion of participants with stable chronic conditions (incl. HIV on ART). Older adults showed lower antibody titers; VE remained high but trended lower with age. | Two-dose NVX-CoV2373 provided high protection against symptomatic, asymptomatic, and severe COVID-19 through >6 months with favorable safety; modest waning suggests benefit of boosters. Variant context predominantly Alpha with emerging Delta during follow-up. |

**Study 11**

| **Study ID (Author, Year, Country/Region)** | **Study Design & Population** | **Focus Area (Impact / Response / Preparedness / Equity)** | **Exposure / Intervention / Policy Studied** | **Outcomes (with Statistical Data)** | **Equity Dimension / Vulnerable Groups** | **Key Findings (Summary of Results)** |
| --- | --- | --- | --- | --- | --- | --- |
| (Jennings et al., 2024), South Africa (Cape Town) | Retrospective observational cohort using routine program data (Provincial Health Data Centre). Two annual cohorts: **pre-COVID-19** (1 Oct 2018–30 Sep 2019; n=27,481 DS-TB diagnoses) and **during-COVID-19** (1 Apr 2020–31 Mar 2021; n=19,800 DS-TB diagnoses). | **Impact** (health service disruption due to COVID-19). | COVID-19 period/lockdown exposure (pre vs during pandemic; first lockdown 26 Mar 2020) affecting TB services; no clinical intervention. | Diagnosed DS-TB: **–28%** relative change (**95% CI 27.4–28.5**). Initial loss to follow-up (ILTFU): **13.4% → 15.2%** (**p<0.001**). Post-treatment loss (PTL): **25.2% → 26.1%** (**p=0.033**). Treatment success rate: **74.8% → 73.9%** (relative –1.3%). Cascade success (diagnosed → treatment success): **64.8% → 62.7%** (**p<0.001**). Stratified results provided by sex, age, HIV status, prior TB, and diagnostic mode. | Disaggregations by **sex (male/female)**, **age** (<15 vs ≥15), **HIV status**, **TB history** (new vs retreatment), and **mode of diagnosis** (bacteriological vs clinical). Higher relative ILTFU increase in **males** and **people living with HIV (PLHIV)**. | COVID-19 substantially disrupted TB services: fewer diagnoses (–28%), increased ILTFU (+13.2% relative) and PTL (+3.7% relative), with declines in treatment and cascade success. PLHIV and males were disproportionately affected. Findings support **TB recovery plans** to strengthen linkage and retention during/after public-health disruptions. |

**Study 12**

| **Study ID (Author, Year, Country/Region)** | **Study Design & Population** | **Focus Area (Impact / Response / Preparedness / Equity)** | **Exposure / Intervention / Policy Studied** | **Outcomes (with Statistical Data)** | **Equity Dimension / Vulnerable Groups** | **Key Findings (Summary of Results)** |
| --- | --- | --- | --- | --- | --- | --- |
| (López-Macías, Torres, Armenta-Copca, Wacher, Castro-Castrezana, et al., 2025), **Mexico** | Mixed phase II: initial single-blind safety run-in then **randomized, double-blind, placebo-controlled** booster trial. Adults previously vaccinated against SARS-CoV-2 with baseline anti-spike IgG <1,200 U/mL. Randomized **n=158**: AVX-IM 41 (analyzed 36), AVX-IN 40 (analyzed 36), placebo-IM 40, placebo-IN 37; placebo later received open-label AZ/ChAdOx-1-S at day 28. Follow-up to day 365. | **Response** (booster immunization), **Impact** (immune augmentation), **Equity** (local manufacturing in LMIC). | **AVX/COVID-12** (NDV-based) booster **IM 0.5 mL** or **IN 0.2 mL** vs placebo; subsequent AZ booster in placebo group. | **Neutralizing antibody ≥2.5-fold rise (Day 14):** IM AVX vs IM placebo—Wuhan 78.9% vs 17.9% (p<0.0001); Alpha 84.2% vs 25.6% (p<0.0001); Beta 50.0% vs 17.9% (p=0.003); Delta 55.3% vs 17.9% (p=0.001); Omicron BA.2 50.0% vs 12.8% (p<0.0001); BA.5 71.1% vs 35.9% (p=0.002). IN AVX vs IN placebo—Wuhan 82.1% vs 24.3% (p<0.0001); Alpha 76.9% vs 29.7% (p<0.0001); Beta 48.7% vs 21.6% (p=0.012); Delta 35.9% vs 16.2% (p=0.045); BA.2 42.1% vs 16.2% (p=0.013); BA.5 65.8% vs 48.6% (p=0.102). **Binding IgG ≥4-fold rise:** IM 73.7% vs 10.0% (p<0.0001); IN 51.3% vs 16.2% (p=0.001). **Cellular immunity:** IFN-γ GM increased to day 180/365 (e.g., IM 235.8→1160.1 pg/mL, p=0.01; IN 241.4→951.2/1011.3, p=0.03/0.01). **Safety (Day 7):** Any AE higher with AVX than placebo (e.g., IM 92.68% vs 77.5%); local AESIs higher IM AVX vs IM placebo (p≈0.003–0.008); **no alarming safety signals; SAEs not vaccine-related**. | LMIC context; subgroup data by age/sex, route (IM/IN), prior primary series platform; small numbers with **HIV** included. | Locally manufactured **AVX** booster (IM and IN) was **safe** and **immunogenic**, significantly boosting neutralizing antibodies across variants (including **Omicron BA.2/BA.5**; stronger IM effect) and sustaining **IFN-γ** responses to 6–12 months. Supports **equitable**, locally producible booster options; clinical efficacy against infection was not a primary endpoint. |

**Study 13**

| **Study ID (Author, Year, Country/Region)** | **Study Design & Population** | **Focus Area (Impact / Response / Preparedness / Equity)** | **Exposure / Intervention / Policy Studied** | **Outcomes (with Statistical Data)** | **Equity Dimension / Vulnerable Groups** | **Key Findings (Summary of Results)** |
| --- | --- | --- | --- | --- | --- | --- |
| (López-Macías, Torres, Armenta-Copca, Wacher, Galindo-Fraga, et al., 2025), Mexico | Parallel-group RCT, double-blind, active-controlled, noninferiority (phase 2 immunogenicity/futility; phase 3 immunobridging). Adults ≥18 with prior vaccination and/or prior infection. Safety cohort N=4,056; immunobridging AVX n=705 vs AZ n=712; follow-up to 180 days. | **Response** (booster vaccination); **Impact** (immune augmentation); **Equity** (LMIC, local manufacturing). | Single-dose AVX/COVID-12 (NDV-LaSota HexaPro-S) **IM booster** (10^8 TCID50) vs **AZ/ChAdOx-1-S** active control. | **Primary noninferiority:** Neutralizing GMT ratio AVX/AZ day 14 = **0.96 (95% CI 0.85–1.06)** → meets WHO NI (LL≥0.67). **Seroconversion (≥2× rise):** **58.15% AVX vs 55.75% AZ**; difference +2.4% (95% CI −2.7 to 7.5). **Exploratory neutralization vs Omicron BA.2/BA.5:** significant rises vs baseline at days 14/90/180. **Cellular:** ↑ IFN-γ–producing **CD8+ T cells** day 14 vs baseline for AVX (**p=0.006**). **Symptomatic COVID-19 ≤180d:** **5.5% AVX (37/673)** vs **6.3% AZ (42/667)**; incidence/1000 days 0.29 vs 0.32; **log-rank p=0.42**. **Safety (7d):** any AE **51.37% AVX** vs **54.27% AZ** (NS); local AESIs **25.64% vs 20.61% (p=0.002)**; systemic mild AESIs **15.89% vs 19.55% (p=0.008)**; VAAEs **17.27% vs 13.03% (p=0.01)**; **no severe COVID-19, hospitalizations, or deaths** in either arm. | Conducted in an **LMIC** (Mexico); broad adult inclusion incl. comorbidities; addresses **access and local manufacturing** needs for Global South. | AVX booster was **safe**, **immunogenic**, and **noninferior** to AZ for neutralizing responses; boosted CD8+ IFN-γ responses; similar short-term symptomatic COVID-19 incidence. Supports **equitable**, locally manufactured booster options for LMICs and future preparedness. |

**Study 14**

| **Study ID (Author, Year, Country/Region)** | **Study Design & Population** | **Focus Area (Impact / Response / Preparedness / Equity)** | **Exposure / Intervention / Policy Studied** | **Outcomes (with Statistical Data)** | **Equity Dimension / Vulnerable Groups** | **Key Findings (Summary of Results)** |
| --- | --- | --- | --- | --- | --- | --- |
| (Mayland et al., 2021), United Kingdom | Observational cross-sectional open online survey (Qualtrics); bereaved relatives/friends (≥18 y) of decedents in the UK during the initial pandemic period; n=278 completed (of 384 who accessed); respondent mean age 53.4 y; 78.0% female; nearly all White British; decedents predominantly older; 58.5% died in nursing/residential homes. | **Impact** (bereavement experience, emotional support, communication, dignity), **Response** (health/social care communication practices, visiting policies), **Equity** (gender differences; access to visits/virtual contact). | Public health **visiting restrictions** and communication practices; setting of care (home, care home, hospital); ability to visit (yes/no); respondent and decedent characteristics (age, gender, relationship; dementia; place of death). | Descriptives and regression: 56.5% unable to visit in last days; 33.7% had no online contact. Perceived adequate support: 54.5% yes, 45.5% no. **Univariate** predictors of adequate support: male respondents 29.8% vs 11.9% (p=0.001); able to visit 55.3% vs 35.5% (p=0.001). **Multivariate logistic regression:** male vs female **OR 2.90 (95% CI 1.44–5.94; p=0.003)**; able to visit vs not **OR 2.20 (95% CI 1.30–3.75; p=0.004)**. Additional items: told likely to die soon 75.0%; told what to expect when dying 30.9%; “right place” of death 75.2%. | **Gender** (men reported higher perceived support); **Setting** (care homes heavily represented); **Age/Dementia** (many decedents older; 53.2% dementia). Ethnic representation limited (nearly all White British), indicating potential generalisability concerns. | Visiting restrictions compounded distress and were independently associated with lower perceived support; clearer, proactive communication and enabling presence/connection (in person or virtual) linked to better perceived support. Perceptions of dignity/respect and communication were less favorable than pre-COVID CODE™ studies. Men more likely than women to report adequate support. Limitations: convenience sampling; missing data; limited ethnic diversity. |

**Study 15**

| **Study ID (Author, Year, Country/Region)** | **Study Design & Population** | **Focus Area (Impact / Response / Preparedness / Equity)** | **Exposure / Intervention / Policy Studied** | **Outcomes (with Statistical Data)** | **Equity Dimension / Vulnerable Groups** | **Key Findings (Summary of Results)** |
| --- | --- | --- | --- | --- | --- | --- |
| (Mediavilla et al., 2023), Spain (Madrid & Catalonia) | Parallel, multicentre RCT; HCWs with psychological distress (K10 ≥ 16) employed by regional health departments; n=232 randomized (115 stepped-care; 117 eCAU); mean age 37.5 y; 86% women; ~93% involved in direct COVID-19 care; ~59% ever infected with COVID-19. | **Response** (mental-health intervention for HCWs), **Impact** (anxiety/depression/PTSD symptoms). | Stepped-care programme (WHO **Doing What Matters** [5 weeks, guided self-help] → step-up to **Problem Management Plus** [~5–6 weeks] if still distressed) delivered remotely by trained non-specialists, versus enhanced care as usual (PFA-informed resource guidance). | **Primary (PHQ-ADS at week 21):** baseline-adjusted mean difference = **4.4** (95% CI 2.1–6.7); standardized effect size **0.8** (95% CI 0.4–1.2) favoring intervention. **Interim:** week 7 diff **3.4** (1.7–5.2); week 13 diff **5.9** (3.6–8.1). **Secondary:** improvements on PHQ-9 and GAD-7 at t2–t4; PCL-5 improved except non-significant at week 7; mixed-effects ITT models with robust SEs; no serious adverse events. | **HCWs** (occupational risk group); majority female; frontline exposure considered; high-income setting but applicable to stressed workforces. | A brief, scalable stepped-care psychological programme **reduced anxiety/depression** among HCWs versus eCAU with moderate-to-large effects by 21 weeks; safe, remote delivery by trained non-specialists supports **policy relevance and scalability** for future health crises. Limitations include self-report outcomes, short follow-up, and absence of an active comparator. |

**Study 16**

| **Study ID (Author, Year, Country/Region)** | **Study Design & Population** | **Focus Area (Impact / Response / Preparedness / Equity)** | **Exposure / Intervention / Policy Studied** | **Outcomes (with Statistical Data)** | **Equity Dimension / Vulnerable Groups** | **Key Findings (Summary of Results)** |
| --- | --- | --- | --- | --- | --- | --- |
| (Nice et al., 2025); Global (507 cities across Africa, Asia, Europe, North & South America, Oceania) | Retrospective observational **ecological** study; 507 cities with complete 2015–2020 NO₂/PM₂.₅ series; graph neural network used to characterise city design/transport networks; linked to 2020 Google/Apple mobility indices and reported COVID-19 cases. | **Impact** (air pollution & health risk); **Response** (NPIs, mobility restrictions, mode shifts); **Preparedness/Resilience** (city design sustaining low pollution/risks); **Equity** (includes Global South cities). | Non-pharmaceutical interventions → mobility reductions and **modal shifts** (public/active↔private vehicles) interacting with **urban design typologies** across phases: pre-pandemic, entry, mid-crisis, recovery (2020). | Entry→mid-crisis mean **NO₂ −3.76 ppb** (observed 12.63 vs predicted 16.39); mean **PM₂.₅ −9.76 μg/m³** (observed 29.03 vs predicted 38.79). If maintained long term, estimated **relative risk (RR) reductions**: NO₂—**all-cause mortality −1.5% (95% CI 2.2–3.0)**; CVD mortality −4.1% (2.6–6.0); respiratory mortality −1.9% (0.8–3.0). PM₂.₅—**all-cause mortality −18.9% (13.2–25.0)**; asthma −46.8% (18.7–65.5); IHD morbidity −0.25% (0.2–0.3). Recovery: car-oriented regions (Americas/Oceania) rebounded to/beyond baseline pollution and road trauma; Japan/South Korea sustained transit share with lower pollution/risks. | Inclusion of **LMIC/Global South** cities (e.g., Latin America, Africa, South Asia) with higher dependence on public transport and varying car reliance; heterogeneity in exposure/benefit. | Early NPIs yielded **transient** reductions in transport-related pollution and estimated health risks; **car-centric designs** enabled a shift to private vehicles in recovery, eroding gains and aligning with higher road injury; **transit-oriented designs** (Japan/South Korea) showed **greater resilience**, sustaining lower pollution and risks. Ecological design and modeled risk estimates noted as limitations. |

**Study 17**

| **Study ID (Author, Year, Country/Region)** | **Study Design & Population** | **Focus Area (Impact / Response / Preparedness / Equity)** | **Exposure / Intervention / Policy Studied** | **Outcomes (with Statistical Data)** | **Equity Dimension / Vulnerable Groups** | **Key Findings (Summary of Results)** |
| --- | --- | --- | --- | --- | --- | --- |
| *(Puertas-Gonzalez et al., 2022)t*; Spain (national, pandemic “state of alert”; recruitment Sep 2020–Jun 2021) | Single-blind, 3-arm RCT; N=207 pregnant women (12–28 weeks’ gestation). Randomised 1:1:1 to online CBT (o-CBT n=70), online psychological support (o-PS n=69), usual care (UC n=68). ITT with last-observation-carried-forward; baseline balance tested by ANOVA/χ². | **Impact** (stress, psychopathology), **Response** (online intervention), **Equity** (pregnancy as vulnerable group; remote delivery during restrictions). | 8 weekly group sessions (1.5–2 h) via Google Meet. o-CBT: psychoeducation, relaxation, cognitive restructuring, social skills, emotion regulation, optimism; o-PS: psychoeducation/emotional support; UC: routine antenatal care. | **Primary:** PDQ (pregnancy-specific stress): Group×Time F(2,197.48)=6.27, *p*=.002, η²=.06; o-CBT Δ↓ with *d*=0.62. PSS-14 (perceived stress): F(2,197.61)=5.02, *p*=.007, η²=.05; o-CBT *d*=0.76. IVE: no interaction. CD-RISC (resilience): F(2,192.78)=7.08, *p*=.001, η²=.07; o-CBT improved. **Secondary (SCL-90-R):** Obsessions-compulsions F=8.13, *p*=.001, η²=.08; Depression F=6.23, *p*=.002, η²=.06; Anxiety F=7.05, *p*=.001, η²=.07; o-CBT effect sizes approx. *d*=0.51 (OBS), 0.61 (DEP), 0.54 (ANX); o-PS small/none; UC minimal. | Pregnant women during COVID-19 (vulnerable group); remote format improves access during mobility restrictions. | o-CBT significantly reduced pregnancy-specific and perceived stress and improved resilience versus o-PS/UC; medium reductions in anxiety, depression, and obsessions-compulsions versus comparators. Demonstrates efficacy and practical feasibility of remote CBT for pregnant women under pandemic conditions. |

**Study 18**

| **Study ID (Author, Year, Country/Region)** | **Study Design & Population** | **Focus Area (Impact / Response / Preparedness / Equity)** | **Exposure / Intervention / Policy Studied** | **Outcomes (with Statistical Data)** | **Equity Dimension / Vulnerable Groups** | **Key Findings (Summary of Results)** |
| --- | --- | --- | --- | --- | --- | --- |
| (Reyes et al., 2023); Multinational (54 countries; major contributors UK, Pakistan, India, USA, Brazil; >6,000 from LMICs) | Prospective observational cohort of hospitalized **severe COVID-19** adults; N=49,479; severe = advanced respiratory support and/or vasopressors within 24h; stratified by development of **MACE** during admission. | **Impact** (MACE incidence, mortality, LOS); **Equity** (HIC/LMIC mix). | Patient-level clinical factors and acute treatments (IMV, NIV/HFNC, vasopressors) associated with **MACE** occurrence during hospitalization. | **Incidence:** MACE 17.8% (8,829/49,479). **Risk factors for MACE (multivariable):** vasopressors OR 1.44 (95% CI 1.40–1.47); age OR 1.22 (1.19–1.24); IMV 1.17 (1.14–1.20); CKD 1.09 (1.07–1.11); NIV 1.05 (1.03–1.07); HFNC 1.05 (1.03–1.07); smoking 1.04 (1.02–1.06); prior arrhythmia 1.04 (1.02–1.06); model AUROC≈0.72 (10-fold CV). **Mortality effect:** 28-day mortality 63.1% (MACE) vs 35.6% (non-MACE), *p*<0.001; adjusted OR 1.36 (1.33–1.39). 90-day 69.9% vs 37.8%, *p*<0.001; adjusted OR 1.47 (1.43–1.50); mortality model AUROC≈0.80–0.81. **LOS:** median 13 vs 10 days, *p*<0.001. | Broad geographic inclusion with substantial **LMIC** representation; older adults, CKD/CVD, smokers identified as higher risk. | MACE affects ~1 in 5–6 severe COVID-19 inpatients and is **independently associated** with markedly higher 28- and 90-day mortality and longer LOS; early risk stratification (age, CKD/CVD, early vasopressors/ventilation) is critical for monitoring and management. |

**Study 19**

| **Study ID (Author, Year, Country/Region)** | **Study Design & Population** | **Focus Area (Impact / Response / Preparedness / Equity)** | **Exposure / Intervention / Policy Studied** | **Outcomes (with Statistical Data)** | **Equity Dimension / Vulnerable Groups** | **Key Findings (Summary of Results)** |
| --- | --- | --- | --- | --- | --- | --- |
| (Siedner et al., 2020), South Africa (uMkhanyakude District, rural KwaZulu-Natal) | Observational cohort; interrupted time series across 11 primary care clinics; 46,523 individuals making 89,476 visits; Jan 27–Jun 30, 2020 | Impact (health service utilization); Response (national lockdown policy) | National COVID-19 lockdown levels: transition from pre-lockdown to Level 5 (Mar 27), Level 4 (May 1), Level 3 (Jun 1) | Total visits: −6.7 visits/clinic/day at Level 5 start (95% CI −16.4 to 3.0, ns); Child health visits: −7.1/clinic/day (~60%) at Level 5 start (95% CI −8.9 to −5.3); Age <1: −5.3 (95% CI −6.9 to −3.7); Age 1–5: −5.6 (95% CI −6.7 to −4.4); HIV visits: +8.0/clinic/day at Level 5 start (95% CI 2.1 to 13.8); Family planning: +2.0/clinic/day at Level 3 start (95% CI 0.3 to 3.7); No significant change for NCD visits | Rural, low-income/Global South setting; children <5 years; high HIV-burden community; women of reproductive age | Lockdown caused a large, immediate (>50%) but temporary drop in child health visits that rebounded to near baseline within ~3 months; adult ambulatory and HIV services were largely resilient, with HIV visits increasing post-lockdown; policy disruptions differentially affected child preventive care. |

**Study 20**

| **Study ID (Author, Year, Country/Region)** | **Study Design & Population** | **Focus Area (Impact / Response / Preparedness / Equity)** | **Exposure / Intervention / Policy Studied** | **Outcomes (with Statistical Data)** | **Equity Dimension / Vulnerable Groups** | **Key Findings (Summary of Results)** |
| --- | --- | --- | --- | --- | --- | --- |
| **(Sisti et al., 2021), Italy (national)** | Observational, national cross-sectional survey of reception-centre managers covering 5,038/6,837 facilities (73.7%) and ~59,648 guests (≈70% of all 85,730 migrants), lockdown period **1 Feb–12 Jun 2020**. | **Impact** (incidence, hospitalization); **Equity** (migrants/refugees); **Response** (isolation, public-health actions). | Facility characteristics: **facility type** (CAS vs SIPROIMI), **facility saturation index (FSI; crowding proxy)**, geographic macro-area; centre isolation practices; PPE availability. | **Cumulative incidence** ≈ **400/100,000** overall; **positivity rate** among suspected: **41.8%**; **hospitalization**: **25.9%** (ICU: 2 cases); **no deaths**. Regional/macro-area CIs reported (e.g., Bolzano 6,666.7 per 100,000; 95% CI 4,670.7–8,662.6). **Statistical tests:** χ²/Fisher for distributions; Kruskal–Wallis for FSI by macro-area; Mann–Whitney for FSI (facilities with vs without cases; **p < 0.0001**). | **Migrants and refugees** in Italian reception system (vulnerable, high-risk settings). | Incidence in reception system **similar to** general Italian population overall, with **12% higher** in the North and **~90% lower** elsewhere. **Cases concentrated in CAS (82.4%)**; **higher FSI** in facilities with cases (87.7% vs 78.6%; **p < 0.0001**), suggesting crowding as a risk factor. Isolation often in-facility; variable adequacy of single-room isolation. No COVID-19-related deaths observed during the period. |

**Study 21**

| **Study ID (Author, Year, Country/Region)** | **Study Design & Population** | **Focus Area (Impact / Response / Preparedness / Equity)** |  | **Exposure / Intervention / Policy Studied** | **Outcomes (with Statistical Data)** | **Equity Dimension / Vulnerable Groups** | **Key Findings (Summary of Results)** |
| --- | --- | --- | --- | --- | --- | --- | --- |
| (Thiem et al., 2025), Vietnam (Thai Binh Province) | Randomized, observer-blind, controlled **phase 2 RCT**; adults ≥18 y (mean age ≈49; 49.5% male); N=374 randomized 1:1:1 to COVIVAC 3 µg, COVIVAC 6 µg, or VAXZEVRIA; 2 doses (Day 1, Day 29); follow-up to Day 197; 365 completed. | **Response** (vaccine intervention), **Preparedness** (local egg-based platform), **Equity** (LMIC manufacturer; inclusion of ≥60 y). |  | **Intervention:** COVIVAC (NDV-HXP-S) 3 µg or 6 µg IM ×2; **Comparator:** VAXZEVRIA (ChAdOx1) ×2; age-stratified randomization (18–59; ≥60). | **Neutralizing antibodies (PNA NT50 GMT, Day 43):** 18–59 y: 153.28 (95% CI 124.20–189.15) [3 µg], 176.20 (141.45–220.27) [6 µg], 99.92 (80.80–123.56) [VAXZEVRIA]; ≥60 y: 183.57 (133.40–252.61) [3 µg], 257.87 (181.60–367.18) [6 µg], 79.49 (55.68–113.40) [VAXZEVRIA]. **GMFR Day 43:** 18–59 y: 31.20 (25.14–38.74) [3 µg], 35.80 (29.03–44.15) [6 µg], 18.85 (15.10–23.54) [VAXZEVRIA]; ≥60 y: 37.27 (27.43–50.63), 50.10 (35.46–70.76), 16.11 (11.73–22.13). **GMT ratio (COVIVAC 6 µg/VAXZEVRIA, baseline-seronegatives):** Day 43: 1.77 (95% CI 1.30–2.40) [18–59]; 3.24 (1.98–5.32) [≥60]. Day 197: 1.11 (0.51–2.43) [18–59]; 2.32 (0.69–7.85) [≥60]. **Seroresponse ≥4-fold (Day 43, 18–59):** 89.0% [3 µg], 92.8% [6 µg], 85.4% [VAXZEVRIA]. **Live-virus neutralization:** GMT ratios ~1.5–2.0 favoring COVIVAC. **Binding IgG:** lower with COVIVAC (e.g., GMC ratio 0.38 [0.29–0.50] in 18–59 at Day 43). **Safety:** Solicited reactions mostly mild; **fever ≥38 °C after dose 1:** 0.8% (COVIVAC) vs 22.4% (VAXZEVRIA). Unsolicited AEs 23.2–31.2% across arms; **no vaccine-related AEs**; 6 SAEs, none related. **T cells (subset):** CD4+ AIM+ responses in 90–100% across groups; magnitudes similar; CD8+ low. | **LMIC** context (Vietnam public-sector manufacturer; egg-based production enabling affordability/regional access); inclusion of **older adults (≥60 y)**. | COVIVAC (3 µg/6 µg) produced **higher peak neutralizing GMTs** than VAXZEVRIA at Day 43 with **favorable reactogenicity** (notably less fever after dose 1). Differences narrowed by ~6 months. Findings support **locally manufactured, strain-update-capable vaccines** as viable, **equitable preparedness** tools; booster performance and variant-updated formulations warrant further evaluation. |

**Study 22**

| **Study ID (Author, Year, Country/Region)** | **Study Design & Population** | **Focus Area (Impact / Response / Preparedness / Equity)** | **Exposure / Intervention / Policy Studied** | **Outcomes (with Statistical Data)** | **Equity Dimension / Vulnerable Groups** | **Key Findings (Summary of Results)** |
| --- | --- | --- | --- | --- | --- | --- |
| Voysey et al., 2020; UK, Brazil, South Africa | Blinded randomized controlled trials; adults ≥18 y. Enrolled N=23,848 across 4 trials; primary efficacy population N=11,636 (vaccine n=5,807; control n=5,829). Dosing regimens: LD/SD and SD/SD. Median safety follow-up 3.4 months. | Response (public health vaccination); Impact (clinical outcomes, hospitalisation); Equity (includes Global South sites). | ChAdOx1 nCoV-19 (AstraZeneca) vs control (MenACWY or saline); two doses; LD/SD vs SD/SD schedules. | Primary symptomatic COVID-19 ≥14 days post-dose 2 (seronegative): overall VE 70.4% (95.8% CI 54.8–80.6); SD/SD VE 62.1% (95% CI 41.0–75.7); LD/SD VE 90.0% (95% CI 67.4–97.0). First-dose analysis ≥21 days (SD only): VE 64.1% (95% CI 50.5–73.9). Hospitalisations ≥21 days post-dose 1: 10/10 in control arm (2 severe incl. 1 death). Any NAAT-positive swab: VE 55.7% (95% CI 41.1–66.7). Safety: 175 severe AEs (84 vaccine; 91 control); 3 possibly related (1 vaccine, 1 control, 1 masked). | Inclusion of Brazil and South Africa (Global South); high-exposure healthcare workers; limited ≥56 y representation at interim. | Two-dose ChAdOx1 provided significant protection against symptomatic COVID-19 with no hospitalised cases in vaccinees ≥21 days after first dose; LD/SD showed higher VE than SD/SD in interim data; safety profile comparable to control with few serious events. |

**Study 23**

| **Study ID (Author, Year, Country/Region)** | **Study Design & Population** | **Focus Area (Impact / Response / Preparedness / Equity)** | **Exposure / Intervention / Policy Studied** | **Outcomes (with Statistical Data)** | **Equity Dimension / Vulnerable Groups** | **Key Findings (Summary of Results)** |
| --- | --- | --- | --- | --- | --- | --- |
| (Wachtler et al., 2024), Germany | Observational cross-sectional sero-epidemiologic study; working population aged 18–67; N=6,826 (Nov 2021–Feb 2022) | Impacts; Equity; Response (remote work as mitigation) | Educational attainment (ISCED: very high/high/medium/low); frequency of working from home (none, ≤biweekly, several times/week, daily) | Adjusted PR of infection (low vs very high education): **1.76** (95% CI 1.08–2.88; p=0.023); attenuated to **1.27** (0.78–2.09; p=0.338) after adding remote work. Remote work associated with lower infection risk: several times/week **PR 0.61** (0.43–0.86; p=0.005), daily **PR 0.55** (0.39–0.79; p=0.001). KHB mediation: proportion mediated by remote work—**27% (high)**, **42% (medium)**, **58% (low)** education groups. | Lower educational attainment; also reports on migration experience and urban/rural residence (covariates). Vulnerable group: workers with low education and limited remote-work capacity. | Lower education linked to higher SARS-CoV-2 infection risk; a substantial share of this disparity is mediated by inability to work from home. Increasing remote-work capacity—especially in lower-education occupations—may reduce inequalities and infection risk. |

**Study 24**

| **Study ID (Author, Year, Country/Region)** | **Study Design & Population** | **Focus Area (Impact / Response / Preparedness / Equity)** | **Exposure / Intervention / Policy Studied** | **Outcomes (with Statistical Data)** | **Equity Dimension / Vulnerable Groups** | **Key Findings (Summary of Results)** |
| --- | --- | --- | --- | --- | --- | --- |
| (Williams et al., 2024), UK | Phase 2/3 RCT context (ChAdOx1 nCoV-19 vs MenACWY); 10,811 adults at 19 UK sites; VE subset n=8,548; 356,551 weekly self-swabs over ~13 months | Response; Preparedness; Equity | Weekly home RT-PCR self-swabbing program during vaccine trial; predictors of adherence (age, sex, ethnicity, occupation, BMI, comorbidity); trial milestones (unblinding, post-positive) | Median adherence 75.0% (IQR 42.6–90.9). Odds of returning a swab declined 8.1% per week (OR=0.919, 95% CI 0.917–0.921). After unblinding: 32.0% lower odds; after a positive swab: 56.4% lower odds. Age effect: ≥65 vs <25 yrs OR=25.650 (95% CI 19.979–32.931). Estimated probability of detecting an asymptomatic infection at median adherence: 74.5% (IQR 50.9–78.8); during VE period: 78.8% (IQR 70.3–83.4). Estimated false positives in VE period: ~59 (range 21–96) assuming test specificity ~99.945%. | Age (older higher adherence), sex (female higher), ethnicity (white higher), occupation (HCWs—especially seeing COVID-19 patients—lower), BMI (higher BMI lower adherence) | Long-term regular asymptomatic testing in a large vaccine trial is feasible with high—but time-declining—adherence. Adherence varies by sociodemographics and key trial events, influencing infection detection and potential VE bias; insights inform future trial design and pandemic preparedness strategies. |

Global Health Research Group on Children’s Non-Communicable Diseases Collaborative. Impact of the COVID-19 pandemic on patients with paediatric cancer in low-income, middle-income and high-income countries: a multicentre, international, observational cohort study. BMJ open. 2022 Apr;12(4):e054690. <https://doi.org/10.1136/bmjopen-2021-054690>

Aggarwal, N. R., Nordwall, J., Braun, D. L., Chung, L., Coslet, J., Der, T., Eriobu, N., Ginde, A. A., Hayanga, A. J., Highbarger, H., Holodniy, M., Horcajada, J. P., Jain, M. K., Kim, K., Laverdure, S., Lundgren, J., Natarajan, V., Nguyen, H. H., Pett, S. L.,…Reilly, C. (2024). Viral and Host Factors Are Associated With Mortality in Hospitalized Patients With COVID-19. *Clin Infect Dis*, *78*(6), 1490-1503. <https://doi.org/10.1093/cid/ciad780>

Ambrose, N., Amin, A., Anderson, B., Bertagnolli, M., Campion, F., Chow, D., Danan, R., D'Arinzo, L., Drews, A., Erlandson, K., Fitzgerald, K., Gaspar, F., Gong, C., Hanna, G., Hawley, H., Jones, S., Lopansri, B., Mullen, T., Musser, J.,…Yttri, J. (2023). The Influence of Social Determinants on Receiving Outpatient Treatment with Monoclonal Antibodies, Disease Risk, and Effectiveness for COVID-19. *J Gen Intern Med*, *38*(16), 3472-3481. <https://doi.org/10.1007/s11606-023-08324-y>

Ataguba, J. E., Birungi, C., Cunial, S., & Kavanagh, M. (2023). Income inequality and pandemics: insights from HIV/AIDS and COVID-19-a multicountry observational study. *BMJ Glob Health*, *8*(9). <https://doi.org/10.1136/bmjgh-2023-013703>

Berthaud, V., Creech, C. B., Rostad, C. A., Carr, Q., de Leon, L., Dietrich, M., Gupta, A., Javita, D., Nachman, S., Pinninti, S., Rathore, M., Rodriguez, C. A., Luzuriaga, K., Towner, W., Yeakey, A., Brown, M., Zhao, X., Deng, W., Xu, W.,…Schnyder Ghamloush, S. (2024). Safety and Immunogenicity of an mRNA-1273 Booster in Children. *Clin Infect Dis*, *79*(6), 1524-1532. <https://doi.org/10.1093/cid/ciae420>

Bhattacharyya, R., Burman, A., Singh, K., Banerjee, S., Maity, S., Auddy, A., Rout, S. K., Lahoti, S., Panda, R., & Baladandayuthapani, V. (2022). Role of multiresolution vulnerability indices in COVID-19 spread in India: a Bayesian model-based analysis. *BMJ Open*, *12*(11), e056292. <https://doi.org/10.1136/bmjopen-2021-056292>

Bradbury, C. A., Lawler, P. R., Stanworth, S. J., McVerry, B. J., McQuilten, Z., Higgins, A. M., Mouncey, P. R., Al-Beidh, F., Rowan, K. M., Berry, L. R., Lorenzi, E., Zarychanski, R., Arabi, Y. M., Annane, D., Beane, A., van Bentum-Puijk, W., Bhimani, Z., Bihari, S., Bonten, M. J. M.,…Gordon, A. C. (2022). Effect of Antiplatelet Therapy on Survival and Organ Support-Free Days in Critically Ill Patients With COVID-19: A Randomized Clinical Trial. *Jama*, *327*(13), 1247-1259. <https://doi.org/10.1001/jama.2022.2910>

Bravo, L., Smolenov, I., Han, H. H., Li, P., Hosain, R., Rockhold, F., Clemens, S. A. C., Roa, C., Jr., Borja-Tabora, C., Quinsaat, A., Lopez, P., López-Medina, E., Brochado, L., Hernández, E. A., Reynales, H., Medina, T., Velasquez, H., Toloza, L. B., Rodriguez, E. J.,…Clemens, R. (2022). Efficacy of the adjuvanted subunit protein COVID-19 vaccine, SCB-2019: a phase 2 and 3 multicentre, double-blind, randomised, placebo-controlled trial. *Lancet*, *399*(10323), 461-472. <https://doi.org/10.1016/s0140-6736(22)00055-1>

Gonçalves, B. P., Hall, M., Jassat, W., Balan, V., Murthy, S., Kartsonaki, C., Semple, M. G., Rojek, A., Baruch, J., Reyes, L. F., Dasgupta, A., Dunning, J., Citarella, B. W., Pritchard, M., Martín-Quiros, A., Sili, U., Baillie, J. K., Aryal, D., Arabi, Y.,…Olliaro, P. L. (2022). An international observational study to assess the impact of the Omicron variant emergence on the clinical epidemiology of COVID-19 in hospitalised patients. *Elife*, *11*. <https://doi.org/10.7554/eLife.80556>

Heath, P. T., Galiza, E. P., Baxter, D. N., Boffito, M., Browne, D., Burns, F., Chadwick, D. R., Clark, R., Cosgrove, C. A., Galloway, J., Goodman, A. L., Heer, A., Higham, A., Iyengar, S., Jeanes, C., Kalra, P. A., Kyriakidou, C., Bradley, J. M., Munthali, C.,…Toback, S. (2023). Safety and Efficacy of the NVX-CoV2373 Coronavirus Disease 2019 Vaccine at Completion of the Placebo-Controlled Phase of a Randomized Controlled Trial. *Clin Infect Dis*, *76*(3), 398-407. <https://doi.org/10.1093/cid/ciac803>

Jennings, K., Lembani, M., Hesseling, A. C., Mbula, N., Mohr-Holland, E., Mudaly, V., Smith, M., Osman, M., & Meehan, S. A. (2024). A decline in tuberculosis diagnosis, treatment initiation and success during the COVID-19 pandemic, using routine health data in Cape Town, South Africa. *PLoS One*, *19*(9), e0310383. <https://doi.org/10.1371/journal.pone.0310383>

López-Macías, C., Torres, M., Armenta-Copca, B., Wacher, N. H., Castro-Castrezana, L., Colli-Domínguez, A. A., Rivera-Hernández, T., Torres-Flores, A., Damián-Hernández, M., Ramírez-Martínez, L., la Rosa, G. P., Rojas-Martínez, O., Suárez-Martínez, A., Peralta-Sánchez, G., Carranza, C., Juárez, E., Zamudio-Meza, H., Carreto-Binaghi, L. E., Viettri, M.,…Lozano-Dubernard, B. (2025). Phase II study on the safety and immunogenicity of single-dose intramuscular or intranasal administration of the AVX/COVID-12 "Patria" recombinant Newcastle disease virus vaccine as a heterologous booster against COVID-19 in Mexico. *Vaccine*, *43*(Pt 2), 126511. <https://doi.org/10.1016/j.vaccine.2024.126511>

López-Macías, C., Torres, M., Armenta-Copca, B., Wacher, N. H., Galindo-Fraga, A., Castro-Castrezana, L., Colli-Domínguez, A. A., Cervantes-Trujano, E., Rucker-Joerg, I. E., Lozano-Patiño, F., Rivera-Alcocer, J. J., Simón-Campos, A., Sánchez-Campos, E. A., Aguirre-Rivero, R., Muñiz-Carvajal, A. J., Del Carpio-Orantes, L., Márquez-Díaz, F., Rivera-Hernández, T., Torres-Flores, A.,…Lozano-Dubernard, B. (2025). Phase 2/3 study evaluating safety, immunogenicity, and noninferiority of single booster dose of AVX/COVID-12 vaccine. *Sci Adv*, *11*(26), eadq2887. <https://doi.org/10.1126/sciadv.adq2887>

Mayland, C. R., Hughes, R., Lane, S., McGlinchey, T., Donnellan, W., Bennett, K., Hanna, J., Rapa, E., Dalton, L., & Mason, S. R. (2021). Are public health measures and individualised care compatible in the face of a pandemic? A national observational study of bereaved relatives' experiences during the COVID-19 pandemic. *Palliat Med*, *35*(8), 1480-1491. <https://doi.org/10.1177/02692163211019885>

Mediavilla, R., Felez-Nobrega, M., McGreevy, K. R., Monistrol-Mula, A., Bravo-Ortiz, M. F., Bayón, C., Giné-Vázquez, I., Villaescusa, R., Muñoz-Sanjosé, A., Aguilar-Ortiz, S., Figueiredo, N., Nicaise, P., Park, A. L., Petri-Romão, P., Purgato, M., Witteveen, A. B., Underhill, J., Barbui, C., Bryant, R.,…Ayuso-Mateos, J. L. (2023). Effectiveness of a mental health stepped-care programme for healthcare workers with psychological distress in crisis settings: a multicentre randomised controlled trial. *BMJ Ment Health*, *26*(1). <https://doi.org/10.1136/bmjment-2023-300697>

Nice, K. A., Thompson, J., Zhao, H., Seneviratne, S., Zapata-Diomedi, B., Garcia, L., Hunter, R. F., Reis, R. S., Hallal, P. C., Millett, C., Wang, R., & Stevenson, M. (2025). Effects of city design on transport mode choice and exposure to health risks during and after a crisis: a retrospective observational analysis. *Lancet Planet Health*, *9*(6), e467-e479. <https://doi.org/10.1016/s2542-5196(25)00088-9>

Puertas-Gonzalez, J. A., Mariño-Narvaez, C., Romero-Gonzalez, B., Sanchez-Perez, G. M., & Peralta-Ramirez, M. I. (2022). Online cognitive behavioural therapy as a psychological vaccine against stress during the COVID-19 pandemic in pregnant women: A randomised controlled trial. *J Psychiatr Res*, *152*, 397-405. <https://doi.org/10.1016/j.jpsychires.2022.07.016>

Reyes, L. F., Garcia-Gallo, E., Murthy, S., Fuentes, Y. V., Serrano, C. C., Ibáñez-Prada, E. D., Lee, J., Rojek, A., Citarella, B. W., Gonçalves, B. P., Dunning, J., Rätsep, I., Viñan-Garces, A. E., Kartsonaki, C., Rello, J., Martin-Loeches, I., Shankar-Hari, M., Olliaro, P. L., & Merson, L. (2023). Major adverse cardiovascular events (MACE) in patients with severe COVID-19 registered in the ISARIC WHO clinical characterization protocol: A prospective, multinational, observational study. *J Crit Care*, *77*, 154318. <https://doi.org/10.1016/j.jcrc.2023.154318>

Siedner, M. J., Kraemer, J. D., Meyer, M. J., Harling, G., Mngomezulu, T., Gabela, P., Dlamini, S., Gareta, D., Majozi, N., Ngwenya, N., Seeley, J., Wong, E., Iwuji, C., Shahmanesh, M., Hanekom, W., & Herbst, K. (2020). Access to primary healthcare during lockdown measures for COVID-19 in rural South Africa: an interrupted time series analysis. *BMJ Open*, *10*(10), e043763. <https://doi.org/10.1136/bmjopen-2020-043763>

Sisti, L. G., Di Napoli, A., Petrelli, A., Rossi, A., Diodati, A., Menghini, M., Mirisola, C., & Costanzo, G. (2021). COVID-19 Impact in the Italian Reception System for Migrants during the Nationwide Lockdown: A National Observational Study. *Int J Environ Res Public Health*, *18*(23). <https://doi.org/10.3390/ijerph182312380>

Thiem, V. D., Anh, D. D., Ha, V. H., Van Thom, N., Thang, T. C., Mateus, J., Carreño, J. M., Raghunandan, R., Huong, N. M., Mercer, L. D., Flores, J., Escarrega, E. A., Raskin, A., Thai, D. H., Van Be, L., Sette, A., Innis, B. L., Krammer, F., & Weiskopf, D. (2025). Safety and immunogenicity of an inactivated recombinant Newcastle disease virus vaccine expressing SARS-CoV-2 spike: A randomised, comparator-controlled, phase 2 trial. *Vaccine*, *44*, 126542. <https://doi.org/10.1016/j.vaccine.2024.126542>

Voysey, M., Clemens, S. A. C., Madhi, S. A., Weckx, L. Y., Folegatti, P. M., Aley, P. K., Angus, B., Baillie, V. L., Barnabas, S. L., Bhorat, Q. E., Bibi, S., Briner, C., Cicconi, P., Collins, A. M., Colin-Jones, R., Cutland, C. L., Darton, T. C., Dheda, K., Duncan, C. J. A.,…Pollard, A. J. (2021). Safety and efficacy of the ChAdOx1 nCoV-19 vaccine (AZD1222) against SARS-CoV-2: an interim analysis of four randomised controlled trials in Brazil, South Africa, and the UK. *Lancet*, *397*(10269), 99-111. <https://doi.org/10.1016/s0140-6736(20)32661-1>

Wachtler, B., Beese, F., Demirer, I., Haller, S., Pförtner, T. K., Wahrendorf, M., Grabka, M. M., & Hoebel, J. (2024). Education and pandemic SARS-CoV-2 infections in the German working population - the mediating role of working from home. *Scand J Work Environ Health*, *50*(3), 168-177. <https://doi.org/10.5271/sjweh.4144>

Williams, L. R., Emary, K. R. W., Phillips, D. J., Hay, J., Larwood, J. P. J., Ramasamy, M. N., Pollard, A. J., Grassly, N. C., & Voysey, M. (2024). Implementation and adherence to regular asymptomatic testing in a COVID-19 vaccine trial. *Vaccine*, *42*(21), 126167. <https://doi.org/10.1016/j.vaccine.2024.126167>
