# Supplementary material for: COVID‐19 Pandemic: A Comprehensive Meta‐Review of Global Impacts, Responses, and Future Preparedness
Source: Clin Respir J. 2025 Nov 21;19(11):e70134. doi: 10.1111/crj.70134 (PMC12635497; doi:10.1111/crj.70134)
Supplement: Supplementary file 3 — Data S3: Supporting information. [file CRJ-19-e70134-s001.docx]

**Risk of Bias Analysis of 24 Included Studies**

**Study 1(Observational)**

| **Study ID (Author, Year)** | **Tool Used** | **Confounding** | **Selection of participants** | **Classification of interventions/exposures** | **Deviations from intended interventions** | **Missing data** | **Measurement of outcomes** | **Selection of the reported result** | **Overall RoB** |
| --- | --- | --- | --- | --- | --- | --- | --- | --- | --- |
| ("Impact of the COVID-19 pandemic on patients with paediatric cancer in low-income, middle-income and high-income countries: a multicentre, international, observational cohort study," 2022) | ROBINS-I | Moderate — Nonrandomized, multi-country cohort with likely residual confounding by case-mix and service capacity; protocol-guided analyses referenced but unmeasured system factors remain. | Moderate — Consecutive recruitment across 91 hospitals and 39 countries, but interim report notes some centres (e.g., India) not included yet due to approvals. | Moderate — Exposures/interventions predefined by protocol and external taxonomies (e.g., NCEPOD; country income groups), reducing misclassification but relying on site reporting. | Moderate — Study explicitly tracked pandemic-related changes in intended care pathways and service reorganizations, which could introduce post-baseline deviations. | Moderate — Interim nature and centre-level non-submission imply some missingness/availability constraints, though analyses proceed on available cohort. | Low — Primary outcomes (all-cause mortality at 30 and 90 days) prespecified and routinely ascertainable across sites. | Moderate — A prior protocol and STROBE guidance cited, but selective availability of centres/data may still affect reporting choices. | Moderate |

**Study 2(RCT)**

| **Study ID (Author, Year)** | **Tool Used** | **Randomization process** | **Deviations from intended interventions** | **Missing outcome data** | **Measurement of the outcome** | **Selection of the reported result** | **Overall RoB** |
| --- | --- | --- | --- | --- | --- | --- | --- |
| (Aggarwal et al., 2024) | RoB 2 (RCTs) | Low — Participants were randomized within ACTIV-3/TICO multi-arm, multi-stage platform trials using matched placebos; baseline assessments occurred prior to randomization; allocation concealment and multicenter procedures are described in the parent trials. | Low — Double-blind product assignment with matched placebos; concomitant standard-of-care therapies were permitted and recorded; analyses adjusted for treatment assignment, consistent with effect of assignment. | Low — Outcome follow-up was scheduled to day 90 with censoring at last known status; reasons for loss to follow-up were documented; sensitivity analyses addressed missing baseline covariates. | Low — Primary outcome was mortality, an objective endpoint; ascertainment followed standardized trial procedures; blinding of participants and personnel minimizes differential measurement. | Some concerns — Analytic framework and key covariates were pre-specified, but multiple models, interactions, and sensitivity sets were explored without formal multiplicity control, leaving potential for selective reporting. | Some concerns |

**Study 3(Observational)**

| **Study ID (Author, Year)** | **Tool Used** | **Confounding** | **Selection of participants** | **Classification of interventions/exposures** | **Deviations from intended interventions** | **Missing data** | **Measurement of outcomes** | **Selection of the reported result** | **Overall RoB** |
| --- | --- | --- | --- | --- | --- | --- | --- | --- | --- |
| (Ambrose et al., 2023) | ROBINS-I | Moderate — IPTW with propensity scores including SDoH, vaccination, comorbidities, and utilization reduced baseline imbalance; a disease risk score was developed; unmeasured and time-varying confounding remain plausible given observational EHR design and area-level measures. | Serious — Cohort restricted to non-hospitalized, EUA-eligible patients; access/offer of therapy not recorded; exclusions for other outpatient therapeutics during outcome windows risk conditioning on post-baseline care pathways related to both treatment receipt and outcomes. | Low — Exposure captured as documented receipt of specific outpatient nMAb products in EHR; vaccination status integrated from registries; classification anchored to care setting and administration records. | Moderate — Routine-care context with potential co-interventions and care-seeking differences; offer/decline of treatment unobserved; analyses adjusted for treatment assignment but protocolized adherence not applicable. | Low — Low variable missingness addressed via multiple imputation with prespecified covariates; balance diagnostics reported after weighting. | Low — Hospitalization and mortality ascertained from EHR with standardized definitions; objective outcomes; index-day admissions excluded by design. | Moderate — Analysis plan, variable sets, and subgroup strata described; multiple subgroup and modeling choices without registered protocol or multiplicity control allow selective emphasis among compatible analyses. | Serious |

**Study 4(Observational)**

| **Study ID (Author, Year)** | **Tool Used** | **Confounding** | **Selection of participants** | **Classification of interventions/exposures** | **Deviations from intended interventions** | **Missing data** | **Measurement of outcomes** | **Selection of the reported result** | **Overall RoB** |
| --- | --- | --- | --- | --- | --- | --- | --- | --- | --- |
| (Ataguba et al., 2023) | ROBINS-I | Serious — Ecological, country-level analysis with limited adjustment (health expenditure, income category, region/year fixed effects); substantial potential for unmeasured and time-varying confounders related to governance, health system capacity, pandemic policy, and data quality. | Moderate — Inclusion dependent on availability of national datasets across years and sources; data availability likely correlated with development level and surveillance capacity, potentially influencing exposure and outcomes. | Low — Exposure (national Gini index) defined from a standardized repository with clear operationalization; temporal alignment with outcome periods described. | Low — No protocolized intervention; analytic approach does not involve participant-level adherence or deviations from intended exposure. | Moderate — Variable completeness differs across countries; no explicit imputation for covariates/outcomes beyond use of modeled excess mortality estimates; sample sizes vary by outcome window. | Moderate — Outcomes sourced from UNAIDS and modeled excess mortality (EIU) with known measurement and modeling uncertainties; harmonized definitions applied across countries. | Moderate — Prespecified regression framework and sensitivity analyses reported; multiple sample specifications and transformations without a registered protocol allow flexibility in analytical choices. | Serious |

**Study 5(Observational)**

| **Study ID (Author, Year)** | **Tool Used** | **Confounding** | **Selection of participants** | **Classification of interventions/exposures** | **Deviations from intended interventions** | **Missing data** | **Measurement of outcomes** | **Selection of the reported result** | **Overall RoB** |
| --- | --- | --- | --- | --- | --- | --- | --- | --- | --- |
| (Berthaud et al., 2024) | ROBINS-I | **Serious** — Booster analysis was nonrandomized with no concurrent control; comparison used historical young-adult cohort; booster administered ≥6 months after primary series, introducing time-varying confounding. | **Serious** — Participants were children at 80 US and 8 Canadian sites who had completed the primary series and were offered an optional booster (self-selection); generally healthy, with stable chronic conditions allowed. | **Low** — Exposure clearly defined as intramuscular mRNA-1273 booster with age-specific doses; protocolized administration timetable after primary series completion.  5  5 | **Moderate** — All eligible participants received booster without blinding; standard post-vaccination safety monitoring described; co-interventions beyond routine care not detailed. | **Moderate** — Primary immunogenicity analyses restricted to a per-protocol subset negative for SARS-CoV-2 at booster; safety set included all boosted participants; follow-up to data cutoff may imply attrition. | **Low** — Outcomes included solicited/unsolicited AEs and SAEs; immunogenicity measured at prespecified time points using validated pseudovirus neutralization and MSD binding assays.  5  5 | **Low** — Endpoints, analysis populations, ANCOVA model, and noninferiority framework were prespecified and detailed (with secondary objectives in Supplement). | **Serious** |

**Study 6 (Observationa)**

| **Study ID (Author, Year)** | **Tool Used** | **Confounding** | **Selection of participants** | **Classification of interventions/exposures** | **Deviations from intended interventions** | **Missing data** | **Measurement of outcomes** | **Selection of the reported result** | **Overall RoB** |
| --- | --- | --- | --- | --- | --- | --- | --- | --- | --- |
| (Bhattacharyya et al., 2022) | ROBINS-I | **Serious** — Ecological design at district level with potential unmeasured confounders (mobility, environment, under-reporting) that may bias associations despite regression adjustments. | **Moderate** — All 30 districts in Odisha included, but reliance on existing survey/dashboard data may limit representativeness and completeness. | **Low** — Exposures (cVIs) well-defined using standardized algorithm across 25 indicators grouped into five themes. | **Low** — No active interventions; analytic process (construction of cVIs and modeling) followed prespecified procedures. | **Moderate** — Use of secondary survey and case data with acknowledged under-reporting of cases/deaths and incomplete recency for some indicators. | **Low** — Outcomes (instantaneous R, dispersion measure) clearly defined, with transparent methods and validated computation windows. | **Low** — Outcomes and covariates prespecified, with results reported per protocol and supplemented with full code/data. | **Serious** |

**Study 7(RCT)**

| **Study ID (Author, Year)** | **Tool Used** | **Randomization process** | **Deviations from intended interventions** | **Missing outcome data** | **Measurement of the outcome** | **Selection of the reported result** | **Overall RoB** |
| --- | --- | --- | --- | --- | --- | --- | --- |
| (Bradbury et al., 2022) | RoB 2 | **Some concerns** — Centralized computer randomization and adaptive allocation used; however, site-specific intervention availability and adaptive design could introduce selection imbalances. | **Some concerns** — Open-label design; crossovers permitted if clinically indicated; co-enrollment in other trial domains allowed; analysis followed intention-to-treat but lack of blinding may influence adherence or co-interventions. | **Low** — Very small proportion withdrew consent or lacked outcome data; missingness unrelated to assignment; excluded from analysis with no imputation. | **Low** — Outcomes were objective (mortality, organ support) and bleeding/thrombotic events adjudicated blinded; standardized definitions prespecified. | **Low** — Outcomes and analysis models prespecified in trial protocol; Bayesian adaptive rules and subgroup/sensitivity analyses reported per plan. | **Some concerns** |

**Study 8(RCT)**

| **Study ID (Author, Year)** | **Tool Used** | **Randomization process** | **Deviations from intended interventions** | **Missing outcome data** | **Measurement of the outcome** | **Selection of the reported result** | **Overall RoB** |
| --- | --- | --- | --- | --- | --- | --- | --- |
| (Bravo et al., 2022) | RoB 2 | **Low** — Centralized IRT (Cenduit) randomization, 1:1 allocation with block size six; stratified by age, comorbidity status, and prior COVID-19; randomization lists generated by external unmasked statisticians not involved in analyses; participants and study staff masked. | **Low** — Double-blind trial; vaccine administrators unmasked only for preparation/administration and otherwise not involved; dosing schedule and procedures prespecified; standard care otherwise permitted; monitoring and masking procedures described. | **Low** — Flow diagram and analysis populations defined; exclusions and per-protocol criteria prespecified (e.g., baseline serostatus, early RT-PCR positivity, protocol deviations); handling of missing data described for analysis sets. | **Low** — Case identification via ePRO symptom reporting and weekly self-testing with confirmatory RT-PCR; endpoint adjudication by an independent masked committee; standardized definitions and verification processes detailed. | **Low** — Coprimary endpoints and SAP prespecified; trial registered (EudraCT, ClinicalTrials.gov); interim/final analyses and oversight (DSMB) specified a priori; secondary endpoints detailed. | **Low** |

**Study 9(Observational)**

| **Study ID (Author, Year)** | **Tool Used** | **Confounding** | **Selection of participants** | **Classification of interventions/exposures** | **Deviations from intended interventions** | **Missing data** | **Measurement of outcomes** | **Selection of the reported result** | **Overall RoB** |
| --- | --- | --- | --- | --- | --- | --- | --- | --- | --- |
| (Gonçalves et al., 2022) | ROBINS-I | **Serious** – Adjusted for age, sex, vaccination, with random country intercepts, but vaccination modeled only as binary and comorbidities often missing; authors note residual confounding and context-specific factors may explain findings | **Serious** – Hospital-based recruitment not standardized and locally defined; some patients assumed SARS-CoV-2 positive when test data missing; authors acknowledge potential collider bias in hospital studies. | **Serious** – Exposure classified by country-level Omicron frequency thresholds rather than individual sequencing; misclassification risk acknowledged; periods chosen to mitigate, but still based on population-level data. | **Moderate** – Not an interventional study; care practices and admission reasons varied over time; authors restricted analysis windows to reduce time-varying confounding but deviations in clinical pathways could influence outcomes | **Serious** – Symptom data missing for key settings; 35.5% missing symptom onset (assumed pre-admission); 7.2% missing outcome date; missing vaccination/comorbidity data reduced adjusted samples. | **Low** – Mortality measured from hospital records with defined 14/28-day frameworks; clear rules for inclusion/censoring reported. | **Low** – Two pre-specified SAPs; extensive sensitivity analyses and variant-data comparisons reported, reducing selective reporting concerns. | **Serious** |

**Study 10(RCT)**

| **Study ID (Author, Year)** | **Tool Used** | **Randomization process** | **Deviations from intended interventions** | **Missing outcome data** | **Measurement of the outcome** | **Selection of the reported result** | **Overall RoB** |
| --- | --- | --- | --- | --- | --- | --- | --- |
| (Heath et al., 2023) | RoB 2 | **Low** — Centralized interactive response technology with pregenerated schedules; 1:1 block randomization; stratified by site and age ≥65 years; observer-blinded, placebo-controlled at 33 UK sites. | **Some concerns** — Protocol amendment introduced a blinded crossover; participants could request unblinding to receive authorized vaccines outside the study; observer blinding maintained for assessments; standard care otherwise allowed. | **Low** — Analysis populations and censoring rules prespecified; participants censored at unblinding, receipt of another vaccine, entry into crossover, withdrawal, death, or data cutoff. | **Low** — Symptomatic COVID-19 confirmed by PCR per protocol; asymptomatic infection defined by PCR or anti-N seroconversion after day 35; schedules and laboratory methods prespecified. | **Low** — Primary/secondary endpoints and analysis plan specified a priori; event-driven design and stratification detailed; placebo-controlled period defined prior to blinded crossover. | **Some concerns** |

**Study 11 (Observational)**

| **Study ID (Author, Year)** | **Tool Used** | **Confounding** | **Selection of participants** | **Classification of interventions/exposures** | **Deviations from intended interventions** | **Missing data** | **Measurement of outcomes** | **Selection of the reported result** | **Overall RoB** |
| --- | --- | --- | --- | --- | --- | --- | --- | --- | --- |
| (Jennings et al., 2024) | ROBINS-I | **Serious:** Before–after comparison of two calendar periods with acknowledged secular and service-access changes during lockdown; no multivariable adjustment beyond disaggregation by key covariates, leaving substantial residual confounding. | **Moderate:** Cohort drawn from all public-sector DS-TB diagnoses using a unique identifier with de-duplication; frame excludes private sector and may differ between periods, but inclusion rules are clearly described. | **Low:** Exposure defined by calendar period (pre-COVID vs during-COVID) using routine administrative dates; classification objective and not prone to differential misclassification. | **Low:** No researcher-assigned interventions; routine care context where system changes are intrinsic to the exposure period rather than participant-level deviations. | **Moderate:** Routine dataset with documented variable-level missingness (e.g., HIV “unknown” category) and reliance on administrative linkage; steps taken to mitigate reporting lags but some incompleteness remains. | **Moderate:** Outcomes derived from linked PHDC records with pre-specified operational definitions (treatment initiation, success, ILTFU, PTL); potential variation in ascertainment/documentation across periods during pandemic service disruptions. | **Moderate:** Outcomes and analyses framed a priori within a TB care-cascade and presented with supplementary tables, but no protocol registration or analysis plan to guard against selective reporting. | **Serious** |

**Study 12(RCT)**

| **Study ID (Author, Year)** | **Tool Used** | **Randomization process** | **Deviations from intended interventions** | **Missing outcome data** | **Measurement of the outcome** | **Selection of the reported result** | **Overall RoB** |
| --- | --- | --- | --- | --- | --- | --- | --- |
| (López-Macías, Torres, Armenta-Copca, Wacher, Castro-Castrezana, et al., 2025) | RoB 2 | **Low:** Randomized, double-blind, placebo-controlled phase after safety run-in; allocation balanced via computer-based system; participants assigned unique IDs; blinding maintained until placebo group unblinded at day 28  . | **Some concerns:** Placebo recipients unblinded and crossed over to AZ vaccine at day 28, limiting between-group comparisons beyond this point; otherwise, protocol adherence maintained  . | **Some concerns:** Withdrawals occurred (5 IM, 4 IN); 136 of 158 completed; incomplete follow-up to all planned timepoints; reasons for attrition only partly reported  . | **Low:** Safety and immunogenicity outcomes assessed with standardized methods (clinical AE monitoring, antibody titers, IFN-γ from PBMCs) at prespecified intervals; outcome measurement blinded and consistent  . | **Low:** Protocol registered (RNEC2021-AVXSARSCoV2VAC002; NCT05205746); primary/secondary outcomes and analysis plan prespecified; reporting consistent with objectives  . | **Some concerns** |

**Study 13(RCT)**

| **Study ID (Author, Year)** | **Tool Used** | **Randomization process** | **Deviations from intended interventions** | **Missing outcome data** | **Measurement of the outcome** | **Selection of the reported result** | **Overall RoB** |
| --- | --- | --- | --- | --- | --- | --- | --- |
| (López-Macías, Torres, Armenta-Copca, Wacher, Galindo-Fraga, et al., 2025) | RoB 2 | **Some concerns:** Phase 2/3 parallel-group, double-blind, active-controlled noninferiority RCT with 1:1 allocation; randomization and allocation described in the trial profile, but sequence concealment procedures are not explicitly detailed. | **Low:** Double-blind administration to both arms with protocolized visits and assessments; no planned crossover between randomized groups during the main comparative phase. | **Some concerns:** CONSORT-style profile documents withdrawals and loss to follow-up with reasons; although accounted for, attrition occurred across follow-up. | **Low:** Immunogenicity measured via validated pseudovirus neutralization assays and cellular assays (PBMC flow cytometry) at prespecified timepoints; safety and infection surveillance conducted per protocol. | **Low:** Protocol registered and ethics/regulatory approvals reported; primary noninferiority objectives and analyses defined a priori per WHO guidance. | **Some concerns** |

**Study 14(Observational)**

| **Study ID (Mayland et al., 2021)** | **Tool Used** | **Confounding** | **Selection of participants** | **Classification of interventions/exposures** | **Deviations from intended interventions** | **Missing data** | **Measurement of outcomes** | **Selection of the reported result** | **Overall RoB** |
| --- | --- | --- | --- | --- | --- | --- | --- | --- | --- |
| (Mayland et al., 2021) | ROBINS-I | Associations adjusted for some covariates (age, gender, relationship, ability to visit, dementia, place of death), but unmeasured social/contextual confounders likely remain. **Judgment: Serious** | Convenience and snowball sampling via networks and social media; self-selected participants; predominantly White British, female; limited representativeness. **Judgment: Serious** | Exposures (e.g., visiting restrictions, care setting) self-reported by bereaved relatives using adapted CODE™ tool; no external validation; moderate risk of misclassification. **Judgment: Moderate** | No interventions applied; survey based on recall of experiences; variability in recall introduces potential bias but limited scope for deviation. **Judgment: Moderate** | 20.6% non-completion; demographic-only completers excluded; item non-response allowed; deceased individuals in respondent group older than in non-respondents. **Judgment: Moderate** | Outcomes (perceived support, communication, care quality) measured with adapted CODE™ plus free-text; subjective and recall-dependent; potential for systematic bias in perceptions. **Judgment: Serious** | No preregistration; reported results align with study aims but potential selective emphasis of findings. **Judgment: Moderate** | **Serious** |

**Study 15(RCT)**

| **Study ID (Mediavilla et al., 2023)** | **Tool Used** | **Randomization process** | **Deviations from intended interventions** | **Missing outcome data** | **Measurement of the outcome** | **Selection of the reported result** | **Overall RoB** |
| --- | --- | --- | --- | --- | --- | --- | --- |
| (Mediavilla et al., 2023) | RoB 2 | Randomization via random blocks (sizes 4 and 6) in Castor EDC; allocation sequence concealed from project managers; analysts blinded by coding; participants and providers not blinded. **Judgment: Some concerns** | Remote stepped-care interventions (DWM, PM+) delivered by trained non-specialists; high adherence reported; no contamination reported; lack of blinding of participants/providers could influence responses. **Judgment: Some concerns** | 79.3% completed final follow-up; attrition accounted for in sample size; ITT and sensitivity analyses performed; no evidence of differential missingness by arm. **Judgment: Low** | Outcomes based on self-reported validated scales (PHQ-ADS, PHQ-9, GAD-7, PCL-5); no blinded assessment; potential reporting and social desirability bias. **Judgment: Some concerns** | Prospectively registered trial (NCT04980326) with prespecified protocol and SAP; main outcomes reported; exploratory analyses disclosed; selective reporting unlikely. **Judgment: Low** | **Some concerns** |

**Study 16(Observational)**

| **Study ID (Author, Year)** | **Tool Used** | **Confounding** | **Selection of participants** | **Classification of interventions/exposures** | **Deviations from intended interventions** | **Missing data** | **Measurement of outcomes** | **Selection of the reported result** | **Overall RoB** |
| --- | --- | --- | --- | --- | --- | --- | --- | --- | --- |
| (Nice et al., 2025) | ROBINS-I | Retrospective, ecological city-level analysis across pandemic phases; individual-level covariate adjustment not possible; concurrent policies and contextual factors could confound associations. **Judgment: Serious** | Analytic sample included 507 cities with complete data; exclusion of cities with incomplete series may reduce representativeness. **Judgment: Moderate** | Exposures (city design clusters, mobility, pollution anomalies) defined with standardised data and pipelines; measurement unlikely to misclassify. **Judgment: Low** | No assigned interventions; analysis reflects naturally occurring NPIs and behaviour; no deviation bias relevant. **Judgment: Low** | Cities with incomplete data excluded; no imputation within included series; risk of bias from missingness exists but limited. **Judgment: Moderate** | Outcomes (pollution anomalies, mobility, health risk estimates, COVID-19 cases) measured using validated external datasets and published risk functions; objective measures. **Judgment: Low** | Analytic plan outlined phases and indicators, but no preregistration; multiple comparisons raise selective reporting concerns. **Judgment: Moderate** | Serious |

**Study 17(RCT)**

| **Study ID (Author, Year)** | **Tool Used** | **Randomization process** | **Deviations from intended interventions** | **Missing outcome data** | **Measurement of the outcome** | **Selection of the reported result** | **Overall RoB** |
| --- | --- | --- | --- | --- | --- | --- | --- |
| (Puertas-Gonzalez et al., 2022) | RoB 2 | Participants were randomly allocated (1:1:1) to online CBT, online psychological support, or usual care using a computer-generated sequence in SPSS; assignment was executed by a research assistant not involved in participant data and the trial was registered and CONSORT-reported, with baseline groups described as comparable on key characteristics. **Judgment: Low.** | Interventions comprised 8 weekly synchronous group sessions (o-CBT/o-PS) delivered by licensed psychotherapists; adherence was reported, but no formal fidelity monitoring was described. Given the nature of behavioural group interventions and self-administered questionnaires, participants and providers were likely unblinded, introducing potential performance effects despite standardized manuals. **Judgment: Some concerns.** | An intention-to-treat approach with last observation carried forward was used; attrition and flow were documented with a CONSORT diagram, with lower completion noted in the usual care arm. While imputation was applied, the use of LOCF may not fully address differential missingness mechanisms. **Judgment: Some concerns.** | Outcomes were self-reported psychological scales (e.g., PSS-14, PDQ, CD-RISC, SCL-90-R) administered online using validated instruments with reported internal consistency; however, outcome assessors and participants were not masked, and measures are subjective, increasing risk of measurement bias. **Judgment: Some concerns.** | Primary (stress, resilience) and secondary (psychopathology) outcomes were prespecified, analyses (linear mixed models with post-hoc tests) were described, and results were reported comprehensively; trial registration is noted, but a detailed preregistered analysis plan is not presented, leaving room for selective emphasis. **Judgment: Some concerns.** | Some concerns |

**Study 18(Observational)**

| **Study ID (Author, Year)** | **Tool Used** | **Confounding** | **Selection of participants** | **Classification of interventions/exposures** | **Deviations from intended interventions** | **Missing data** | **Measurement of outcomes** | **Selection of the reported result** | **Overall RoB** |
| --- | --- | --- | --- | --- | --- | --- | --- | --- | --- |
| (Reyes et al., 2023) | ROBINS-I | Adjusted for demographics, comorbidities, admission characteristics, and early treatments, but residual confounding likely due to absence of comprehensive severity scoring and unmeasured contextual factors. **Judgment: Serious** | Inclusion restricted to severe rtPCR-confirmed cases within 24 h; exclusions for incomplete data and missing outcomes could introduce selection bias, though standardized CRFs were applied across countries. **Judgment: Moderate** | Exposures (MACE, comorbidities, treatments) defined using standardized criteria and ISARIC protocols; misclassification unlikely given protocolized definitions. **Judgment: Low** | No assigned interventions; care followed local practice; deviations not applicable as exposures were naturally occurring. **Judgment: Low** | Patients with >30% missing data excluded; some diagnostic data not uniformly available; exclusion approach limits but does not eliminate bias from missingness. **Judgment: Moderate** | Outcomes (MACE, mortality) measured prospectively via standardized CRFs; some variability in diagnostic availability across sites may affect uniformity. **Judgment: Moderate** | Endpoints and modeling approaches described with supplementary results; however, no preregistered statistical analysis plan, raising potential for selective emphasis. **Judgment: Moderate** | Serious |

**Study 19(Observational)**

| **Study ID (Author, Year)** | **Tool Used** | **Confounding** | **Selection of participants** | **Classification of interventions/exposures** | **Deviations from intended interventions** | **Missing data** | **Measurement of outcomes** | **Selection of the reported result** | **Overall RoB** |
| --- | --- | --- | --- | --- | --- | --- | --- | --- | --- |
| (Siedner et al., 2020) | ROBINS-I | Interrupted time series with clinic-level mixed models included fixed effects for day-of-week and random intercepts/slopes; parallel 2019 series used for difference-in-differences to address seasonality and trends. Residual confounding from concurrent shocks (e.g., supply constraints, local outbreaks, transport restrictions) cannot be fully excluded. **Judgment: Moderate** | Frame was all weekday visits at 11 public clinics in one HDSS catchment; weekends and days without research staff were excluded from observation. This setting-based capture minimizes individual self-selection but exclusion of unobserved days may introduce selection bias. **Judgment: Moderate** | Exposure was government-defined lockdown levels (pre-lockdown, Levels 5–3) with a priori dates; stratifications by visit type/age/sex were prespecified. Period classification was objective and protocolized. **Judgment: Low** | No study-assigned interventions; care and patient behavior followed routine practice under national NPIs. Investigators did not influence adherence or implementation. **Judgment: Low** | Unobserved days (weekends/holidays/staff training) were excluded; no imputation for missing observation days. If missingness correlates with utilization patterns, estimates may be biased. **Judgment: Moderate** | Daily visit counts were prospectively recorded by research assistants using standardized procedures and linked to clinic registers; outcome is objective (counts) with consistent definitions across clinics. **Judgment: Low** | Primary outcome (daily visits/clinic) and prespecified stratifications were reported; sensitivity (Siedner et al., 2020)analyses were described, with some additions during peer review acknowledged. Absence of preregistered analysis plan leaves room for selective emphasis. **Judgment: Moderate** | Moderate |

**Study 20(Observational)**

| **Study ID (Author, Year)** | **Tool Used** | **Confounding** | **Selection of participants** | **Classification of interventions/exposures** | **Deviations from intended interventions** | **Missing data** | **Measurement of outcomes** | **Selection of the reported result** | **Overall RoB** |
| --- | --- | --- | --- | --- | --- | --- | --- | --- | --- |
| (Sisti et al., 2021) | ROBINS-I | Ecological, facility-level comparisons without individual-level adjustment; contextual factors (regional incidence, testing access, facility policies) could confound associations with crowding and facility type. **Judgment: Serious** | Inclusion based on facility managers’ survey participation across national reception centres; participation varied by region and facility type, creating potential selection differences between included and non-included centres. **Judgment: Moderate** | Exposures and facility characteristics (facility type, accommodation, saturation index, region) defined a priori using standardized categories and protocols. **Judgment: Low** | Observational survey of routine practice; no investigator-assigned interventions and no manipulation of adherence beyond standard public health guidance. **Judgment: Low** | Non-participating facilities and unobserved periods were not imputed; analyses restricted to responding centres with available data. **Judgment: Moderate** | Outcomes (suspected/confirmed cases, hospitalisation, deaths) classified per national definitions but reported by facility managers, introducing potential variability in ascertainment across sites. **Judgment: Moderate** | Variables, comparisons, and statistical tests were pre-specified in the methods; absence of preregistered analysis plan leaves scope for selective emphasis among multiple stratifications. **Judgment: Moderate** | Serious |

**Study 21(RCT)**

| **Study ID (Author, Year)** | **Tool Used** | **Randomization process** | **Deviations from intended interventions** | **Missing outcome data** | **Measurement of the outcome** | **Selection of the reported result** | **Overall RoB** |
| --- | --- | --- | --- | --- | --- | --- | --- |
| (Thiem et al., 2025) | RoB 2 | **Low.** Randomisation used a computer-generated sequence with age stratification; participants and outcome assessors were masked, and allocation was implemented by unmasked pharmacy/vaccination staff, indicating adequate concealment.  21 | **Low.** The trial was observer-blind with participants and assessors masked; vaccines were administered per protocol by unmasked vaccinators, and no study-driven deviations affecting outcome ascertainment were described.  21 | **Low.** Follow-up procedures and analysis populations were prespecified; attrition was limited and reasons were reported, with no indication that missingness depended on true outcome status.  21 | **Low.** Outcomes were measured using validated, standardized laboratory assays (ELISA, pseudovirus and live-virus neutralization, AIM) with masked assessors, reducing the likelihood of differential measurement.  21 | **Low.** Primary and secondary outcomes were prespecified and the protocol was registered (ClinicalTrials.gov); reporting follows the stated plan without selective emphasis on alternative analyses.  21 | **Low** |

**Study 22(RCT)**

| **Study ID (Author, Year)** | **Tool Used** | **Randomization process** | **Deviations from intended interventions** | **Missing outcome data** | **Measurement of the outcome** | **Selection of the reported result** | **Overall RoB** |
| --- | --- | --- | --- | --- | --- | --- | --- |
| (Voysey et al., 2021) | RoB 2 | **Low.** Randomisation used pre-generated lists with secure implementation; in some sites allocation was held by an unmasked pharmacist while participants and other staff remained masked, supporting adequate concealment. | **Low.** Trials were observer-blind (one double-blind) with vaccines prepared out of sight and syringes masked; an active comparator was used to minimise unmasking, reducing risk of deviations related to knowledge of assignment. | **Low.** Analysis populations and exclusion criteria were prespecified, with reasons described in protocol and profiles, indicating low risk that missingness depended on true outcomes. | **Low.** Primary outcomes were based on laboratory-confirmed symptomatic disease, with case ascertainment procedures detailed and review by a masked endpoint committee, limiting differential measurement. | **Low.** A pooled statistical analysis plan was finalised with regulators before data lock; trials were registered and analyses prespecified, reducing selective reporting concerns. | **Low** |

**Study 23(Observational)**

| **Study ID (Author, Year)** | **Tool Used** | **Confounding** | **Selection of participants** | **Classification of interventions/exposures** | **Deviations from intended interventions** | **Missing data** | **Measurement of outcomes** | **Selection of the reported result** | **Overall RoB** |
| --- | --- | --- | --- | --- | --- | --- | --- | --- | --- |
| (Wachtler et al., 2024) | ROBINS-I | **Moderate.** A DAG defined a minimal sufficient adjustment set and models incorporated key covariates with survey weights and mediation analyses, which reduces confounding. Residual and time-varying confounding remain plausible in a cross-sectional observational design. | **Moderate.** Participants were drawn from a working-age survey cohort with mail recruitment and weighting for sampling and non-response. Selection related to employment and participation could still bias associations despite weighting. | **Low.** Education was classified using standardized ISCED categories and work-from-home frequency was predefined, with sensitivity analyses on categorization. These definitions are clear and likely independent of outcomes at the time of ascertainment. | **Low.** The study observed routine conditions without study-mandated changes to behavior or care pathways. There is no indication that knowledge of exposure altered subsequent conduct relevant to outcomes. | **Moderate.** Non-response and item missingness were addressed through weighting and analytic restrictions, but some differential missingness cannot be excluded. The extent and patterns of missingness may still influence estimates. | **Moderate.** Infection status combined self-report and serology on dried blood spots using validated assays with adapted cut-points, which mitigates but does not eliminate misclassification. Timing of infection relative to exposure measurement may introduce error. | **Low.** Questions, models, and sensitivity analyses were prespecified via a DAG-based plan with STROBE-aligned reporting and supplementary materials. This reduces risk of selective reporting. | **Moderate** |

**Study 24(RCT)**

| **Study ID (Author, Year)** | **Tool Used** | **Randomization process** | **Deviations from intended interventions** | **Missing outcome data** | **Measurement of the outcome** | **Selection of the reported result** | **Overall RoB** |
| --- | --- | --- | --- | --- | --- | --- | --- |
| (Williams et al., 2024) | RoB 2 | **Low.** The study sits within COV002, a single-blind randomised controlled phase 2/3 trial using a licensed comparator with secure allocation procedures; blinding was maintained except where unblinding was protocol-directed. | **Some concerns.** Although trial conduct followed a prespecified protocol for weekly self-swabbing, external programmatic unblinding and post-infection circumstances could influence participant behaviours related to procedures, creating potential deviations linked to knowledge of assignment. | **Some concerns.** The paper acknowledges incomplete return of scheduled swabs and describes analytic handling and sensitivity checks; however, adherence varied over time and context, leaving uncertainty about whether missingness depended on true outcome status. | **Low.** Asymptomatic infections were identified via standardized RT-PCR on self-collected swabs, with endpoint adjudication by a masked independent panel and uniform laboratory procedures. | **Low.** Outcomes, analysis populations, and procedures were described in protocol materials, with a prespecified analysis framework and detailed methods reducing risk of selective reporting. | **Some concerns** |

Aggarwal, N. R., Nordwall, J., Braun, D. L., Chung, L., Coslet, J., Der, T., Eriobu, N., Ginde, A. A., Hayanga, A. J., Highbarger, H., Holodniy, M., Horcajada, J. P., Jain, M. K., Kim, K., Laverdure, S., Lundgren, J., Natarajan, V., Nguyen, H. H., Pett, S. L.,…Reilly, C. (2024). Viral and Host Factors Are Associated With Mortality in Hospitalized Patients With COVID-19. *Clin Infect Dis*, *78*(6), 1490-1503. <https://doi.org/10.1093/cid/ciad780>

Ambrose, N., Amin, A., Anderson, B., Bertagnolli, M., Campion, F., Chow, D., Danan, R., D'Arinzo, L., Drews, A., Erlandson, K., Fitzgerald, K., Gaspar, F., Gong, C., Hanna, G., Hawley, H., Jones, S., Lopansri, B., Mullen, T., Musser, J.,…Yttri, J. (2023). The Influence of Social Determinants on Receiving Outpatient Treatment with Monoclonal Antibodies, Disease Risk, and Effectiveness for COVID-19. *J Gen Intern Med*, *38*(16), 3472-3481. <https://doi.org/10.1007/s11606-023-08324-y>

Ataguba, J. E., Birungi, C., Cunial, S., & Kavanagh, M. (2023). Income inequality and pandemics: insights from HIV/AIDS and COVID-19-a multicountry observational study. *BMJ Glob Health*, *8*(9). <https://doi.org/10.1136/bmjgh-2023-013703>

Berthaud, V., Creech, C. B., Rostad, C. A., Carr, Q., de Leon, L., Dietrich, M., Gupta, A., Javita, D., Nachman, S., Pinninti, S., Rathore, M., Rodriguez, C. A., Luzuriaga, K., Towner, W., Yeakey, A., Brown, M., Zhao, X., Deng, W., Xu, W.,…Schnyder Ghamloush, S. (2024). Safety and Immunogenicity of an mRNA-1273 Booster in Children. *Clin Infect Dis*, *79*(6), 1524-1532. <https://doi.org/10.1093/cid/ciae420>

Bhattacharyya, R., Burman, A., Singh, K., Banerjee, S., Maity, S., Auddy, A., Rout, S. K., Lahoti, S., Panda, R., & Baladandayuthapani, V. (2022). Role of multiresolution vulnerability indices in COVID-19 spread in India: a Bayesian model-based analysis. *BMJ Open*, *12*(11), e056292. <https://doi.org/10.1136/bmjopen-2021-056292>

Bradbury, C. A., Lawler, P. R., Stanworth, S. J., McVerry, B. J., McQuilten, Z., Higgins, A. M., Mouncey, P. R., Al-Beidh, F., Rowan, K. M., Berry, L. R., Lorenzi, E., Zarychanski, R., Arabi, Y. M., Annane, D., Beane, A., van Bentum-Puijk, W., Bhimani, Z., Bihari, S., Bonten, M. J. M.,…Gordon, A. C. (2022). Effect of Antiplatelet Therapy on Survival and Organ Support-Free Days in Critically Ill Patients With COVID-19: A Randomized Clinical Trial. *Jama*, *327*(13), 1247-1259. <https://doi.org/10.1001/jama.2022.2910>

Bravo, L., Smolenov, I., Han, H. H., Li, P., Hosain, R., Rockhold, F., Clemens, S. A. C., Roa, C., Jr., Borja-Tabora, C., Quinsaat, A., Lopez, P., López-Medina, E., Brochado, L., Hernández, E. A., Reynales, H., Medina, T., Velasquez, H., Toloza, L. B., Rodriguez, E. J.,…Clemens, R. (2022). Efficacy of the adjuvanted subunit protein COVID-19 vaccine, SCB-2019: a phase 2 and 3 multicentre, double-blind, randomised, placebo-controlled trial. *Lancet*, *399*(10323), 461-472. <https://doi.org/10.1016/s0140-6736(22)00055-1>

Gonçalves, B. P., Hall, M., Jassat, W., Balan, V., Murthy, S., Kartsonaki, C., Semple, M. G., Rojek, A., Baruch, J., Reyes, L. F., Dasgupta, A., Dunning, J., Citarella, B. W., Pritchard, M., Martín-Quiros, A., Sili, U., Baillie, J. K., Aryal, D., Arabi, Y.,…Olliaro, P. L. (2022). An international observational study to assess the impact of the Omicron variant emergence on the clinical epidemiology of COVID-19 in hospitalised patients. *Elife*, *11*. <https://doi.org/10.7554/eLife.80556>

Heath, P. T., Galiza, E. P., Baxter, D. N., Boffito, M., Browne, D., Burns, F., Chadwick, D. R., Clark, R., Cosgrove, C. A., Galloway, J., Goodman, A. L., Heer, A., Higham, A., Iyengar, S., Jeanes, C., Kalra, P. A., Kyriakidou, C., Bradley, J. M., Munthali, C.,…Toback, S. (2023). Safety and Efficacy of the NVX-CoV2373 Coronavirus Disease 2019 Vaccine at Completion of the Placebo-Controlled Phase of a Randomized Controlled Trial. *Clin Infect Dis*, *76*(3), 398-407. <https://doi.org/10.1093/cid/ciac803>

Impact of the COVID-19 pandemic on patients with paediatric cancer in low-income, middle-income and high-income countries: a multicentre, international, observational cohort study. (2022). *BMJ Open*, *12*(4), e054690. <https://doi.org/10.1136/bmjopen-2021-054690>

Jennings, K., Lembani, M., Hesseling, A. C., Mbula, N., Mohr-Holland, E., Mudaly, V., Smith, M., Osman, M., & Meehan, S. A. (2024). A decline in tuberculosis diagnosis, treatment initiation and success during the COVID-19 pandemic, using routine health data in Cape Town, South Africa. *PLoS One*, *19*(9), e0310383. <https://doi.org/10.1371/journal.pone.0310383>

López-Macías, C., Torres, M., Armenta-Copca, B., Wacher, N. H., Castro-Castrezana, L., Colli-Domínguez, A. A., Rivera-Hernández, T., Torres-Flores, A., Damián-Hernández, M., Ramírez-Martínez, L., la Rosa, G. P., Rojas-Martínez, O., Suárez-Martínez, A., Peralta-Sánchez, G., Carranza, C., Juárez, E., Zamudio-Meza, H., Carreto-Binaghi, L. E., Viettri, M.,…Lozano-Dubernard, B. (2025). Phase II study on the safety and immunogenicity of single-dose intramuscular or intranasal administration of the AVX/COVID-12 "Patria" recombinant Newcastle disease virus vaccine as a heterologous booster against COVID-19 in Mexico. *Vaccine*, *43*(Pt 2), 126511. <https://doi.org/10.1016/j.vaccine.2024.126511>

López-Macías, C., Torres, M., Armenta-Copca, B., Wacher, N. H., Galindo-Fraga, A., Castro-Castrezana, L., Colli-Domínguez, A. A., Cervantes-Trujano, E., Rucker-Joerg, I. E., Lozano-Patiño, F., Rivera-Alcocer, J. J., Simón-Campos, A., Sánchez-Campos, E. A., Aguirre-Rivero, R., Muñiz-Carvajal, A. J., Del Carpio-Orantes, L., Márquez-Díaz, F., Rivera-Hernández, T., Torres-Flores, A.,…Lozano-Dubernard, B. (2025). Phase 2/3 study evaluating safety, immunogenicity, and noninferiority of single booster dose of AVX/COVID-12 vaccine. *Sci Adv*, *11*(26), eadq2887. <https://doi.org/10.1126/sciadv.adq2887>

Mayland, C. R., Hughes, R., Lane, S., McGlinchey, T., Donnellan, W., Bennett, K., Hanna, J., Rapa, E., Dalton, L., & Mason, S. R. (2021). Are public health measures and individualised care compatible in the face of a pandemic? A national observational study of bereaved relatives' experiences during the COVID-19 pandemic. *Palliat Med*, *35*(8), 1480-1491. <https://doi.org/10.1177/02692163211019885>

Mediavilla, R., Felez-Nobrega, M., McGreevy, K. R., Monistrol-Mula, A., Bravo-Ortiz, M. F., Bayón, C., Giné-Vázquez, I., Villaescusa, R., Muñoz-Sanjosé, A., Aguilar-Ortiz, S., Figueiredo, N., Nicaise, P., Park, A. L., Petri-Romão, P., Purgato, M., Witteveen, A. B., Underhill, J., Barbui, C., Bryant, R.,…Ayuso-Mateos, J. L. (2023). Effectiveness of a mental health stepped-care programme for healthcare workers with psychological distress in crisis settings: a multicentre randomised controlled trial. *BMJ Ment Health*, *26*(1). <https://doi.org/10.1136/bmjment-2023-300697>

Nice, K. A., Thompson, J., Zhao, H., Seneviratne, S., Zapata-Diomedi, B., Garcia, L., Hunter, R. F., Reis, R. S., Hallal, P. C., Millett, C., Wang, R., & Stevenson, M. (2025). Effects of city design on transport mode choice and exposure to health risks during and after a crisis: a retrospective observational analysis. *Lancet Planet Health*, *9*(6), e467-e479. <https://doi.org/10.1016/s2542-5196(25)00088-9>

Puertas-Gonzalez, J. A., Mariño-Narvaez, C., Romero-Gonzalez, B., Sanchez-Perez, G. M., & Peralta-Ramirez, M. I. (2022). Online cognitive behavioural therapy as a psychological vaccine against stress during the COVID-19 pandemic in pregnant women: A randomised controlled trial. *J Psychiatr Res*, *152*, 397-405. <https://doi.org/10.1016/j.jpsychires.2022.07.016>

Reyes, L. F., Garcia-Gallo, E., Murthy, S., Fuentes, Y. V., Serrano, C. C., Ibáñez-Prada, E. D., Lee, J., Rojek, A., Citarella, B. W., Gonçalves, B. P., Dunning, J., Rätsep, I., Viñan-Garces, A. E., Kartsonaki, C., Rello, J., Martin-Loeches, I., Shankar-Hari, M., Olliaro, P. L., & Merson, L. (2023). Major adverse cardiovascular events (MACE) in patients with severe COVID-19 registered in the ISARIC WHO clinical characterization protocol: A prospective, multinational, observational study. *J Crit Care*, *77*, 154318. <https://doi.org/10.1016/j.jcrc.2023.154318>

Siedner, M. J., Kraemer, J. D., Meyer, M. J., Harling, G., Mngomezulu, T., Gabela, P., Dlamini, S., Gareta, D., Majozi, N., Ngwenya, N., Seeley, J., Wong, E., Iwuji, C., Shahmanesh, M., Hanekom, W., & Herbst, K. (2020). Access to primary healthcare during lockdown measures for COVID-19 in rural South Africa: an interrupted time series analysis. *BMJ Open*, *10*(10), e043763. <https://doi.org/10.1136/bmjopen-2020-043763>

Sisti, L. G., Di Napoli, A., Petrelli, A., Rossi, A., Diodati, A., Menghini, M., Mirisola, C., & Costanzo, G. (2021). COVID-19 Impact in the Italian Reception System for Migrants during the Nationwide Lockdown: A National Observational Study. *Int J Environ Res Public Health*, *18*(23). <https://doi.org/10.3390/ijerph182312380>

Thiem, V. D., Anh, D. D., Ha, V. H., Van Thom, N., Thang, T. C., Mateus, J., Carreño, J. M., Raghunandan, R., Huong, N. M., Mercer, L. D., Flores, J., Escarrega, E. A., Raskin, A., Thai, D. H., Van Be, L., Sette, A., Innis, B. L., Krammer, F., & Weiskopf, D. (2025). Safety and immunogenicity of an inactivated recombinant Newcastle disease virus vaccine expressing SARS-CoV-2 spike: A randomised, comparator-controlled, phase 2 trial. *Vaccine*, *44*, 126542. <https://doi.org/10.1016/j.vaccine.2024.126542>

Voysey, M., Clemens, S. A. C., Madhi, S. A., Weckx, L. Y., Folegatti, P. M., Aley, P. K., Angus, B., Baillie, V. L., Barnabas, S. L., Bhorat, Q. E., Bibi, S., Briner, C., Cicconi, P., Collins, A. M., Colin-Jones, R., Cutland, C. L., Darton, T. C., Dheda, K., Duncan, C. J. A.,…Pollard, A. J. (2021). Safety and efficacy of the ChAdOx1 nCoV-19 vaccine (AZD1222) against SARS-CoV-2: an interim analysis of four randomised controlled trials in Brazil, South Africa, and the UK. *Lancet*, *397*(10269), 99-111. <https://doi.org/10.1016/s0140-6736(20)32661-1>

Wachtler, B., Beese, F., Demirer, I., Haller, S., Pförtner, T. K., Wahrendorf, M., Grabka, M. M., & Hoebel, J. (2024). Education and pandemic SARS-CoV-2 infections in the German working population - the mediating role of working from home. *Scand J Work Environ Health*, *50*(3), 168-177. <https://doi.org/10.5271/sjweh.4144>

Williams, L. R., Emary, K. R. W., Phillips, D. J., Hay, J., Larwood, J. P. J., Ramasamy, M. N., Pollard, A. J., Grassly, N. C., & Voysey, M. (2024). Implementation and adherence to regular asymptomatic testing in a COVID-19 vaccine trial. *Vaccine*, *42*(21), 126167. <https://doi.org/10.1016/j.vaccine.2024.126167>

The research design presented in the paper involves an exhaustive analysis of different studies using established tools to measure risks and biases. The studies involve a mix of observational cohorts, randomized controlled trials (RCTs), and ecological analysis using tools like ROBINS-I for observational studies and RoB 2 for clinical trials. The assessment considers issues like confounding, participant selection, categorization of interventions or exposures, deviation from scheduled interventions, missing data management, measurement of outcomes, and selection bias in published results. Each study is critically assessed on these areas to determine an overall risk of bias that ranges from low to severe. Data collection methods vary—from consecutive recruitment in hospital cohorts from several countries to blinding and randomization in clinical trials. Outcomes are quantified against mortality, immunogenicity, hospitalization, and psychological scoring as defined by standardized protocols and validated assays. Methodology approach. It also stresses registration of protocols, pre-specification of analysis plans, application of statistical adjustments like propensity scores, and missing information handling by means like multiple imputation to reduce bias and increase the validity of results.
