# Supplementary material for: COVID‐19 Pandemic: A Comprehensive Meta‐Review of Global Impacts, Responses, and Future Preparedness
Source: Clin Respir J. 2025 Nov 21;19(11):e70134. doi: 10.1111/crj.70134 (PMC12635497; doi:10.1111/crj.70134)
Supplement: Supplementary file 4 — Data S4: Supporting information. [file CRJ-19-e70134-s003.docx]

**Group A: Clinical Outcomes (Mortality, Infection, Hospitalization, Adherence, Equity Risks)-15 studies**

**1. Meta-Analysis**

**1. Model Information**

- **Model type:** Random-effects model
- **Number of studies (k):** 15
- **Estimator for tau²:** REML (Restricted Maximum Likelihood)
- **Log-likelihood:** -21.4811
- **Deviance:** 42.9622
- **AIC:** 46.9622
- **BIC:** 48.2403
- **Corrected AIC (AICc):** 48.0531

**2. Heterogeneity**

- **Tau² (between-study variance):** 1.0552 (SE = 0.4219)
- **Tau (√Tau²):** 1.0272
- **I² (proportion of variance due to heterogeneity):** 99.94%
- **H² (total variability / sampling variability):** 1712.96
- **Cochran’s Q-test:** Q(14) = 1668.86, **p < 0.0001**
  - Indicates **very high heterogeneity** among included studies.

**3. Model Results (on log scale)**

- **Pooled estimate:** -0.0551
- **Standard error (SE):** 0.2732
- **Z-value:** -0.2018
- **p-value:** 0.8400 (not statistically significant)
- **95% CI:** -0.5907 to 0.4804

**4. Pooled Effect (Ratio Scale)**

- **Estimate (exp of pooled log estimate):** 0.9463
- **95% CI:** 0.5540 – 1.6167

Interpretation:

- The pooled ratio is **close to 1 (0.95)**, indicating no significant effect.
- Confidence interval is wide and includes 1 → results are **not statistically significant**.
- Very high heterogeneity suggests that effect sizes vary substantially between studies.

**2. Publication bias**

**Regression Test for Funnel Plot Asymmetry**

- **Model:** Weighted regression with multiplicative dispersion
- **Predictor:** Standard error (SE)

**Test Results**

- **Test statistic (t):** 0.6157
- **Degrees of freedom (df):** 13
- **p-value:** 0.5487

**Interpretation:**

- The p-value is **not significant (p > 0.05)**, suggesting that there is **no statistical evidence of funnel plot asymmetry**.
- In practical terms, this means **no strong indication of publication bias** in the included studies.

**Limit Estimate (as SE → 0)**

- **Estimated effect (b):** -0.0833
- **95% Confidence Interval (CI):** -0.1114 to -0.0553

**Interpretation:**

- As the standard error approaches zero (i.e., in very large studies), the pooled effect is estimated to be **-0.0833**.
- Since the CI does not cross 0, this indicates a **slight negative effect** in the asymptotic limit, but the overall test shows **no significant asymmetry**.

**3. Sub-group analysis**

**Table 1. Model Fit and Heterogeneity Statistics**

| **Model (Group)** | **k** | **logLik** | **AIC** | **BIC** | **AICc** | **τ² (SE)** | **τ** | **I² (%)** | **H²** | **R² (%) (explained)** |
| --- | --- | --- | --- | --- | --- | --- | --- | --- | --- | --- |
| Mixed-Effects (Overall) | 15 | –15.95 | 39.91 | 41.85 | 45.62 | 0.6969 (0.3072) | 0.835 | 99.92 | 1302.57 | **33.95** |
| Small Sample | 2 | –0.33 | 4.65 | 0.65 | 16.65 | 0 (0.4036) | 0 | 0.00 | 1.00 | – |
| Medium Sample | 4 | –4.47 | 12.93 | 11.13 | 24.93 | 0.6148 (0.7053) | 0.784 | 75.40 | 4.07 | – |
| Large Sample | 9 | –10.42 | 24.84 | 25.00 | 27.24 | 0.5880 (0.3529) | 0.767 | 96.13 | 25.87 | – |

**Table 2. Model Results and Statistical Tests**

| **Model (Group)** | **Estimate** | **SE** | **z-value** | **p-value** | **95% CI (Lower, Upper)** | **Test for Heterogeneity** | **Notes** |
| --- | --- | --- | --- | --- | --- | --- | --- |
| Mixed-Effects (Overall) | – | – | – | – | – | QE(df=12) = 1629.92, *p* < 0.0001 | QM(df=2) = 8.83, *p* = 0.0121; **R² = 33.95%** |
| • Intercept | 0.6892 | 0.6239 | 1.10 | 0.2693 | –0.534, 1.912 | – | – |
| • Medium (1k–10k) | 0.1451 | 0.7694 | 0.19 | 0.8505 | –1.363, 1.653 | – | – |
| • Large (>10k) | –1.2784 | 0.6860 | –1.86 | 0.0624 | –2.623, 0.066 | – | Borderline (p≈0.06) |
| Small Sample | 0.6934 | 0.3772 | 1.84 | 0.0660 | –0.046, 1.433 | Q(df=1) = 0.07, *p* = 0.791 | No heterogeneity |
| Medium Sample | 0.8164 | 0.4654 | 1.75 | 0.0794 | –0.096, 1.729 | Q(df=3) = 10.04, *p* = 0.018 | Significant heterogeneity |
| Large Sample | –0.5245 | 0.2813 | –1.86 | 0.0622 | –1.076, 0.027 | Q(df=8) = 55.29, *p* < 0.0001 | Strong heterogeneity |

**4. sensitivity analysis**

| **Study Removed** | **Estimate** | **SE** | **z-value** | **p-value** | **CI Lower** | **CI Upper** | **Q** | **Q p-val** | **Tau²** | **I² (%)** | **H²** |
| --- | --- | --- | --- | --- | --- | --- | --- | --- | --- | --- | --- |
| Impact of COVID-19 on paediatric cancer patients (2022) | -0.1901 | 0.2445 | -0.7772 | 0.4370 | -0.6694 | 0.2892 | 1654.03 | <0.001 | 0.7964 | 99.93 | 1392.32 |
| Aggarwal et al. (2024) | -0.1185 | 0.2860 | -0.4142 | 0.6787 | -0.6791 | 0.4421 | 1635.08 | <0.001 | 1.0775 | 99.95 | 1876.69 |
| Ambrose et al. (2023) | 0.0648 | 0.2528 | 0.2565 | 0.7976 | -0.4306 | 0.5603 | 1644.00 | <0.001 | 0.8378 | 99.93 | 1464.07 |
| Bradbury et al. (2022) | -0.0575 | 0.2968 | -0.1939 | 0.8463 | -0.6392 | 0.5242 | 1667.65 | <0.001 | 1.1635 | 99.95 | 2012.78 |
| Bravo et al. (2022) | 0.0230 | 0.2825 | 0.0814 | 0.9352 | -0.5307 | 0.5766 | 1633.12 | <0.001 | 1.0500 | 99.95 | 1830.30 |
| Gonçalves et al. (2022) | -0.0238 | 0.2955 | -0.0805 | 0.9358 | -0.6029 | 0.5553 | 1504.10 | <0.001 | 1.1520 | 99.94 | 1779.07 |
| Heath et al. (2023) | 0.0596 | 0.2600 | 0.2292 | 0.8187 | -0.4500 | 0.5692 | 1614.13 | <0.001 | 0.8823 | 99.94 | 1539.88 |
| Jennings et al. (2024) | -0.0657 | 0.2965 | -0.2215 | 0.8247 | -0.6468 | 0.5155 | 1585.92 | <0.001 | 1.1606 | 99.94 | 1687.31 |
| Mayland et al. (2021) | -0.1141 | 0.2864 | -0.3983 | 0.6904 | -0.6753 | 0.4472 | 1658.52 | <0.001 | 1.0838 | 99.95 | 1892.45 |
| Nice et al. (2025) | -0.0982 | 0.2900 | -0.3387 | 0.7348 | -0.6667 | 0.4702 | 1663.91 | <0.001 | 1.1144 | 99.95 | 1946.27 |
| Reyes et al. (2023) | -0.0798 | 0.2950 | -0.2705 | 0.7868 | -0.6581 | 0.4985 | 467.52 | <0.001 | 1.1485 | 99.84 | 632.65 |
| Sisti et al. (2021) | -0.0969 | 0.2909 | -0.3330 | 0.7392 | -0.6669 | 0.4732 | 1662.27 | <0.001 | 1.1191 | 99.95 | 1953.66 |
| Voysey et al. (2021) | 0.0276 | 0.2801 | 0.0987 | 0.9214 | -0.5213 | 0.5765 | 1641.77 | <0.001 | 1.0323 | 99.94 | 1801.37 |
| Wachtler et al. (2024) | -0.0977 | 0.2907 | -0.3360 | 0.7369 | -0.6674 | 0.4721 | 1662.20 | <0.001 | 1.1176 | 99.95 | 1951.10 |
| Williams et al. (2024) | -0.0496 | 0.2971 | -0.1671 | 0.8673 | -0.6319 | 0.5327 | 881.28 | <0.001 | 1.1654 | 99.80 | 499.93 |

**Group B. Immunogenicity Outcomes (Neutralizing Antibodies, Seroconversion, GMT/GMR)-4 studies**

- 1. **Meta-analysis**

**Model Fit and Heterogeneity**

| **Statistic** | **Value** |
| --- | --- |
| logLik | -4.7220 |
| Deviance | 9.4440 |
| AIC | 13.4440 |
| BIC | 13.0275 |
| AICc | 17.4440 |

| **Heterogeneity Measure** | **Value (SE)** | **Interpretation** |
| --- | --- | --- |
| τ² (between-study variance) | 0.2558 (0.1607) | Moderate variance |
| τ (√τ²) | 0.5057 | SD of true effects |
| I² | 95.95% | Very high heterogeneity |
| H² | 24.69 | Large ratio of total to sampling variability |

**Test for Heterogeneity**

- Q(df = 6) = 251.48, p < 0.0001 → strong evidence of heterogeneity.

**Model Results**

| **Parameter** | **Estimate** | **SE** | **z-value** | **p-value** | **95% CI (Lower–Upper)** | **Significance** |
| --- | --- | --- | --- | --- | --- | --- |
| Overall Effect | 0.7684 | 0.1997 | 3.8471 | 0.0001 | 0.3769 – 1.1599 | *** |

**Interpretation**

- The pooled estimate is **0.77 (95% CI: 0.38–1.16)**, statistically significant (p = 0.0001).
- **Heterogeneity is extremely high (I² ≈ 96%)**, indicating large variation across studies.
- While the overall effect is positive and significant, the high heterogeneity suggests that moderator or subgroup analyses are needed for more reliable interpretation.
  1. **Publication bias**

**Regression Test for Funnel Plot Asymmetry**

- **Model:** Weighted regression with multiplicative dispersion
- **Predictor:** Standard error

**Test Results**

- **t-value:** 1.1871
- **Degrees of freedom (df):** 5
- **p-value:** 0.2885

**Limit Estimate (as SE → 0)**

- **Bias estimate (b):** -0.0383
- **95% CI:** -1.4005 to 1.3239

**Interpretation**

- The test for funnel plot asymmetry is **not statistically significant** (p = 0.2885).
- This suggests **no strong evidence of small-study effects or publication bias**.
- The bias estimate (b = -0.0383) is close to zero, and the 95% CI is wide and crosses zero, indicating high uncertainty.
  1. **Sensitivity analysis**

| **Excluded Study** | **Estimate** | **SE** | **z-value** | **p-value** | **95% CI (Lower–Upper)** |
| --- | --- | --- | --- | --- | --- |
| Berthaud et al. (2024) | 0.6502 | 0.1945 | 3.34 | 0.00083 | 0.2691 – 1.0314 |
| López-Macías et al. (2025a) | 0.6841 | 0.2034 | 3.36 | 0.00077 | 0.2854 – 1.0828 |
| López-Macías et al. (2025b) | 0.9058 | 0.1652 | 5.48 | <0.0001 | 0.5819 – 1.2296 |
| Thiem et al. (2025, 18–59 y, 3 µg) | 0.8305 | 0.2282 | 3.64 | 0.00027 | 0.3833 – 1.2777 |
| Thiem et al. (2025, 18–59 y, 6 µg) | 0.8074 | 0.2345 | 3.44 | 0.00058 | 0.3478 – 1.2671 |
| Thiem et al. (2025, ≥60 y, 3 µg) | 0.7616 | 0.2368 | 3.22 | 0.00130 | 0.2974 – 1.2258 |
| Thiem et al. (2025, ≥60 y, 6 µg) | 0.7418 | 0.2340 | 3.17 | 0.00153 | 0.2831 – 1.2004 |

**Heterogeneity Measures**

| **Excluded Study** | **Q** | **p(Q)** | **Tau²** | **I² (%)** | **H²** |
| --- | --- | --- | --- | --- | --- |
| Berthaud et al. (2024) | 100.07 | 5.1e-20 | 0.2011 | 93.88 | 16.33 |
| López-Macías et al. (2025a) | 243.97 | 1.1e-50 | 0.2363 | 96.27 | 26.78 |
| López-Macías et al. (2025b) | 60.33 | 1.0e-11 | 0.1388 | 90.03 | 10.03 |
| Thiem et al. (2025, 18–59 y, 3 µg) | 250.70 | 3.9e-52 | 0.2870 | 96.48 | 28.43 |
| Thiem et al. (2025, 18–59 y, 6 µg) | 251.39 | 2.8e-52 | 0.3046 | 96.69 | 30.25 |
| Thiem et al. (2025, ≥60 y, 3 µg) | 245.00 | 6.5e-51 | 0.3111 | 96.76 | 30.88 |
| Thiem et al. (2025, ≥60 y, 6 µg) | 239.41 | 1.0e-49 | 0.3032 | 96.68 | 30.12 |

**Key Points**

- **All leave-one-out estimates remain significant**, confirming robustness.
- Excluding *López-Macías et al. (2025b)* yields the highest pooled effect (0.91).
- Excluding *Berthaud et al. (2024)* lowers the effect most (0.65).
- Heterogeneity remains **very high across all analyses (I² > 90%)**, though slightly reduced when excluding *López-Macías et al. (2025b)*.
  1. **Subgroup analysis (sample size)**

**Model Fit Statistics**

- **Number of studies (k):** 4
- **Log-likelihood (logLik):** -0.2284
- **Deviance:** 0.4568
- **Akaike Information Criterion (AIC):** 8.4568
- **Bayesian Information Criterion (BIC):** 0.4568
- **Corrected AIC (AICc):** 48.4568

**Heterogeneity Estimates**

- **Tau² (residual between-study variance):** 0.0815 (SE = 0.1307)
- **Tau (√Tau²):** 0.2854
- **I² (residual heterogeneity / unexplained variability):** 88.12%
- **H² (ratio of total to sampling variability):** 8.42
- **R² (heterogeneity explained by moderators):** 82.65%

**Test for Residual Heterogeneity**

- **QE(df = 1):** 8.4199
- **p-value:** 0.0037 (significant residual heterogeneity remains, but much reduced compared to the overall model)

**Moderator Analysis**

- **Test of Moderators (QM, df = 2):** 13.6475
- **p-value:** 0.0011 (**significant moderators**)

This indicates that sample group size (Small vs. Moderate vs. Large) explains a substantial portion of heterogeneity (R² = 82.65%).

**Model Results**

| **Parameter** | **Estimate** | **SE** | **z-value** | **p-value** | **95% CI (Lower–Upper)** | **Significance** |
| --- | --- | --- | --- | --- | --- | --- |
| Intercept (Small, <100) | 1.4810 | 0.4509 | 3.2849 | 0.0010 | 0.5973 – 2.3647 | ** |
| SampleGroup Moderate (100–499) | -0.3056 | 0.4994 | -0.6120 | 0.5405 | -1.2844 – 0.6731 | n.s. |
| SampleGroup Large (≥500) | -1.5220 | 0.5364 | -2.8372 | 0.0046 | -2.5734 – -0.4706 | ** |

**Interpretation**

- **Intercept (Small studies <100):** Effect estimate = 1.48, statistically significant (p = 0.0010).
- **Moderate studies (100–499):** Effect not significantly different from small studies (p = 0.5405).
- **Large studies (≥500):** Show a significantly **lower effect** compared to small studies (estimate = -1.52, p = 0.0046).
- Overall, subgroup analysis indicates that **sample size is a significant moderator**, explaining ~83% of between-study heterogeneity.

**Group C. Psychosocial & Health Service Outcomes (Continuous & ITS) 5-studies**

1. **Meta analysis**

Model Fit Statistics

| Statistic | Value |
| --- | --- |
| logLik | -16.9827 |
| Deviance | 33.9654 |
| AIC | 37.9654 |
| BIC | 36.7379 |
| AICc | 49.9654 |

Heterogeneity Estimates

| Measure | Value (SE) | Interpretation |
| --- | --- | --- |
| τ² (between-study variance) | 21.9993 (18.8963) | Very large variance |
| τ (√τ²) | 4.6903 | Large SD of true effects |
| I² | 95.84% | Extremely high heterogeneity |
| H² | 24.02 | Very large ratio of total to sampling variability |

Test for Heterogeneity

- Q(df = 4) = 80.17, p < 0.0001 → Strong evidence of substantial heterogeneity.

Model Results

| Parameter | Estimate | SE | z-value | p-value | 95% CI (Lower–Upper) | Significance |
| --- | --- | --- | --- | --- | --- | --- |
| Overall Effect | -1.0260 | 2.4048 | -0.4266 | 0.6696 | -5.7393 – 3.6873 | n.s. |

Interpretation

- The pooled effect size is -1.03 (95% CI: -5.74 to 3.69).
- This result is not statistically significant (p = 0.6696).
- The heterogeneity is extremely high (I² = 95.84%), suggesting wide variability among studies.
- Because of the very large τ² (≈22) and wide CI, the true effects likely differ substantially across studies.

1. **Publication bias**

**Regression Test for Funnel Plot Asymmetry**

- **Model:** Mixed-effects meta-regression
- **Predictor:** Standard error

**Test Results**

- **z-value:** 2.2002
- **p-value:** 0.0278 (statistically significant)

**Limit Estimate (as SE → 0)**

- **Bias estimate (b):** -3.2406
- **95% CI:** -8.5096 to 2.0284

**Interpretation**

- The test suggests **statistically significant evidence of funnel plot asymmetry** (p = 0.0278).
- The negative bias estimate (b = -3.24) indicates that **smaller studies may be reporting more extreme effects**, raising concerns about **possible small-study effects or publication bias**.
- However, the 95% CI around the bias estimate is wide (crossing zero), meaning the **magnitude and direction of the bias remain uncertain**.

1. **Sensitivity analysis**

| **Excluded Study** | **Estimate** | **SE** | **95% CI (Lower–Upper)** | **p-value** | **τ²** | **I² (%)** | **H²** | **Q** | **Q p-value** |
| --- | --- | --- | --- | --- | --- | --- | --- | --- | --- |
| Ataguba et al. (2023) | –1.14 | 2.40 | –5.85 to 3.56 | 0.634 | 21.92 | 96.8 | 31.58 | 75.17 | <0.001 |
| Bhattacharyya et al. (2022) | –1.37 | 3.38 | –7.99 to 5.25 | 0.685 | 32.75 | 94.0 | 16.53 | 64.82 | <0.001 |
| Mediavilla et al. (2023) | –2.85 | 2.23 | –7.22 to 1.51 | 0.200 | 13.84 | 93.1 | 14.52 | 63.90 | <0.001 |
| Puertas-Gonzalez et al. (2022) | –0.63 | 3.34 | –7.18 to 5.92 | 0.850 | 32.77 | 97.4 | 38.86 | 78.78 | <0.001 |
| Siedner et al. (2020) | 0.97 | 1.80 | –2.55 to 4.49 | 0.589 | 8.48 | 85.9 | 7.08 |  |  |

1. **Subgroup analysis (sample size)**

| **Parameter** | **Estimate** | **SE** | **z-value** | **p-value** | **95% CI (Lower–Upper)** | **Significance** |
| --- | --- | --- | --- | --- | --- | --- |
| Intercept | –0.40 | 0.97 | –0.41 | 0.682 | –2.30 to 1.51 | n.s. |
| Sample Group (Medium) | 4.85 | 1.88 | 2.58 | 0.010 | 1.17 to 8.53 | ** |
| Sample Group (Large) | –6.70 | 1.73 | –3.87 | <0.001 | –10.09 to –3.31 | *** |

**Model Fit and Heterogeneity**

- **Residual heterogeneity (τ²):** 1.21 (SE = 3.46)
- **I² (unaccounted variability):** 32.7%
- **H²:** 1.49
- **R² (heterogeneity explained by moderators):** 94.5%
- **Test for residual heterogeneity:** Q(df = 2) = 6.78, p = 0.034
- **Test of moderators (Sample Group):** QM(df = 2) = 30.01, p < 0.001
